# Solvent-free construction of Cr(iii)-sulfonate coordination polymers†

**DOI:** 10.1039/d5sc03014e

**Published:** 2025-05-31

**Authors:** Fan Yang, Xiang-Jing Kong, Tao He, Zhengqing Zhang, Ke Wang, Honglin Du, Guohong Cai, Jing Ju, Xiaoge Wang, Jian-Rong Li, Junliang Sun, Chongli Zhong

**Affiliations:** a State Key Laboratory of Advanced Separation Membrane Materials, School of Chemistry and Chemical Engineering, Tiangong University Tianjin 300387 P. R. China zhongchongli@tiangong.edu.cn; b Beijing Key Laboratory for Green Catalysis and Separation and Department of Chemical Engineering, College of Materials Science and Engineering, Beijing University of Technology Beijing China jrli@bjut.edu.cn; c College of Chemistry and Molecular Engineering, Beijing National Laboratory for Molecular Sciences, Peking University Beijing China wang_wxg@pku.edu.cn junliang.sun@pku.edu.cn

## Abstract

Constructing sulfonate coordination polymers (CPs) with high stability remains a significant challenge due to the relatively weak coordination ability of the sulfonate group, especially when paired with highly inert Cr^3+^ ions. In this study, we designed solvent-free methods to enhance Cr(iii)-sulfonate coordination and further advance its reticular chemistry. For the first time, two Cr(iii)-sulfonate CPs, TGU-9 and TGU-10, were successfully constructed, along with two supramolecules, TGU-7 and TGU-8. All structures were elucidated using the 3D electron diffraction technique. Through solvent-free methods, Cr(iii)-sulfonate coordination was achieved by a double displacement reaction between Cr salts and –SO_3_H groups. In particular, this method resulted in a counterintuitive coordination reversal from –COO^−^ > –SO_3_^−^ to –SO_3_^−^ > –COO^−^. Reaction mechanism analysis revealed that the higher acidity of the –SO_3_H group, compared to the –COOH group, leads to its preferential deprotonation, thereby facilitating the kinetics of Cr-sulfonate self-assembly. Furthermore, both TGU-9 and TGU-10 exhibited exceptional long-term stability under ambient conditions and over a wide pH range. They also showed high proton conductivity exceeding 10^−2^ S cm^−1^, ranking in the top two among the reported sulfonate CPs. The designed solvent-free method demonstrated a generally applicable and simple strategy in designing novel metal–ligand coordination and constructing reticular chemistry, beyond the limitations of conventional solvent-based methods.

## Abstract 2

Solvent-free synthesis enhances sulfonate coordination, enabling the first assembly of Cr(iii)-SO_3_ coordination polymers with high stability and proton conductivity and reversing the coordination hierarchy from –SO_3_^−^ < –COO^−^ to –SO_3_^−^ > –COO^−^.

## Graphical abstract

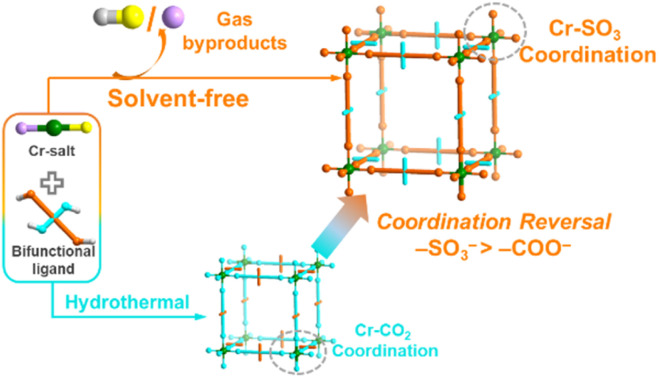

## Introduction

Solvent-free reactions have garnered widespread recognition as a promising alternative to solution-based chemistry.^1–4^ As the name suggests, this methodology not only eliminates the use of bulk solvents, thereby significantly reducing waste generation, but also creates a distinctive reaction environment.^5,6^ Within this unique setting, chemists gain access to distinct synthetic strategies to explore novel chemistry and materials science that were previously unattainable in solution-based systems.^7–12^

Over the past two decades, coordination polymers (CPs) including metal–organic frameworks (MOFs) have undergone remarkable progress in both synthesis and application research.^13,14^ Among the various types of CPs, porous metal sulfonates are recognized as an important subclass due to their structural diversity and the abundance of polar sites within their pores, which enable a wide range of promising applications,^15–17^ such as proton conduction.^18–26^ However, compared to the rapid progress of CPs, the advancement of sulfonate CPs is significantly slower. This lag is primarily attributed to the relatively weak coordination ability of sulfonates with metal ions in solution.^15,16,27–30^ In some instances, sulfonate ligands exhibit even lower coordination affinity than the solvents, such as water.^28,30^ Therefore, the solvent-free synthetic approach holds great potential for the discovery of new sulfonate-based CPs, although it has yet to be investigated.

**Fig. 1:**
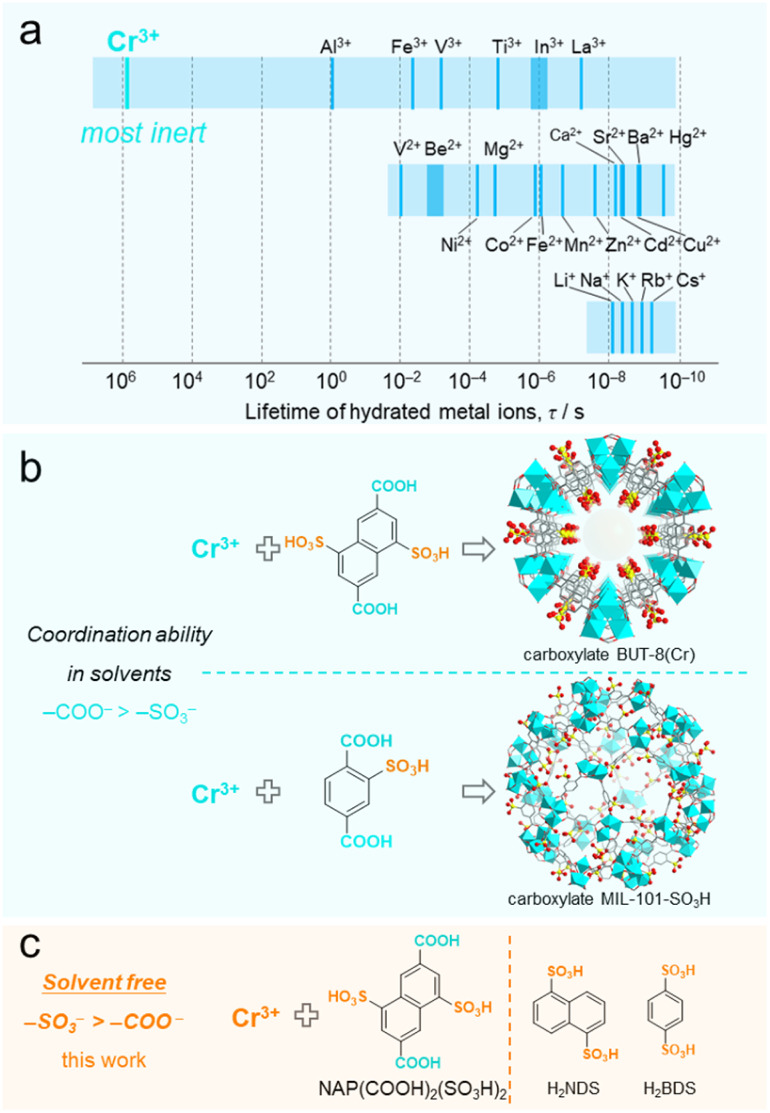
Concept illustration of the solvent-free Cr–SO_3_ coordination based on the inertness of the Cr^3+^ ion and the low coordination ability of the sulfonate group in the solvent. (a) Mean lifetimes of a water molecule (*τ*_H_2_O_) in hydrated metal ions.^39–42^ (b) The representative Cr-carboxylate BUT-8 and MIL-101-SO_3_H with uncoordinated –SO_3_H/–SO_3_^−^ groups developed in solvents.^43,44^ (c) Proposed coordination between the Cr^3+^ ion and the sulfonate group *via* solvent-free methods in this work.

With regard to the metal center, adopting the solvent-free approach to construct Cr(iii)-sulfonate coordinated CPs is highly representative. This is due to the following two reasons: (1) the Cr(iii)-based CPs, characterized by the high inertness of Cr(iii)–O bonds, represent one of the most stable categories of all CPs/MOFs;^31–38^ (2) Cr^3+^ ions have the inherent ability to form the most stable solvated complexes in solution among the non-noble metal ions (Fig. 1a),^39–42^ where it is challenging to substitute the coordinated solvent molecules with organoligands, especially sulfonate ligands. For the latter, our previously reported BUT-8(Cr) clearly illustrates the difficulty in substitution (Fig. 1b).^43^ BUT-8(Cr) is constructed from the bifunctional ligand 4,8-disulfonaphthalene-2,6-dicarboxylic acid (NAP(COOH)_2_(SO_3_H)_2_) and Cr^3+^ ions in *N*,*N*-dimethylformamide solution. In this MOF, the sulfonate groups are unable to coordinate with Cr^3+^ ions, while the carboxylate groups preferentially bind to the metal centers. The similar difficulty in coordination between Cr^3+^ and the sulfonate group is also suggested by the hydrothermally synthesized Cr-carboxylate MIL-101-SO_3_H, in which the –SO_3_H/–SO_3_^−^ groups on the carboxylate ligand remain uncoordinated.^44^ To date, no Cr-sulfonate CPs or MOFs have been reported except for only one supramolecular structure, which was obtained through an over nine-day slow-kinetic process to substitute the water molecules in Cr(H_2_O)_6_^3+^ ions.^45^ Therefore, overcoming the solvent-related constraints to construct Cr-sulfonate frameworks using solvent-free synthetic strategies is of high chemical interest.

In this study, we reported a solvent-free method for synthesizing Cr-sulfonate coordination frameworks. 3D electron diffraction elucidation showed that TGU-9 (TGU = Tiangong University) and TGU-10 were Cr(iii)-sulfonate coordinated CPs, while TGU-7 and TGU-8 were Cr(iii)-sulfonate coordination supramolecules. Besides, with the same –SO_3_H/–COOH bifunctional ligand employed in the synthesis of TGU-9, a Cr(iii)-carboxylate TGU-11 was obtained by the hydrothermal method. Notably, the designed solvent-free method was effective in enhancing Cr–SO_3_ coordination and even facilitated a counterintuitive reversal of the conventional coordination preference from –COO^−^ > –SO_3_^−^ to –SO_3_^−^ > –COO^−^. Mechanism investigation showed that the double replacement reaction between Cr salts and –SO_3_H groups played a pivotal role in Cr(iii)-sulfonate coordination. Furthermore, TGU-9 and TGU-10 exhibited exceptional long-term stability under environmental conditions and over a wide pH range, as well as the highest proton conductivity among the reported sulfonate CPs.

## Results and discussion

### Synthesis and 3D ED structure analysis

**Fig. 2:**
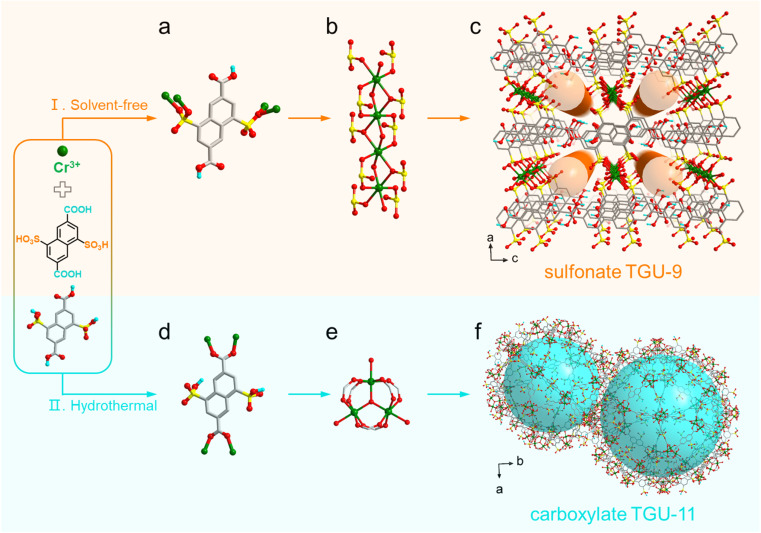
Reversed coordination preference: from Cr-carboxylate coordination in aqueous solution to Cr-sulfonate coordination under solvent-free conditions. (a) Sulfonate coordinated NAP(COOH)_2_(SO_3_)_2_^2−^ linker with four Cr^3+^ ions. (b) 1D Cr–SO_3_ SBU in TGU-9. (c) Sulfonate TGU-9 viewed along the *b* axis. (d) Carboxylate coordinated NAP(COO)_2_(SO_3_H)_2_^2−^ linker with Cr^3+^ ions. (e) Cr_3_ trimer. (f) Carboxylate TGU-11 viewed along the *c* axis. H atoms are omitted for clarity, except those on acid groups.

The designed solvent-free method contained two simple steps: (1) grinding the mixtures of Cr salts and organosulfonate ligands and (2) heating the mixtures in a sealed system. The solvent-free reaction between NAP(COOH)_2_(SO_3_H)_2_ and Cr^3+^ ions afforded a new Cr(iii)-sulfonate framework, designated as TGU-9 (Fig. 2 and S1†). Meanwhile, hydrothermal synthesis using the same ligand with the Cr^3+^ ion yielded a different carboxylate-coordinated Cr-MOF, TGU-11 (Fig. S1†).

**Fig. 3:**
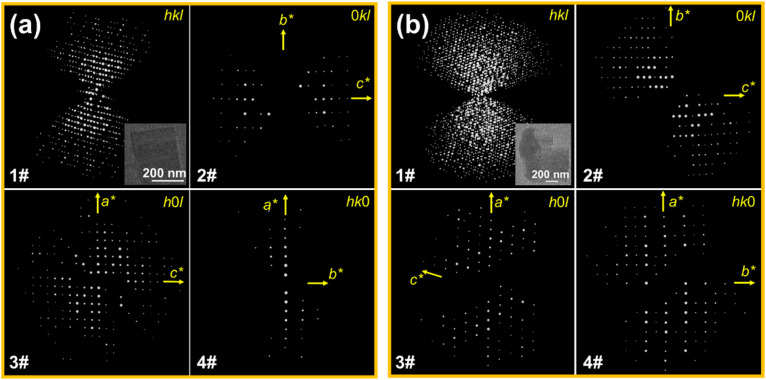
The reconstructed 3D ED datasets of TGU-9 (a) and TGU-10 (b) and the sectioned 0*kl*, *h*0*l* and *hk*0 planes of reciprocal space.

Since the solvent-free reaction products resulted in a small crystal size of several micrometers, we adopted the newly developed three-dimensional electron diffraction (3D ED) technique, specifically the continuous rotation electron diffraction (cRED) method, to solve the crystalline structure.^46–48^ The sectioned planes of TGU-9 showed the reflection conditions: 0*k*0: *k* = 2*n*, suggesting the space group *P*2_1_ (No. 4) or *P*2_1_/*m* (No. 11) (Fig. S4†). The structure model of TGU-9 was then directly determined using the program SHELXT in the space group *P*2_1_/*m* with the 3D ED datasets shown in Fig. 3a. The unit cell parameters were further refined against high resolution PXRD data by the Pawley method (Fig. S5c†).

### Coordination reversal

Through 3D ED structure solution, we found that the two sulfonate groups on each ligand in TGU-9 separately coordinate with Cr^3+^ ions in bidentate and chelating modes (Fig. 2a). A bidentate sulfonate group, a chelating sulfonate group, and a μ_2_-OH bridge work together as a repeating unit that alternately connects Cr^3+^ ions, forming a chain-like second building unit (SBU) of Cr(OH)(SO_3_)_2_ (Fig. 2b). Notably, this Cr-chain represents a previously unreported SBU, in which the adjacent CrO_6_ octahedra share the μ_2_-OH–μ_3_-O edge, distinctly different from the SBUs reported in carboxylate-based Cr-CPs (Fig. S7†).^31,32^ The naphthalene rings stack in parallel and link the adjacent Cr–SO_3_ SBUs, forming a 2D layer along the *ab* plane (Fig. S6a and b†). The –COOH groups on the ligands do not coordinate with the Cr^3+^ ions but instead form strong hydrogen bonds with those in the adjacent layer (Fig. S6c†), eventually leading to the formation of a porous 3D network (Fig. 2c). A 1D channel, surrounded by the 1D Cr-SBU and uncoordinated –COOH groups, is generated along the *b*-axis with a diameter of approximately 3.5 Å (Fig. S8†). This channel provides an ideal hydrophilic reservoir for water sorption and proton conduction. Moreover, the successive 1D Cr-SBUs, coupled with strong π⋯π interactions between the adjacent ligands and hydrogen bonds (Table S3†), effectively enhance the stability of TGU-9.

The PXRD pattern of as-synthesized TGU-11 exhibits excellent agreement with that of the Cr-carboxylate-coordinated 3D MIL-101_NDC framework (Fig. S1a†), which comprises Cr_3_O trimers and 2,6-naphthalenedicarboxylate linkers.^49^ Based on the unit cell parameters derived from Pawley refinement (Fig. S5e†) and the structural topology of MIL-101_NDC, a model of TGU-11 was constructed by replacing the 2,6-naphthalenedicarboxylate linkers in MIL-101_NDC with NAP(SO_3_H)_2_(COO)_2_^−^ moieties. As illustrated in Fig. 2d–f and S6e,† the Cr-carboxylate-coordinated TGU-11 features two types of extra-large mesopores with diameters of 39 Å and 46 Å, in addition to the pentagonal (13.5 Å) and hexagonal (18 × 20 Å) windows. Notably, a considerable number of uncoordinated –SO_3_H/–SO_3_^−^ groups are present within the pores.

By comparing the Cr-carboxylate TGU-11 with its sulfonate counterpart TGU-9, the reversal in coordination trends from –COO^−^ > –SO_3_^−^ to –SO_3_^−^ > –COO^−^ becomes evident. This finding converts the common understanding that the sulfonate group typically exhibits a relatively weaker coordinating ability with hard metal ions compared to the carboxylate group. It also highlights the effectiveness of the solvent-free method in synthesizing new CPs—a significant achievement that is difficult to realize through traditional solution chemistry.

### General synthesis and Cr–SO_3_ coordination features

Encouraged by the above results, we aimed to synthesize more Cr-sulfonate CPs using the solvent-free method to assess its general applicability. Naphthalene-1,5-disulfonic acid (H_2_NDS) was thus employed to construct TGU-7 and TGU-8, while benzene-1,4-disulfonic acid (H_2_BDS) was used to synthesize TGU-10. Systematic attempts to synthesize Cr(iii)-sulfonate CPs *via* hydrothermal treatment using H_2_NDS and H_2_BDS with three common chromium sources (CrCl_3_·6H_2_O, Cr(NO_3_)_3_·9H_2_O, and CrO_3_) were unsuccessful, as no crystalline products were obtained (Table S1†).

**Fig. 4:**
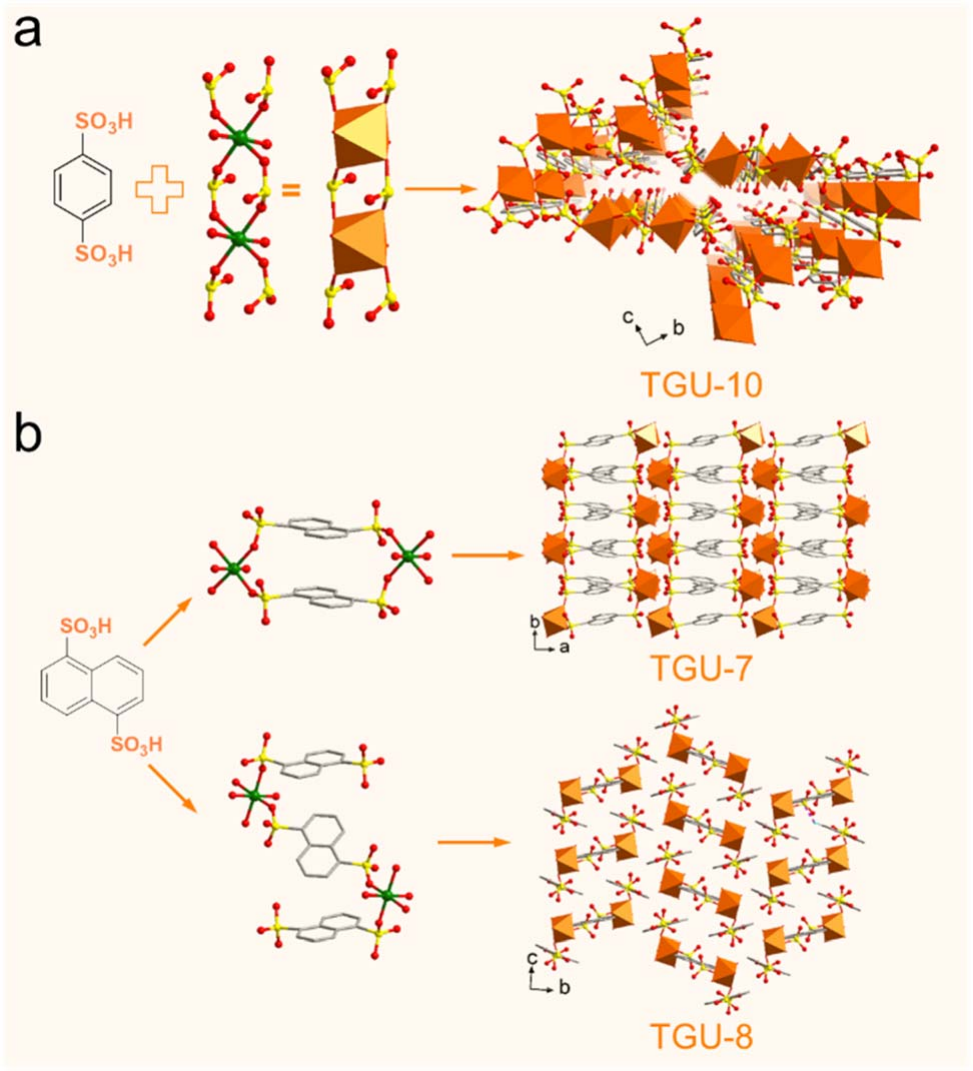
Tuned Cr–SO_3_ coordination under solvent-free conditions. (a) TGU-10 consisted of BDS^2−^ linkers and Cr_2_(SO_3_)_6_(H_2_O)_4_ SBUs. (b) TGU-7 and TGU-8 supramolecular frameworks constructed by NDS^2−^ linkers and different 0D Cr–SO_3_ clusters.

The structures of TGU-7, TGU-8 and TGU-10 were also solved using the 3D ED method and refined by the Pawley method (Fig. 3b, S3 and S5†). The extracted sectioned planes of TGU-7, TGU-8 and TGU-10 showed similar reflection conditions: *h*0*l*: *l* = 2*n*, 0*k*0: *k* = 2*n*, and 00*l*: *l* = 2*n*, suggesting the space group of *P*2_1_/*c* (No. 14). These structures were then directly determined using the program SHELXT (Table S2†). As shown in Fig. 4b, both TGU-7 and TGU-8 consist of 0D clusters, but they differ in the molar ratio of Cr^3+^ to ligand. This difference indicates that the Cr–SO_3_ coordination can be tuned just by adjusting the water content in the hydrated chromium trichloride (Fig. S9–S11†), although 0D clusters are often insufficient for constructing porous and stable CPs.^50^

Furthermore, by using the highly symmetric linear H_2_BDS ligand, the Cr–SO_3_ coordination could also be tuned to obtain TGU-10. Another new Cr-SBU of formula Cr_2_(SO_3_)_6_(H_2_O)_4_ in this CP was observed (Fig. 4a), in which two side-by-side Cr atoms are alternatively linked by sulfonate groups. Each Cr atom coordinates four sulfonate groups and two water molecules. The di-nuclear Cr-SBUs and three benzene rings of BDS^2−^ linkers alternate with CH/π interaction, forming the unique 1D Cr-belts (Fig. S12a†). Under multiple hydrogen-bonding interactions (Table S4†), the Cr-belts stack in a parallel arrangement, creating a jagged three-dimensional structure with ultra-small voids. These voids, along with the adjacent Cr-SBUs, form a hydrophilic layer along the *bc* plane (Fig. S12b and d†), potentially suitable for water sorption and proton transport. In a word, the construction of TGU-7, TGU-8, TGU-10 as well as the aforementioned TGU-9 strongly proves the general applicability of the solvent-free method in facilitating the Cr-sulfonate ligation and that Cr–SO_3_ coordination could be tuned.

In addition, the diverse Cr–SO_3_ coordination features were validated by Fourier-transform infrared (FT-IR) spectra. Specifically, in TGU-9, the new band with strong intensity at 983 cm^−1^ is attributed to the stretching vibration of the S–O bond of the bridging sulfonate groups, due to the strong electron-withdrawing effect of the two coordinated Cr^3+^ ions (Fig. S13a†).^51^ The blue shift of the S–O band from 1030 to 1020 cm^−1^ confirms the bidentate coordination of the sulfonate groups (Fig. S13b†). Similar band shifts were observed at 996 cm^−1^ for both TGU-7 and TGU-8, and at 977 cm^−1^ for TGU-10 (Fig. S14 and S15†). However, no new band or band shift of the sulfonate group was observed in TGU-11. The carboxylate coordination in TGU-11 was confirmed by the disappearance of the C=O band in the –COOH groups at 1693 cm^−1^, whereas in TGU-9, this band closely matched that of the NAP(COOH)_2_(SO_3_H)_2_ ligand. The SEM-EDS images confirmed the crystallographic characteristics, showing uniform morphology and elemental distribution for TGU-9 to TGU-11 (Fig. S16–S20†), including the evident layered structure of TGU-9, which is in line with its 2D structure. After digestion in KOH/D_2_O solutions, all ligands in TGU-7 to TGU-10 remained intact (Fig. S21–S23†), consistent with the crystalline structure solved by 3D ED.

Moreover, XPS analyses of TGU-10 and TGU-11 were conducted to further investigate the electronic differences between Cr–SO_3_ and Cr–COO coordination modes (Fig. S24†). The Cr 2p_1/2_ and 2p_3/2_ binding energies in the Cr–SO_3_-coordinated compounds TGU-9 and TGU-10 are 587.88 and 578.28 eV, respectively. In contrast, the corresponding binding energies in the Cr–COO-coordinated TGU-11 are 586.88 and 577.28 eV, respectively. Notably, the Cr 2p binding energies associated with Cr–SO_3_ coordination are approximately 1.0 eV higher than those observed for Cr–COO coordination, which may be attributed to the stronger electron-withdrawing effect of the sulfonate group relative to the carboxylate group.

### Solvent-free reaction mechanism

**Fig. 5:**
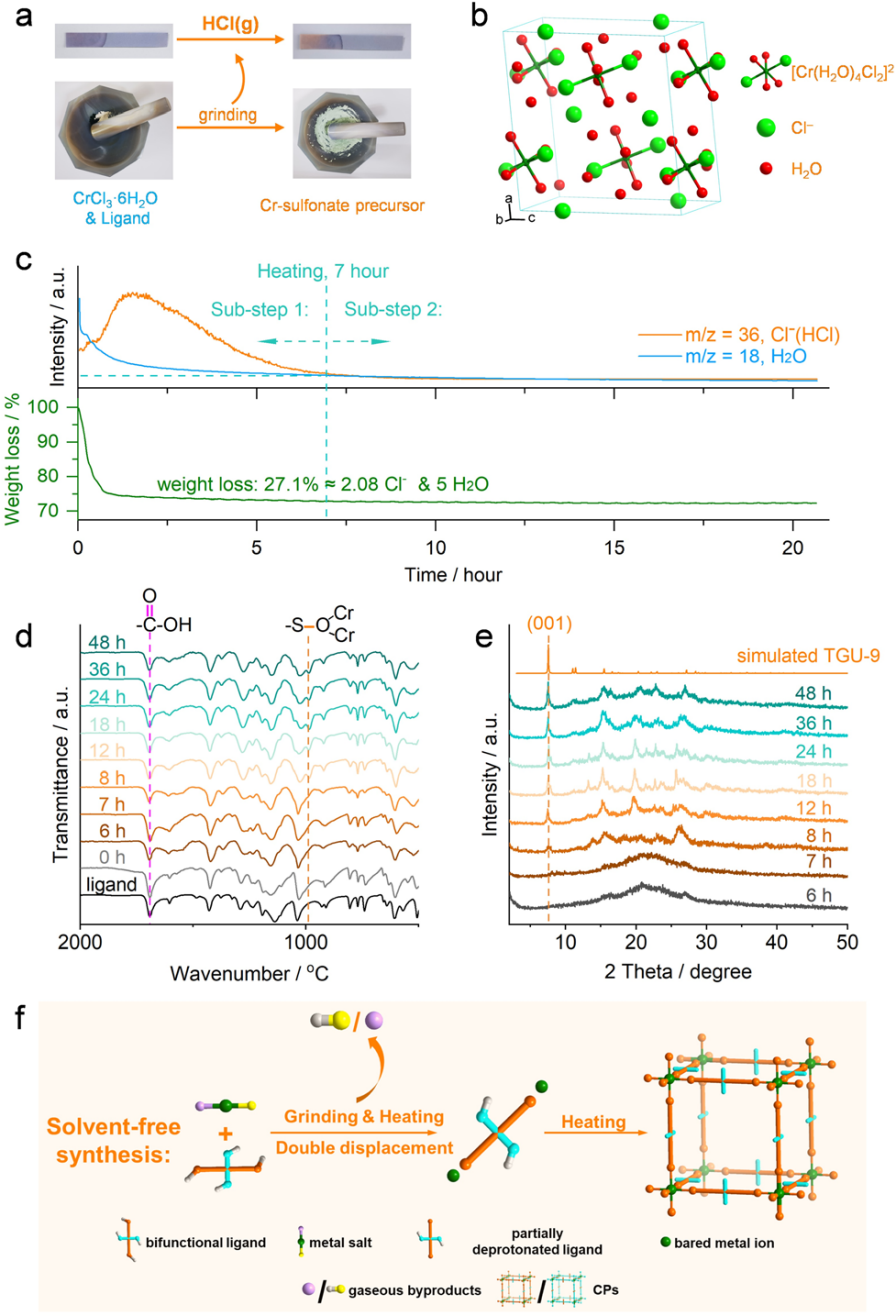
Solvent-free synthesis mechanism analysis of Cr(iii)-sulfonate frameworks. (a) Visualized detection of HCl gas during the grinding of CrCl_3_·6H_2_O and the NAP(COOH)_2_(SO_3_H)_2_ ligand. (b) Unit cell of CrCl_3_·6H_2_O. (c) Time-dependent TG-MS spectra during the heating step. (d and e) Time-dependent PXRD patterns and FT-IR spectra of the crude TGU-9, respectively. (f) Proposed solvent-free synthesis mechanism.

To investigate the reaction mechanism behind the unique Cr(iii)-sulfonate coordination under solvent-free conditions, the reaction process of TGU-9 was comprehensively monitored using various techniques (Fig. 5). In addition, Cr(NO_3_)_3_·9H_2_O and different ligands were also adopted to separately verify the proposed solvent-free reaction mechanism.

Upon grinding equimolar CrCl_3_·6H_2_O and NAP(COOH)_2_(SO_3_H)_2_, an acidic gas was released, as evidenced by the color change of moist blue litmus paper to red (Fig. 5b, ESI video†). Based on the composition of the reactants, the gas can be reasonably inferred to be HCl, which was subsequently confirmed by mass spectrometry (MS) (HCl gas is detected at 0 hour, Fig. 5c).^52^ These observations indicate that a double displacement reaction occurred during the grinding stage at room temperature, *i.e.*, CrCl_3_·6H_2_O and NAP(COOH)_2_(SO_3_H)_2_ exchanged their ions and subsequently released HCl gas. Due to the higher acidity of the –SO_3_H group than the –COOH group (for instance, benzenesulfonic acid has a p*K*_a_ value of 0.7, while benzoic acid has a p*K*_a_ value of 4.2), the H^+^ ions of the –SO_3_H groups readily combined with the Cl^−^ ions, while the –COOH groups remained unaffected. This double displacement reaction resulted in the formation of a new Cr(iii)-sulfonate precursor, as demonstrated by the shift of the Cr 2p binding energy (Fig. S25†).

Grinding step:

I CrCl_3_·6H_2_O (s) + NAP(COOH)_2_(SO_3_H)_2_ (s) → CrCl_2.08_NAP(COOH)_2_(SO_3_H)_1.08_(SO_3_)_0.92_·6H_2_O (s) + 0.92HCl (g)

Heating step:

Sub-step 1 (0–7 hours):

II CrCl_2.08_NAP(COOH)_2_(SO_3_H)_1.08_(SO_3_)_0.92_·6H_2_O (s) → Cr(OH)NAP(COOH)_2_(SO_3_)_2_ (s, amorphous) + 5H_2_O (g) + 2.08HCl (g)

Sub-step 2 (after 7 hours):

III Cr(OH)NAP(COOH)_2_(SO_3_)_2_ (s, amorphous) → Cr(OH)NAP(COOH)_2_(SO_3_)_2_ (s, crystalline TGU-9)

Overall reaction:

IV CrCl_3_·6H_2_O (s) + NAP(COOH)_2_(SO_3_H)_2_ (s) → Cr(OH)NAP(COOH)_2_(SO_3_)_2_ (s, crystalline TGU-9) + 5H_2_O (g) + 3HCl (g)

Notably, in contrast to the continuous reaction observed between the –SO_3_H group and the Cr^3+^ ion, the C=O stretching band remained unchanged throughout the reaction process (0–48 h, Fig. 5d), suggesting that the –COOH group did not participate in coordination with Cr^3+^ ions.

To quantitatively study the released Cl^−^ ions and determine the chemical composition of the solid reaction product, TG-MS analysis was further employed during the heating step. Notably, the release of both HCl gas and water vapor ceased at 7 hours, indicating that the heating process can be divided into two sub-steps: sub-step 1 (0–7 hours) and sub-step 2 (after 7 hours). The double displacement reaction still occurred during sub-step 1. In this period, a total weight loss of 27.1% observed on the TG curve can be attributed to the release of HCl and the loss of coordinated and crystalline water from the CrCl_3_·6H_2_O reactant. Since all 3 equivalents of Cl^−^ ions per Cr^3+^ ion are released as HCl gas, while each ligand provides only 2 equivalents of H^+^ ions from the –SO_3_H groups, it is deduced that one water molecule probably undergoes dissociation into OH^−^ (which finally coordinates with Cr^3+^ as observed in TGU-9) and H^+^ ions. Thus, by subtracting the contribution from the water molecules, the calculated amount of Cl^−^ ions released during heating corresponds to approximately 2.08 equivalents per Cr^3+^ ion. Accordingly, we infer that *ca.* 0.92 equivalents of Cl^−^ ion per Cr^3+^ ion are released as HCl gas during the grinding step. Based on these quantitative analysis, the composition of the grinding-derived solid product is likely CrCl_2.08_NAP(COOH)_2_(SO_3_H)_1.08_(SO_3_)_0.92_·6H_2_O. The solid intermediate product formed during sub-step 1 can be expressed as (Cr(OH)NAP(COOH)_2_(SO_3_)_2_). Thus, the corresponding reaction equations can likely be described by eqn (I) and (II), respectively.

Continuous heating during sub-step (2) facilitated the transformation of the amorphous Cr(OH)NAP(COOH)_2_(SO_3_)_2_ intermediate into the final crystalline TGU-9, as evidenced by the formation of Cr–SO_3_ coordination bonds (Fig. 5d) and the gradual emergence of the diffraction peak (Fig. 5e). Accordingly, the transformation occurring in sub-step (2) can likely be described by eqn (III). Therefore, the overall reaction is summarized in eqn (IV).

Moreover, replacing NAP(COOH)_2_(SO_3_H)_2_ with NAP(COOH)_2_(SO_3_Na)_2_ resulted in an unknown product with low crystallinity rather than TGU-9 (Fig. S26†), further demonstrating the double displacement reaction between CrCl_3_·6H_2_O and –SO_3_H groups.

In addition to CrCl_3_·6H_2_O, Cr(NO_3_)_3_·9H_2_O as a Cr source was also employed to investigate the double displacement reaction. As shown in Fig. S27a,† TGU-9 was successfully obtained by using Cr(NO_3_)_3_·9H_2_O. HNO_3_ gas was also detected during the grinding step (Fig. S27b†), further implying the occurrence of the double displacement reaction between the Cr(NO_3_)_3_·9H_2_O and the –SO_3_H group. Moreover, after analyzing the crystal structure of both CrCl_3_·6H_2_O and Cr(NO_3_)_3_·9H_2_O, uncoordinated Cl^−^ ions and NO_3_^−^ ions can be found in their corresponding unit cell (Fig. 5b, S28 and S29†).^53^ In particular, the presence of one equivalent of uncoordinated Cl^−^ ion per Cr^3+^ in CrCl_3_·6H_2_O broadly coincides with the observed release of 0.92 equivalents of Cl^−^ during the grinding step. These results may suggest that uncoordinated anions in the metal salts possess high reactivity, preferentially combining with H^+^ ions from –SO_3_H groups to release HCl gas—even during the grinding step—thereby facilitating Cr–SO_3_ coordination.

Besides, by increasing the molar ratio of CrCl_3_·6H_2_O to NAP(SO_3_H)_2_(COOH)_2_ to 2 : 1, a new CP, TGU-*X*, was synthesized under the same reaction conditions employed for synthesizing TGU-9 (Fig. S30a†). Although the structural determination *via* 3D ED was not feasible due to the material's intrinsic flexibility and low crystallinity in the dried state (Fig. S30b†), FT-IR spectroscopy provided valuable insight into the coordination environment. The FT-IR spectrum of TGU-*X* shows the disappearance of the carbonyl (C=O) stretching vibration at 1693 cm^−1^ (Fig. S13a†), along with a shift and notable broadening of the S–O stretching vibration band from 1020 to 1030 cm^−1^ (Fig. S13b†). These spectral changes indicate the simultaneous coordination of both carboxylate and sulfonate groups to Cr^3+^ centers in TGU-*X*. The dual coordination exhibited in TGU-*X*, in contrast to the carboxylate coordination observed in TGU-11 and the sulfonate coordination in TGU-9, provides additional evidence that a low dosage of CrCl_3_·6H_2_O can trigger the reversal in coordination preference from –COO^−^ > –SO_3_^−^ to –SO_3_^−^ > –COO^−^.

To validate the generality of the coordination reversal, 2-sulfoterephthalic acid (SBDC), 2,5-disulfoterephthalic acid (DSBDC) and 3,3′-disulfo-[1,1′-biphenyl]-4,4′-dicarboxylic acid (DSBPDC) were prepared and used to react with CrCl_3_·6H_2_O under solvent-free conditions (Schemes S1–S3 and Fig. S31–S34†). The corresponding products, Cr-SBDC, Cr-DSBDC, and Cr-DSBPDC, were obtained. FT-IR spectra of all three compounds reveal new S–O stretching bands, while the characteristic C=O stretching vibrations remain unchanged. Moreover, the XPS results reveal that the binding energies of Cr 2p_3/2_ and 2p_1/2_ in these compounds are similar to those observed in the sulfonate-coordinated TGU-9 and TGU-10 and *ca.* 1.0 eV higher than those in Cr–CO_2_ coordinated TGU-11 (Fig. S13†), also demonstrating the Cr–SO_3_ coordination, although these crystal structures could not be resolved due to low crystallinity (Fig. S32†).

We also discovered that the entire process of this reaction took place in the solid state (Fig. S35†), indicating that the solvation of Cr^3+^ ions, which normally occurs in solution, was avoided due to the absence of bulk solvent. The condensation of water droplets inside the sealed glass tube after the reaction, along with the detected water signal in the MS spectra, strongly indicates that the coordinated and crystalline water molecules in [Cr(H_2_O)_4_Cl_2_]Cl·2H_2_O were readily lost under solvent-free and high-temperature conditions. This loss obviously facilitates the exposure of the unoccupied molecular orbitals on the Cr^3+^ ions, allowing for efficient coordination with sulfonate groups.

Based on the experimental analysis of the reaction process and the composition of the reactants, we propose a solvent-free synthesis mechanism, as illustrated in Fig. 5f. During the grinding and subsequent heating of the metal salt and ligand, a typical double displacement reaction occurs: the reactants get thoroughly mixed and exchange the ions, resulting in the formation of gaseous byproducts and a new solid metal–ligand precursor. With further heating, the precursor continues undergoing double displacement reactions, followed by crystallization, ultimately yielding the final CPs. The absence of bulk solvent tactfully avoided the solvation of metal ions and the corresponding inert substitution of the solvent molecules commonly seen in solution chemistry, thus controlling the formation kinetics of CPs. Most importantly, this reaction mechanism reverses the widely accepted coordination trend, shifting from the conventional –COO^−^ > –SO_3_^−^ to the unprecedented –SO_3_^−^ > –COO^−^, as exemplified by TGU-9. The higher acidity of the –SO_3_H group compared to the –COOH group enables preferential deprotonation by Cl^−^/NO_3_^−^ ions, ultimately leading to Cr–SO_3_ coordination. In addition, the generated HCl/HNO_3_ gas may also inhibit the deprotonation and coordination of –COOH groups, thereby preserving them within the structure.

In contrast, under aqueous conditions, the sulfonate groups on the NDS^2−^ and BDS^2−^ ligands exhibit low coordination ability, making it difficult to substitute the coordinated water on the hydrated chromium ions and form crystalline sulfonate frameworks, as shown in Table S1.† For the NAP(COOH)_2_(SO_3_H)_2_ ligand, both the –SO_3_H and –COOH groups deprotonate in an aqueous solution. Owing to the higher coordination ability of the –COO^−^ group compared to the –SO_3_^−^ group, the Cr(iii)-carboxylate TGU-11 is assembled, with the –SO_3_H/–SO_3_^−^ groups remaining uncoordinated.

### High stability and proton conductivity

**Fig. 6:**
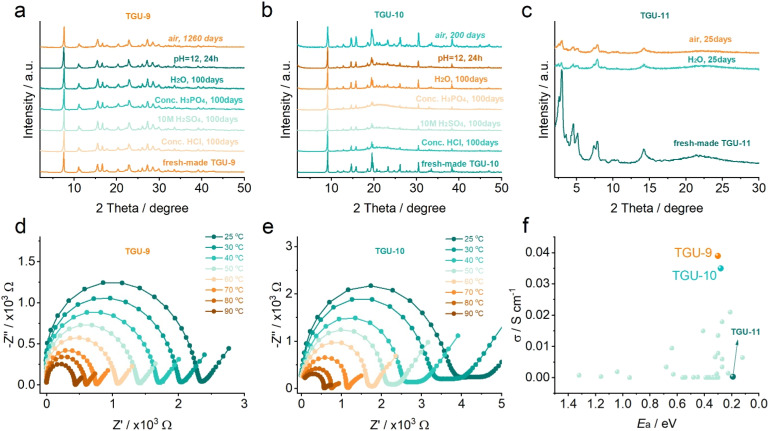
High structural and proton-conducting stability of TGU-9 and TGU-10. (a–c) Chemical stability tests of TGU-9, TGU-10, and TGU-11, respectively. (d and e) Temperature-dependent impedance plots of TGU-9 and TGU-10 at 100% RH after long-time storage, respectively. (f) The proton conduction comparison of TGU-9, TGU-10 and TGU-11 as well as the reported sulfonate-coordinated CPs. The impedance plots of TGU-9 and TGU-10 (d and e) were obtained using the corresponding cuboid plate samples. The proton conduction data in (f) are also listed in Table S5.†

Based on the fantastic Cr–SO_3_ coordination modes and framework structures, the stabilities of these new CPs, which are crucial for applications,^54^ were investigated. TGU-9 displayed exceptionally high stabilities across a wide pH range (Fig. 6a), even when immersed in concentrated hydrochloric acid, phosphoric acid, and 10 M sulfuric acid for 100 days. More importantly, TGU-9 remained intact without losing water adsorption capacity after being stored in air for 1260 days (Fig. S37a and b†). This is the longest stability record of CPs reported to date,^55–57^ superior to other sulfonate CPs. Unexpectedly, the carboxylate-based TGU-11 showed reduced crystallinity in water and air after 25 days (Fig. 6c), demonstrating poorer stability than its sulfonate counterpart TGU-9.

Similar to TGU-9, the sulfonate-coordinated TGU-10 also exhibits excellent stability in strong acid and retained its high crystallinity after being stored for 200 days (Fig. 6b). TGU-7 collapsed in bulk water, while TGU-8 dissolved rapidly, losing approximately 55 wt% (Fig. S38 and S39†), indicating poor stability probably due to their single Cr-node structure. TG curves and *in situ* PXRD patterns revealed that TGU-9 and TGU-10 maintained structural integrity at temperatures up to 400 and 300 °C, respectively, superior to TGU-7 (220 °C) and TGU-8 (220 °C) (Fig. S40–S45†). The high stability of TGU-9 and TGU-10 can be attributed to their unique Cr-SBUs and multiple interactions such as strong hydrogen bonds, π–π interaction, or CH/π interaction in their frameworks.

To further explore the application potential, we investigated the proton conductivity (Fig. S46–S52†). TGU-9, TGU-10, and TGU-11 show increased proton conductivities as the relative humidity (RH) increased. At 100% RH, TGU-9 exhibited smooth and perfect Nyquist plots, with gradually reduced Debye semicircles as the temperature increased from 25 to 90 °C (Fig. 6d). Remarkably, the proton conductivity of TGU-9 steadily increased to 3.5 × 10^−2^ S cm^−1^ at 90 °C and 100% RH. To the best of our knowledge, TGU-9 demonstrates the highest proton conductivity among the proton conducting sulfonate CPs or MOFs (Fig. 6f and Table S5†) and comparable to the commercial Nafion membrane. This conductivity is also two orders of magnitude higher than that of TGU-11 (3.14 × 10^−4^ S cm^−1^, Fig. S53†) under the same condition, despite TGU-11 having a much higher water adsorption capacity (Fig. S37c†) and abundant acidic –SO_3_H groups. Similarly, TGU-10 exhibited a high proton conductivity of 3.39 × 10^−2^ S cm^−1^ at 90 °C and 100% RH (Fig. 6e), slightly lower than TGU-9 but higher than other reported sulfonate CPs and the Cr-carboxylate TGU-11.

Moreover, the conductivities of TGU-9 and TGU-10 remain consistent with those of the freshly prepared samples (Fig. S54†), even after being stored for 1260 days and 200 days, respectively. Both TGU-9 and TGU-10 also retain high proton conductivities at 80 °C and 100% RH for 120 hours while simultaneously preserving their structural integrity (Fig. S55–S58†). To date, numerous proton-conducting CPs/MOFs with terminal sulfonate/sulfonic groups that are not coordinated to metal centers have shown excellent proton conductivities, in some cases reaching up to 10^−1^ S cm^−1^.^43,58–64^ Although the proton conductivities of TGU-9 and TGU-10 are comparable to, or slightly lower than, those of these top-performing materials, their superior stability presents a significant advantage for potential practical applications in proton-conducting devices. This balance between performance and robustness highlights the value of designing Cr-sulfonate CPs/MOFs.

The activation energies (*E*_a_) for proton transport in TGU-9, TGU-10, and TGU-11 were calculated to be 0.28, 0.30, and 0.18 eV, respectively, at 100% relative humidity (RH) (Fig. S59†). These values suggest that proton conduction in all three compounds follows the Grotthuss mechanism (proton hopping), typically characterized by *E*_a_ < 0.4 eV.^65^ The low *E*_a_ observed for TGU-11 can be attributed to the presence of uncoordinated –SO_3_H groups, which readily donate protons. However, as previously discussed, the proton conductivities of TGU-9 and TGU-10 are significantly higher than that of TGU-11. This apparent contradiction can be explained by their distinct pore structures, as revealed by N_2_ and water vapor adsorption analyses (Fig. S36 and S38†). TGU-9 and TGU-10 possess ultra-small pores that facilitate the formation of dense hydrogen-bonding networks upon water uptake, in contrast to the large pores of TGU-11, where hydrogen bonding is relatively sparse. Molecular simulations further support this interpretation: in TGU-9, water molecules are confined within one-dimensional channels, forming continuous hydrogen bonds with one another and with sulfonate groups (Fig. S60†). In TGU-10, water resides in the confined spaces between adjacent di-nuclear Cr-SBUs within the hydrophilic layer (Fig. S61†), where it also engages in extensive hydrogen bonding with coordinated water molecules and sulfonate groups. These hydrogen-bonding networks serve as efficient pathways for proton hopping, ultimately accounting for the high proton conductivities observed in TGU-9 and TGU-10.

## Conclusions

In this study, we successfully designed a solvent-free method for creating Cr(iii)-sulfonate frameworks, overcoming the inherent challenges associated with the low coordination ability of the sulfonate group and the solvation of Cr^3+^ in traditional solution chemistry. The solvent-free method demonstrated broad applicability, enabling the synthesis of two Cr-sulfonate CPs (TGU-9 and TGU-10), and two Cr-sulfonate-coordination supramolecules (TGU-7 and TGU-8). Besides, a different Cr-carboxylate MOF (TGU-11) was also developed in an aqueous solution of the same ligand in TGU-9. Interestingly, the coordination preference was reversed from the familiar –COO^−^ > –SO_3_^−^ to the unprecedented –SO_3_^−^ > –COO^−^, thus preserving the uncoordinated –COOH groups in sulfonate-coordinated TGU-9. The reaction mechanism analysis shows that the Cr–SO_3_ coordination was initiated by a double displacement reaction between the Cr-salt and the –SO_3_H group, leading to the formation of the Cr-sulfonate salt that ultimately transformed into Cr-sulfonate CPs. Notably, TGU-9 and TGU-10 exhibited exceptional long-term stability in air and across a wide pH range, as well as the highest proton conductivities among the sulfonate CPs. The successful construction of Cr-sulfonate CPs with the solvent-free synthetic mechanism in this work opens the door to new CPs/MOFs—extending beyond sulfonate-based systems—that can be synthesized in a more sustainable and efficient manner without the use of solvents.

## Author contributions

F. Y. conceived the idea; J. L., J. S. and C. Z. supervised the project; X. W., J. J. and J. S. performed the structural analysis; F. Y. and K. W. conducted synthesis, chemical analysis, and proton conductivity tests; H. D. and G. C. collected the PXRD patterns for refinement; Z. Z. carried out the molecular simulation; F. Y., X. K. and T. H. prepared the manuscript. All authors discussed the results.

## Conflicts of interest

There are no conflicts to declare.

## Supplementary Material

SC-OLF-D5SC03014E-s001

SC-OLF-D5SC03014E-s002

SC-OLF-D5SC03014E-s003

## Data Availability

The data supporting this work are available in the ESI.†
