# Supplementary material for: Solvent-free construction of Cr(iii)-sulfonate coordination polymers
Source: Chem Sci. 2025 May 31;16(26):11823–32. doi: 10.1039/d5sc03014e (PMC12146893; doi:10.1039/d5sc03014e)
Supplement: SC-016-D5SC03014E-s001 [file SC-016-D5SC03014E-s001.pdf]

Supplementary Information (SI) for Chemical Science.
This journal is © The Royal Society of Chemistry 2025

Supplemental Information

for

Solvent-free Construction of Cr(III)-sulfonate Coordination Polymers

Fan Yang,[a] Xiang-Jing Kong,[b] Tao He,[b] Zhengqing Zhang,[a] Ke Wang,[a] Honglin Du,[c] Guohong Cai,[c] Jing Ju,[c] Xiaoge Wang,[c,*] Jian-Rong Li,[b,*] Junliang Sun[c,*] and Chongli Zhong[a,*]

a State Key Laboratory of Separation Membranes and Membrane Processes, School of Chemistry and Chemical Engineering, Tiangong University, Tianjin 300387, P. R. China

b Beijing Key Laboratory for Green Catalysis and Separation and Department of Chemical Engineering, College of Materials Science and Engineering, Beijing University of Technology, Beijing, China

c College of Chemistry and Molecular Engineering, Beijing National Laboratory for Molecular Sciences, Peking University, Beijing, China

Additional experimental details

Synthesis and Characterization

Synthesis of $NAP(COOH)_2(SO_3H)_2$ and H_2BDS ligands. The $NAP(COOH)_2(SO_3H)_2$ and H_2BDS ligands were synthesized following previous reports.[1,2]

Warning: HCl gas can be released during synthesizing TGU-7~TGU-10. To minimize exposure, the initial grinding process and autoclave opening should be performed inside a well-ventilated fume hood while wearing appropriate personal protective equipment.

Synthesis of TGU-7. $CrCl_3·6H_2O$ (233 mg, 0.875 mmol) and H_2NDS (144 mg, 0.5 mmol) were manually ground in an agate mortar by hand for 15 minutes. The resulting mixture was sealed in a Teflon-lined stainless-steel autoclave and heated at 220 °C for 48 h. After cooling to room temperature, the crude product was washed five times with deionized water and twice with ethanol. A pale green powder was obtained after centrifugation and drying in a vacuum at 80 °C for 12 hours. Elemental analysis (EA) for TGU-7: $C_{20}H_{26}Cr_2O_{20}S_4$ (FW: 818.66): calculated (%): Cr, 12.70; C, 29.34; H, 3.20 and S, 15.66. Found (%): Cr, 12.65 (ICP); C, 30.00; H, 3.51; and S, 16.02.

Synthesis of TGU-8. TGU-8 was synthesized similarly to TGU-7, except adopting the chromium chloride tetrahydrate (201mg, 0.875 mmol) and grinding in a glovebox under a nitrogen atmosphere to prevent exposure to moisture in the air. Yield, 85 mg, 95.45% based on based on H_2NDS. EA for TGU-8: $C_{15}H_{17}CrO_{13}S_3$ (FW: 553.48): calculated (%): Cr, 9.40; C, 32.54; H, 3.09; and S, 17.38. Found (%): Cr, 9.51 (ICP); C, 32.75; H, 3.21; and S, 17.02.

Synthesis of TGU-9. TGU-9 was synthesized similarly to TGU-7, except using the $NAP(COOH)_2(SO_3H)_2$ ligand (0.875 mmol, 329 mg). Yield, 368 mg, 94.65% based on $NAP(COOH)_2(SO_3H)_2$. EA for TGU-9, $C_{12}H_7CrO_{11}S_2$ (FW: 444.30): calculated (%): Cr, 11.70; C, 32.43; H, 1.59; and S, 14.46. Found (%):

Cr, 12.00 (ICP); C, 31.53; H, 1.81; and S, 15.20.

For a 10-gram scale synthesis, the procedure for TGU-9 was proportionally scaled up 30 times using a 100 mL Teflon-lined stainless-steel autoclave, heated at 220 °C for 48 hours. The yield was 10.10 g.

Synthesis of TGU-10. TGU-10 was synthesized similarly to TGU-9, except using the H_2BDS ligand (260 mg, 1.09 mmol) and performing the grinding in a glovebox under nitrogen atmosphere due to the high hygroscopic property of H_2BDS. The reaction temperature and time were 220 °C and 12 hours, respectively. Yield, 110 mg, 59.68% based on H_2BDS. EA for TGU-10, $C_9H_{10}CrO_{11}S_3$ (FW: 442.36): calculated (%): Cr, 11.76; C, 24.43; H, 2.28; and S, 21.74. Found (%): Cr, 11.00 (ICP); C, 23.51; H, 2.00; and S, 20.20.

Synthesis of TGU-11. $Cr(NO_3)_3·9H_2O$ (600 mg, 1.5 mmol), $NAP(COOH)_2(SO_3H)_2$ (376 mg, 1 mmol), hydrofluoric acid (~40 *wt*%, 15 μL), and H_2O (2 mL) were mixed and sealed in a Teflon-lined stainless-steel autoclave and then heated at 220 °C for 10 h. After cooling, the product was collected by centrifugation and washed six times with deionized water. The sample was then dried in a vacuum at 80°C for 12 hours, yielding a green powder. Yield, 121 mg, 27.11% based on $NAP(COOH)_2(SO_3H)_2$. EA for TGU-11, $C_{36}H_{29}Cr_3O_{34}S_6$.

3D ED structure analysis. The cRED method and liquid nitrogen sample holder were combined to collect the 3D ED data of the TGU-7, TGU-8, TGU-9 and TGU-10 nanocrystals to prevent the electron beam damage. For TGU-7, TGU-9 and TGU-10 samples, the cRED operations were conducted on JEOL 2100 transmission electron microscopy (TEM) at 200 kV by the Gatan oneview camera with the InsteaDMatic script. For TGU-8 sample, the data collection was performed on Themis Z transmission electron microscopy at 200 kV by the ASI medpix camera. The collected frames were then processed by the *XDS* software to search peaks,[3] find unit cell and space-group and integrate intensities. The unit cell parameters were finally determined by Pawley method

against the high resolution PXRD patterns.[4] The 3D reciprocal space *hkl* was reconstructed by REDp software and the 0*kl*, *h*0*l* and *hk*0 planes were sectioned for symmetry analysis.[5]

Structure construction of TGU-11. The subsequent structure determination by *Shelxt* method by using atomic structure factors for electrons.[6,7] The structure model of TGU-11 was built based on the pawely refinement and the MIL-101 topology by substituting the ligand molecules.

Proton conductivity measurement. The proton conductivity measurements were conducted following the same procedure outlined in our previous work.[1] In this work, we adopted two different sample geometries—cylindrical and cuboid—to accurately evaluate the proton conductivities of TGU-9, TGU-10, and TGU-11. The pressing molds and the corresponding test schematic representation is shown in Fig. S46. Figs. 6d, 6e and S47 were obtained using cuboid samples with dimensions of 0.2 × 0.4 × 1 cm^3, while Figs. S48, S49, and S53 were obtained using cylindrical pellets with a diameter of 6 mm (the thickness is added into each figure caption). The use of different sample shapes ensured the appearance of complete semicircles in the Nyquist plots under various relative humidity (RH) and temperature conditions for accurately calculating proton conductivity.

For cuboid plates, approximately 90 mg corresponding powder was pressed used the customized cuboid mold under 1,000 kg cm^{-2} pressure for 3 minutes. Both the two surface (0.2 × 0.4 cm^2) of the cuboid plate were affixed to silver wires using silver paste and sealed in a double-walled glass chamber. For cylindrical pellets, approximately 50 mg corresponding powder was pressed by the cylindrical mold under 1,000 kg cm^{-2} pressure for 3 minutes (Figs. S45a and b). Both the round surface of the cylindrical pellet were affixed to silver wires for impedance tests (Figs. S45b and c).

The temperature was regulated by a temperature-controlled circulating water within the interlayer of the double-walled glass chamber. The RH was

tuned by a series of saturated salt aqueous solutions which were prepared by dissolving the excessive corresponding salts in deionized water ($MgCl_2$, ~33% RH; $Mg(NO_3)_2$, ~53% RH; $NaNO_2$, ~65% RH; NaCl, ~75% RH; KCl, ~85% RH; deionized water, 100% RH). The impedance plots were obtained using a Zennium pro electrochemical workstation with tuned frequency range spanning from 1 Hz to 8 MHz and an alternating potential of 100 mV. The proton conductivity (σ, S cm^{-1}) was calculated by using the following equation (*I*, 1.0 cm; *R*, the measured impedance, Ω; *S*, *ca.* 0.2×0.4 cm^2):

$$\sigma = I/(RS)$$

The activation energy (E_a) was calculated by using the proton conductivity data between 25 and 90 °C at 100% RH with the Arrhenius equation (*T*, absolute temperature, K; *A*, pre-exponential factor; k_B, Boltzmann constant):

$$ln(\sigma T) = lnA - E_a/(k_B T)$$

General characterization. Powder X-ray diffraction (PXRD) patterns were recorded on a Bruker D8 Discover X-ray diffractometer with Cu K_α radiation. N_2 uptakes were measured at 77 K with a Micromeritics ASAP 2020 device. Water uptakes were recorded in a MicrotracBEL Belsorp-max instrument. SEM/EDS was recorded using a GeminiSEM 500 scanning electron microscope. TG analysis was conducted using TGA5500 thermal analyser in air atmosphere. FT-IR spectra were acquired using a Thermo Nicolet 380 spectrometer over the wave number range of 400 to 4000 cm^{-1}. X-ray photoelectron spectrometer (XPS) (Al-Kα radiation at 1486.6 eV NEXSA, Thermo Scientific) was used to analyse electronic properties. ^{1}H-NMR spectra were recorded by utilizing a Bruker Fourier 600M spectrometer. Elemental analysis was performed by the Elementar vario EL cube.

Molecular simulation. The insertion of H_2O molecules into the CP structure was achieved through configurational-bias Monte Carlo (CBMC) method, implemented using our in-house HT-CADSS suite. All framework atoms within CPs were held fixed. The H_2O molecule was represented by TIP4P modeland

the interaction between H_2O molecules and CP was described by Lennard-Jones (LJ) potential taken from UFF.[8,9] To account for the long-range electrostatic interactions, the Ewald summation method was employed. The extended charge equilibration (EQeq) method was utilized to assign the atomic charges to CPs.[10] Cross interactions between different types of atoms were estimated by the Lorentz−Berthelot (LB) mixing rule. For each CBMC simulation consisted of 2×10^6 steps conducted under NVT ensemble at a temperature of 298.0 K. The time step was set to 1.0 fs, and the cutoff distance for the non-bonded and Coulomb interactions was established at 12.0 Å. The periodic boundary conditions were applied in all *xyz* directions.

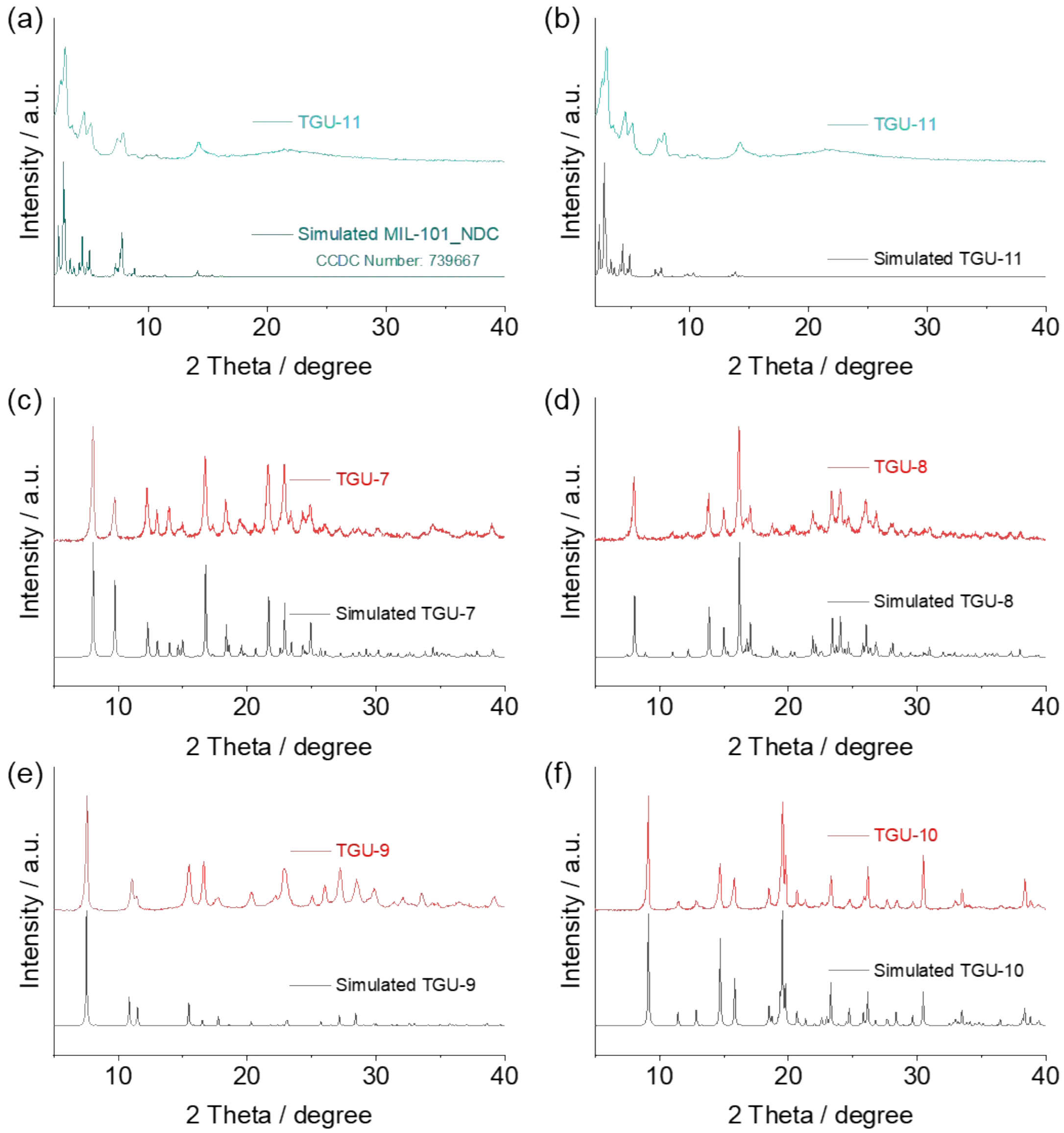

Fig. S1 Comparisons between the experimental and simulated PXRD patterns of TGU-7 to TGU-11 and the comparison between TGU-11 and MIL-101_NDC. (a), the comparison between TGU-11 and MIL-101_NDC; (b), TGU-11; (c), TGU-7; (d), TGU-8; (e), TGU-9; (f), TGU-10.

Table S1. Hydrothermal reaction results of Cr-salt with H_2NDS, H_2BDS, and $NAP(COOH)_2(SO_3H)_2$. Reaction condition: 2 ml H_2O, 220 ^oC, 24 h.

Entry	Ligand	Cr^{3+} source	n_{ligand}:n_{Cr3+}	40*wt*%HF /drops	Results
1	H_2BDS	$CrCl_3·6H_2O$	3:2	0	clear solution
2				2	clear solution
3			2:2	0	clear solution
4				2	clear solution
5			2:3	0	clear solution
6		$Cr(NO_3)_3·9H_2O$	3:2	0	green amorphism
7				1	carbonization
8				2	carbonization
9				3	clear solution
10				5	clear solution
11			2:2	0	carbonization
12				1	carbonization
13				2	carbonization
14			2:3	0	carbonization
15				5	carbonization
16				10	white precipitate
17		CrO_3	3:2	0	green amorphism
18				1	green amorphism
19				2	clear solution
20			1:1	0	green amorphism
21				1	green amorphism
22				2	clear solution
23				0	green amorphism

24				1	green amorphism
25				4	green amorphism
26				5	clear solution
27	H_2NDS	$CrCl_3 \cdot 6H_2O$	3:2	0	white precipitate
28			2:2	0	white precipitate
29			2:3	0	white precipitate
30		$Cr(NO_3)_3 \cdot 9H_2O$	3:2	0	carbonization
31			2:2	0	carbonization
32			2:3	0	carbonization
33		CrO_3	3:2	0	clear solution
34			1:1	0	green amorphism
35				1	green amorphism
36				2	clear solution
37			2:3	0	green amorphism
38				1	green amorphism
39				2	green amorphism
40				3	clear solution
41	$NAP(COOH)_2(SO_3H)_2$	$Cr(NO_3)_3 \cdot 9H_2O$	3:2	0.5	**Cr–carboxylate coordinated TGU-11**

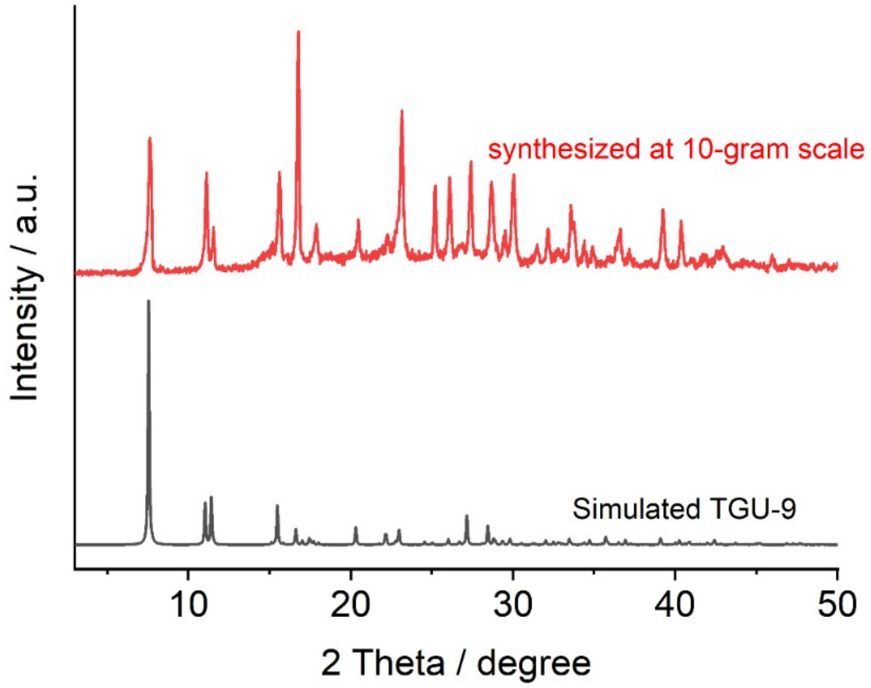

Fig. S2 PXRD pattern of TGU-9 synthesized at the 10-gram scale.

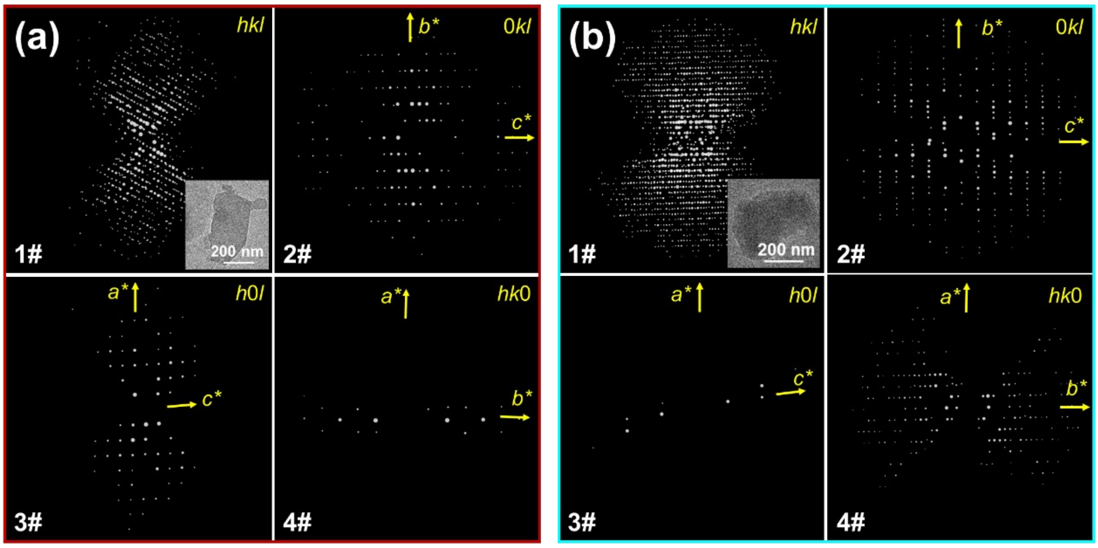

Fig. S3 The reconstructed 3D ED datasets with the sectioned 0*kl, h*0*l* and *hk*0 planes in the reciprocal space. (a), TGU-7; (b), TGU-8.

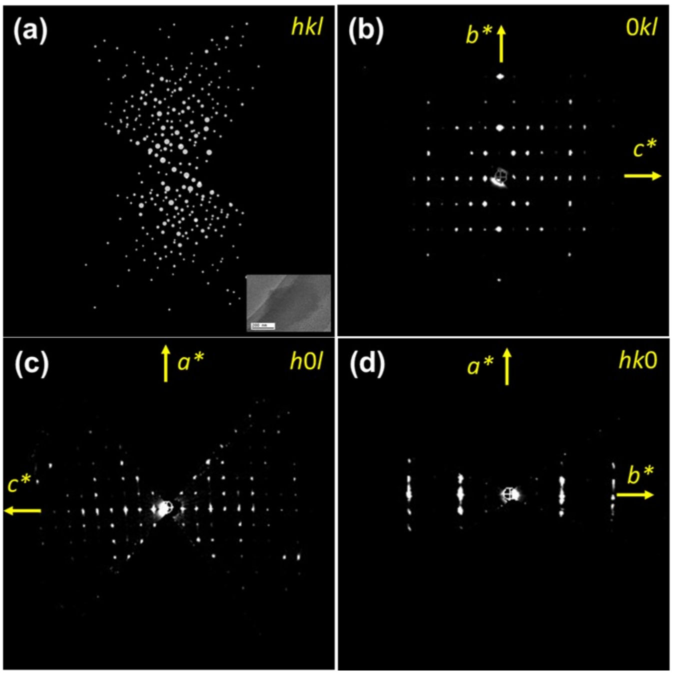

Fig. S4 Another reconstructed 3D ED dataset of TGU-9 with the sectioned 0*kl* (b)*, h*0*l* (c) and *hk*0 (d) planes in the reciprocal space.

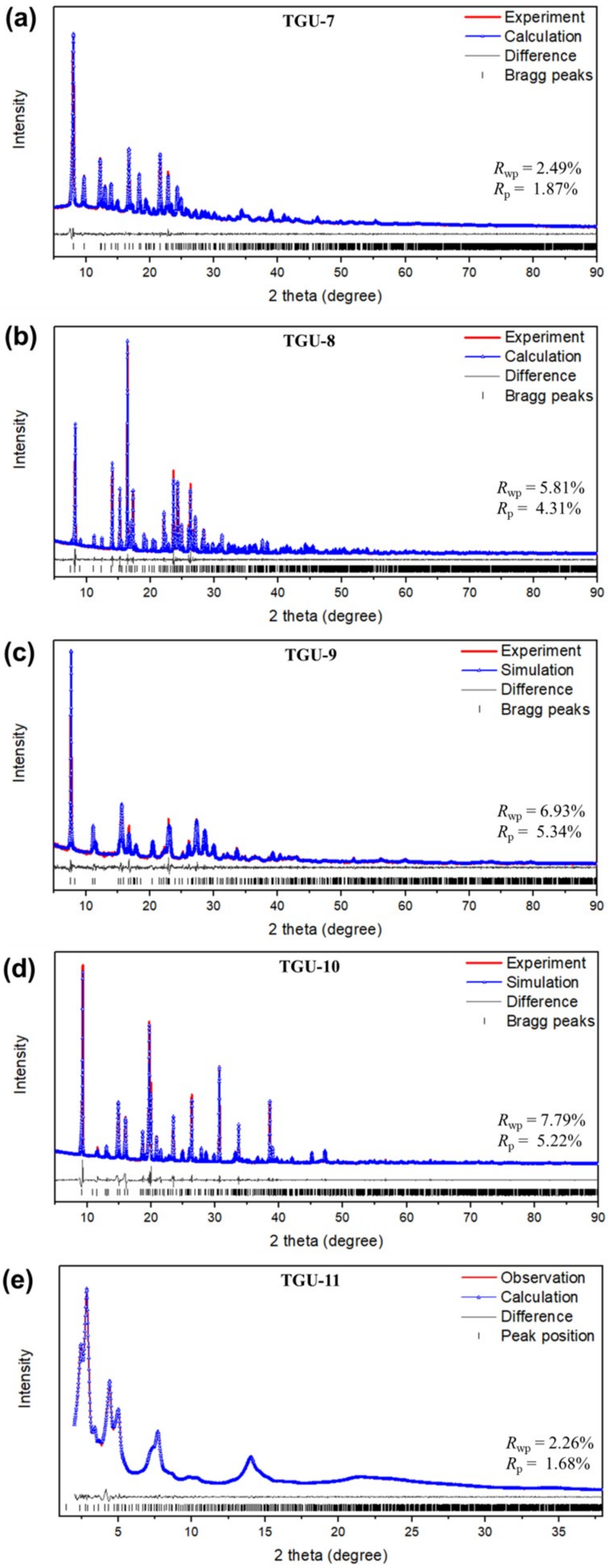

Fig. S5 Profiles fit against the powder XRD data using the Pawley method. (a), TGU-7; (b), TGU-8; (c), TGU-9; (d), TGU-10; (e), TGU-11.

Table S2. The cRED experimental parameters, crystallographic data, and structure refinement details of TGU-7, TGU-8, TGU-9 and TGU-10. The unit cell parameters were refined against powder XRD data.

**Data collection**	**TGU-7**	**TGU-8**	**TGU-9**	**TGU-10**
Tilt range (°)	-69.16 ~ 50.27	-56.97 ~ 67.99	-54.56 ~ 49.41	-70.17 ~ 72.09
Tilt step (°)	0.21	0.25	0.23	0.22
Wavelength (Å)	0.0251	0.0251	0.0251	0.0251
Exposure time per frame (s)	0.5	0.5	0.5	0.5
**Data process**	**TGU-7**	**TGU-8**	**TGU-9**	**TGU-10**
Program for data process	*XDS*	*XDS*	*XDS*	*XDS*
Program for structure solution	*ShelxT*	*ShelxT*	*ShelxT*	*ShelxT*
Crystal system	monoclinic	monoclinic	monoclinic	monoclinic
Unit cell *a*, *b*, *c* (Å) α, β, γ (°)	11.006(3), 7.772(2), 18.214(4), 90, 93.509(4), 90	11.0056(7), 23.4923(13), 7.6371(4), 90, 97.235(2), 90	10.666(7), 6.555(4), 11.700(8), 90, 92.118(18), 90	9.9289(12) 9.5629(10) 15.8800(17) 90, 103.656(3), 90
Volume (Å^3)	1555.1(7)	1958.8(2)	817.4(10)	1465.2(3)
Space group	$P2_1/c$	$P2_1/c$	$P2_1/m$	$P2_1/c$
Resolution (Å)	0.83	0.81	0.81	0.82
Completeness	65%	84%	62%	92%
R_{int}	16.39%	15.56%	7.81%	17.73%
No. of reflections	3481	6642	2114	6691
No. of unique reflections	1864	3061	1107	2479
**Structure refinement**	**TGU-7**	**TGU-8**	**TGU-9**	**TGU-10**
Index range	$-10 \leq h \leq 12$ $-9 \leq k \leq 8$ $-18 \leq l \leq 21$	$-12 \leq h \leq 12$ $-26 \leq k \leq 27$ $-8 \leq l \leq 9$	$-13 \leq h \leq 13$ $-6 \leq k \leq 6$ $-14 \leq l \leq 14$	$-11 \leq h \leq 11$ $-11 \leq k \leq 11$ $-18 \leq l \leq 18$
No. of	184	282	158	201

parameters				
No. of restraints	146	211	187	110
H-atom treatment	geometry	geometry	geometry	geometry
R [$I > 2\sigma(I)$]	R_1 = 0.2466<br>wR_2 = 0.5482	R_1 = 0.3224<br>wR_2 = 0.5995	R_1 = 0.2099<br>wR_2 = 0.4918	R_1 = 0.2207<br>wR_2 = 0.4818
R (all data)	R_1 = 0.3998<br>wR_2 = 0.6155	R_1 = 0.4603<br>wR_2 = 0.7090	R_1 = 0.2761<br>wR_2 = 0.5370	R_1 = 0.2993<br>wR_2 = 0.5493
Goodness-of-fit on F^2	1.621	2.079	1.735	1.537

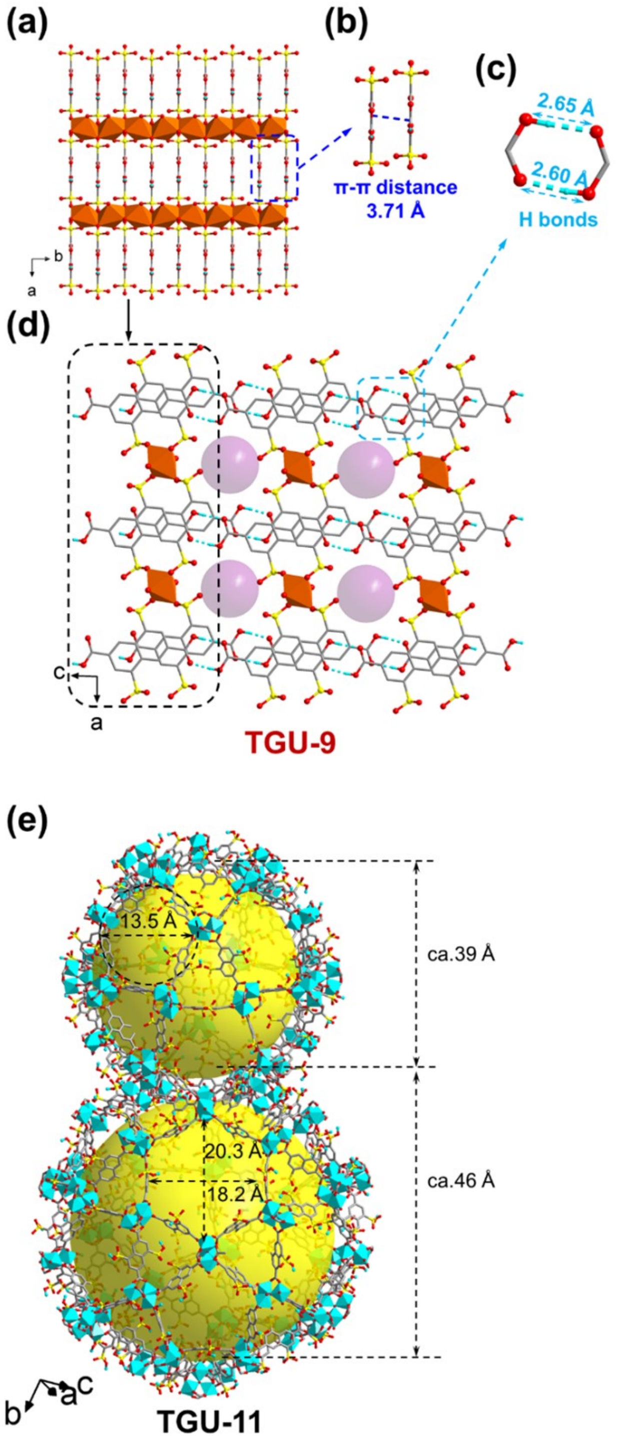

Fig. S6 Structure illustration of TGU-9 and TGU-11. (a), the single layer of TGU-9; (b) the π···π interaction between the adjacent ligands; (c), the interlayer hydrogen bonds; (d), TGU-9 structure viewed along *b*-axis; (d), the super large cages in TGU-11.

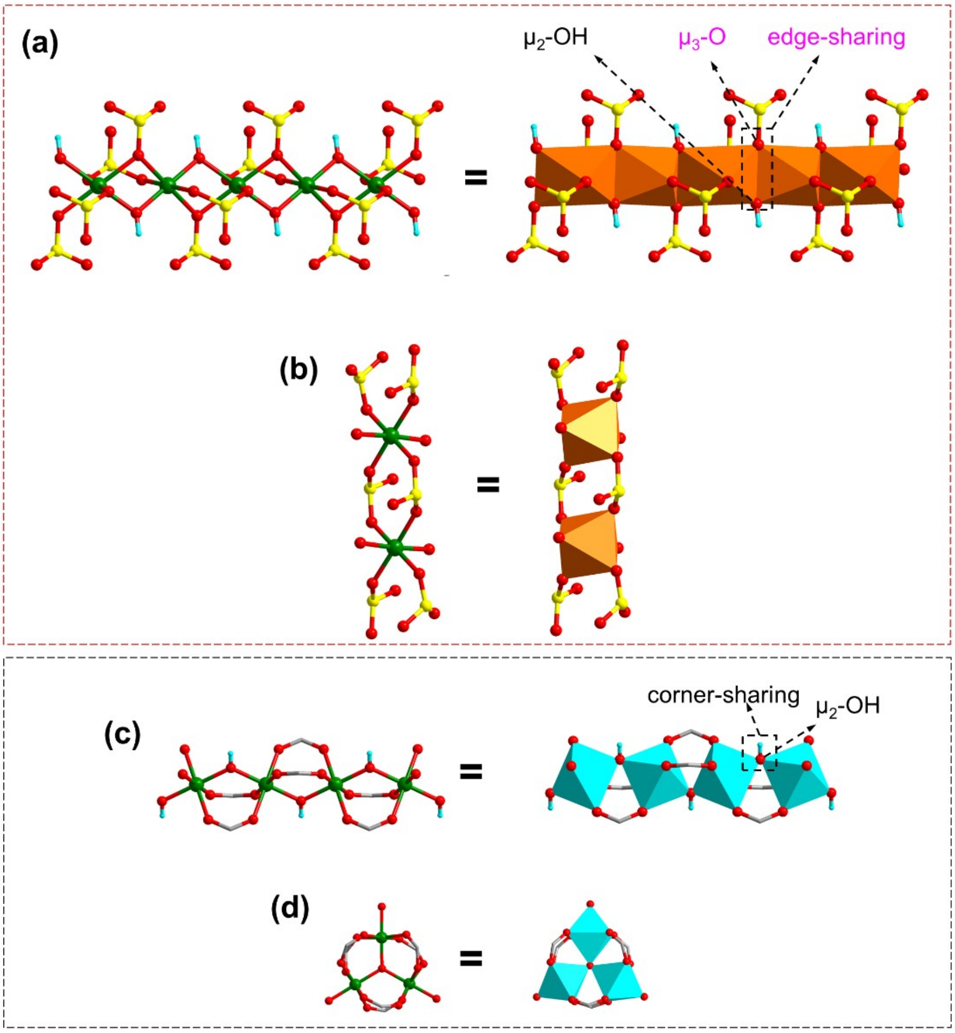

Fig. S7 Comparisons of the Cr-SBUs in this work and literatures. (a), the new 1D Cr-chain characterized by edge-sharing mode in TGU-9; (b), dinuclear Cr-SBU in TGU-10; (c), the reported corner-sharing 1D Cr-chain characterized by corner-sharing mode; (d), the reported Cr-trimer. Cr, green; C, gray; O, red; S, yellow; H, turquoise.

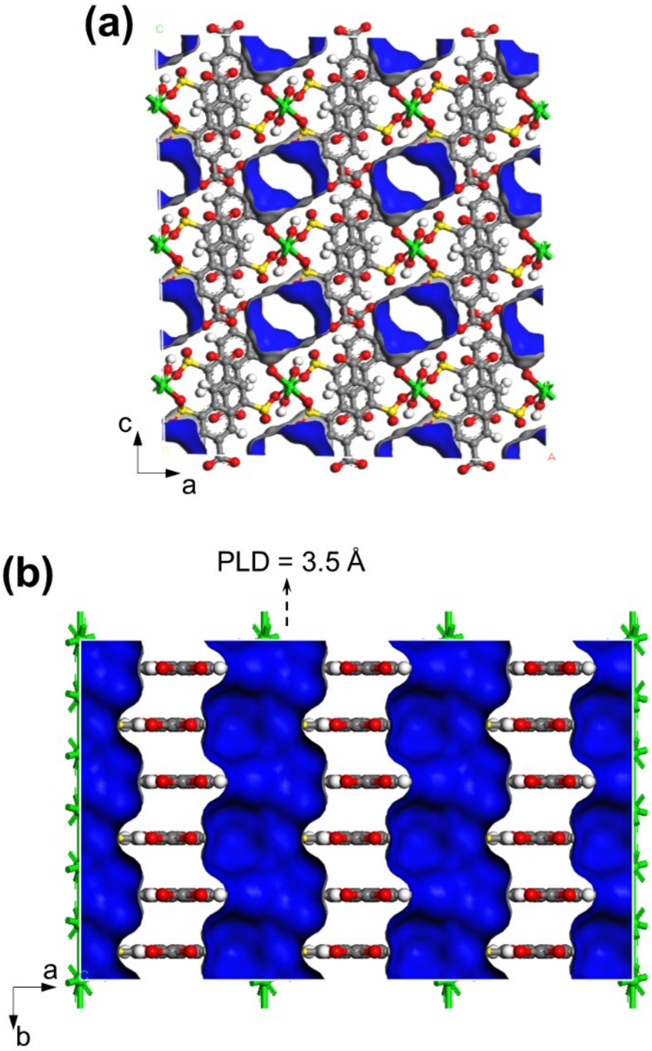

Fig. S8 The calculated pore limited diameter of TGU-9.

Table S3. The hydrogen bond information in TGU-9.

TGU-9
[1]+X,+Y,1+Z; [2]+X,+Y,-1+Z; [3]1+X,3/2-Y,+Z

D	H	A	d(D-H) (Å)	d(H-A) (Å)	d(D-A) (Å)	D-H-A (°)
O12	H12	O9[1]	0.94(2)	1.66(3)	2.60(2)	179(7)
O10	H10	O11[2]	0.94(2)	1.72(3)	2.66(2)	175(6)
O6	H6	O8[3]	0.95	2.59	3.09(3)	113.4

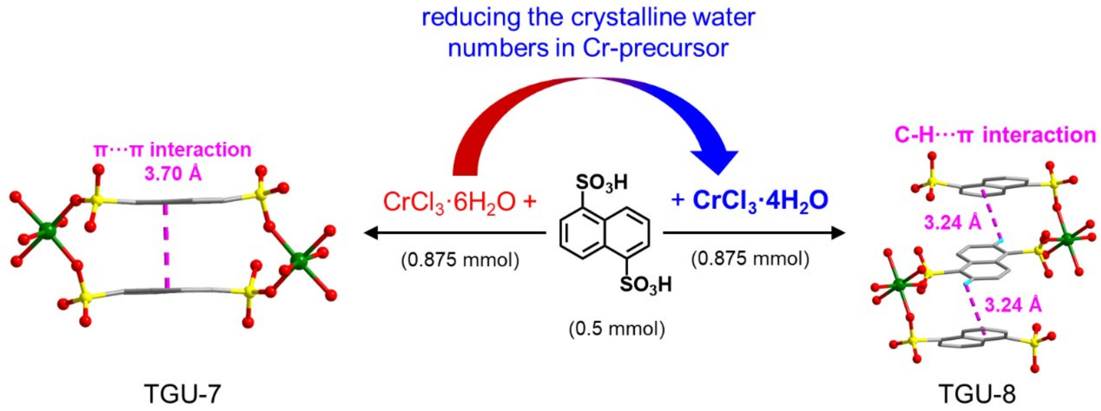

Fig. S9 Schematic illustration of reducing the chemical potential of crystalline water in Cr-precursor to assemble the new TGU-8 material by using $CrCl_3{\cdot}4H_2O$. Cr, green; C, gray; O, red; S, yellow; H atom of the coordinated H_2O and –OH, turquoise. H atoms on the ligands were omitted for clarity.

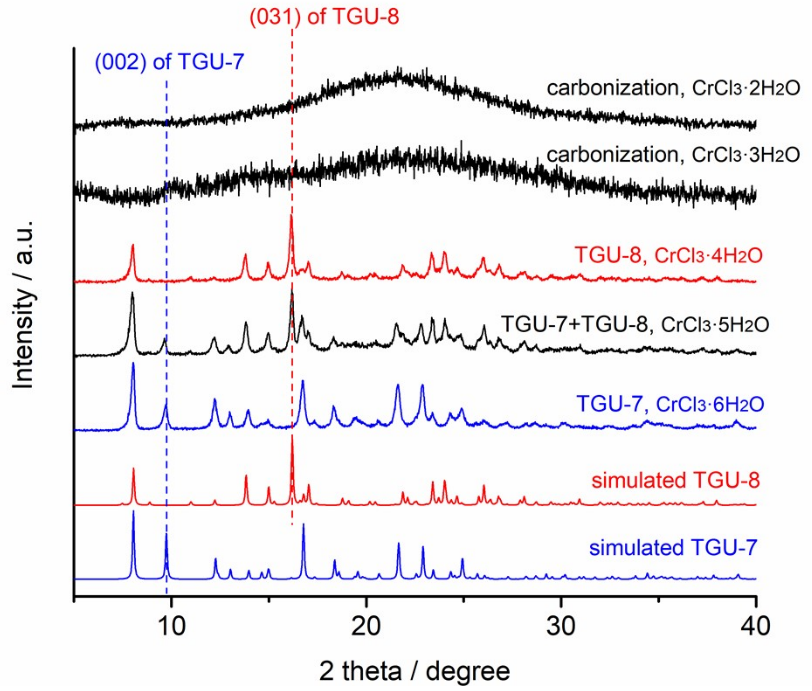

Fig. S10 PXRD patterns of the obtained phases using chromium chloride with different number of water molecules.

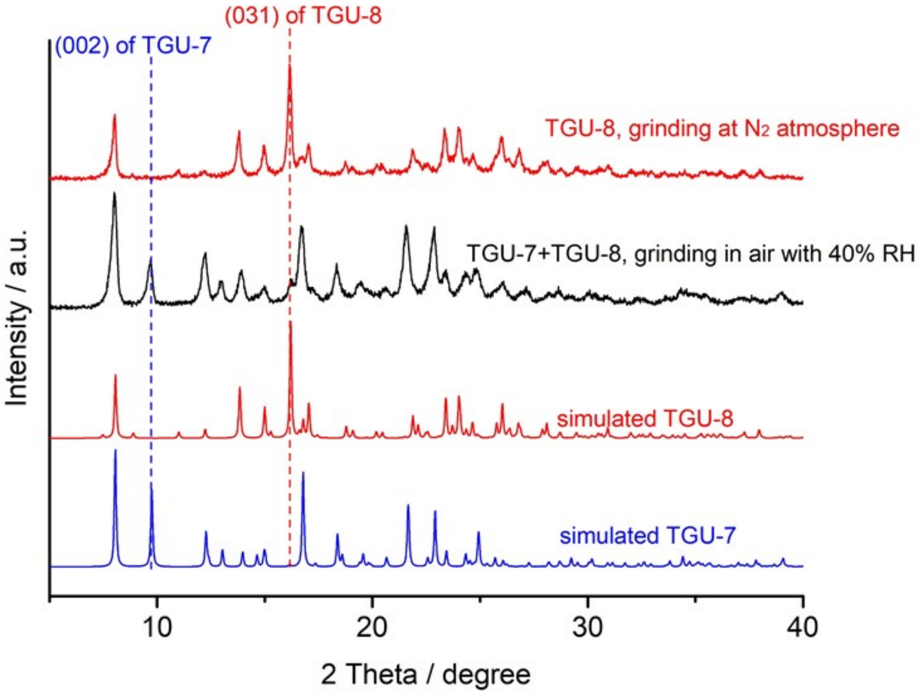

Fig. S11 Comparisons of the trace moisture effect on the synthesis of TGU-8.

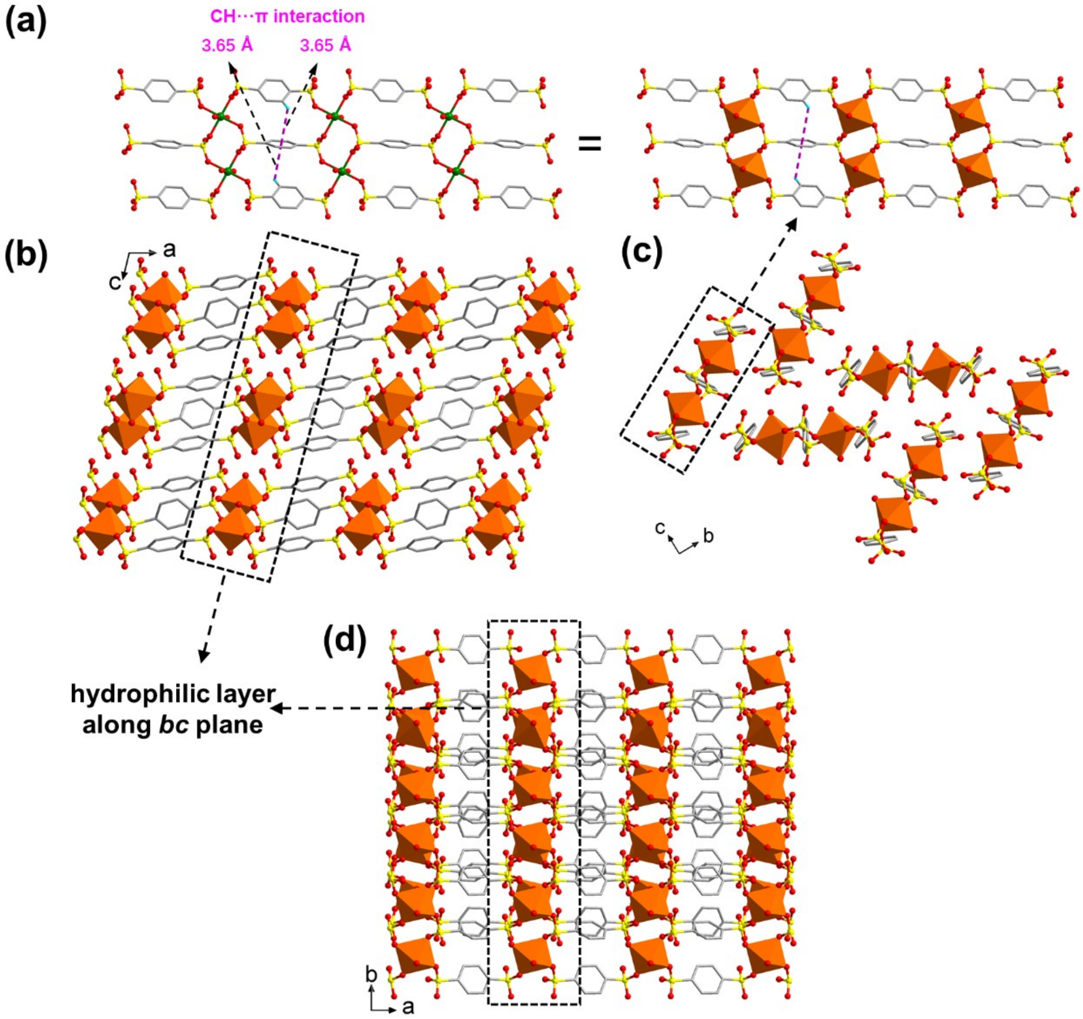

Fig. S12 The structure of TGU-10 with hydrophilic layer along the *bc* plane. (a), the 1D Cr-belt with highlighted CH···π interaction; (b) the view from *b* axis; (c), the view from *a* axis; (d), the view from *c* axis. Cr, green; C, gray; O, red; S, yellow; H atom for the CH···π interaction, turquoise; Other H atoms were omitted for clarity.

Table S4. The hydrogen bond information in TGU-10.

TGU-10

[1]1-X,-1/2+Y,3/2-Z; [2]2-X,-1/2+Y,3/2-Z; [3]2-X,2-Y,1-Z; [4]1-X,2-Y,1-Z

**D**	**H**	**A**	**d(D-H) (Å)**	**d(H-A) (Å)**	**d(D-A) (Å)**	**D-H-A (°)**
O8	H8A	O11[1]	0.87	1.98	2.82(2)	162.0
O8	H8B	O15[2]	0.87	2.03	2.66(2)	128.0
O9	H9A	O14[3]	0.84	2.00	2.560(18)	123.5
O9	H9B	O11[4]	0.85	2.31	3.06(3)	147.7

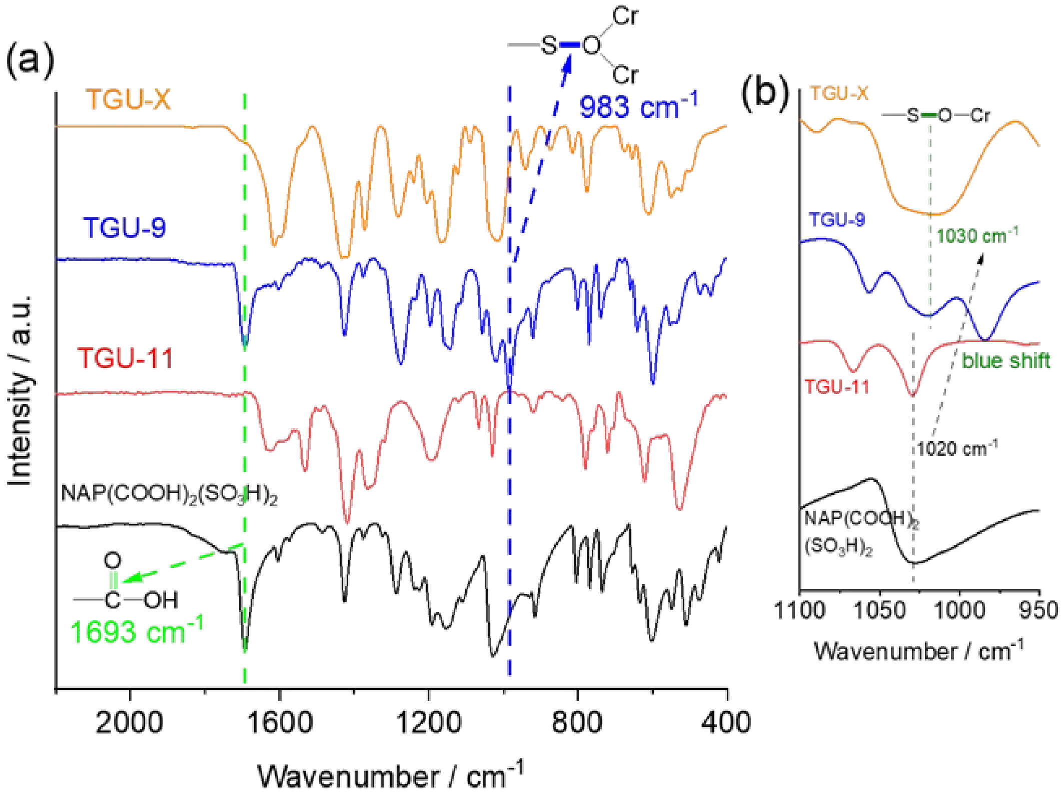

Fig. S13 FT-IR spectra of the TGU-*X*, TGU-9, TGU-11, and $NAP(COOH)_2(SO_3H)_2$ ligand. (a), The highlighted intense stretching vibration of the S−μ_3-O bond and the C=O stretching vibration; (b), the band shift of S−O bond in the bidentate Cr-SO_3 entity.

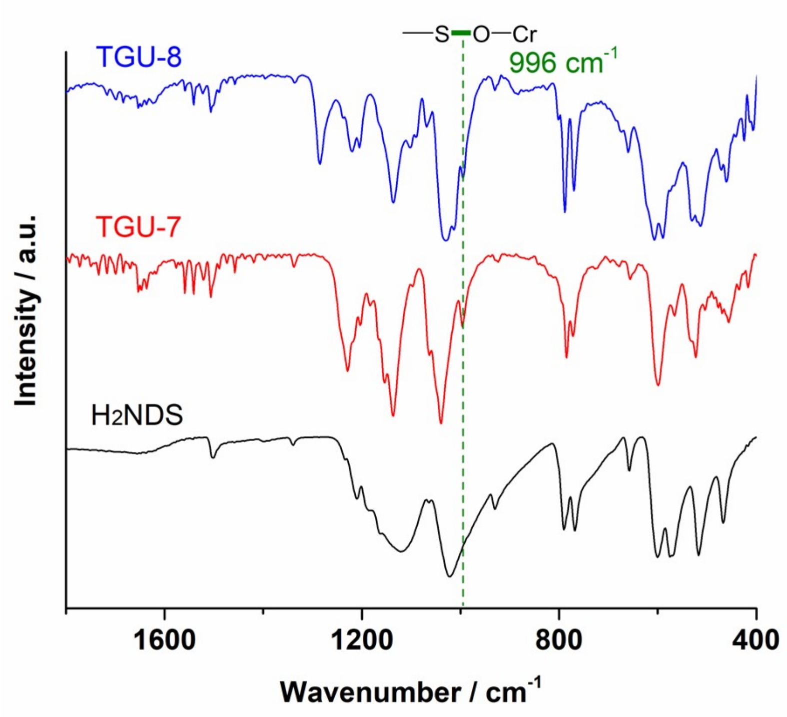

Fig. S14 FT-IR spectra of the TGU-7, TGU-8, and H_2NDS ligand.

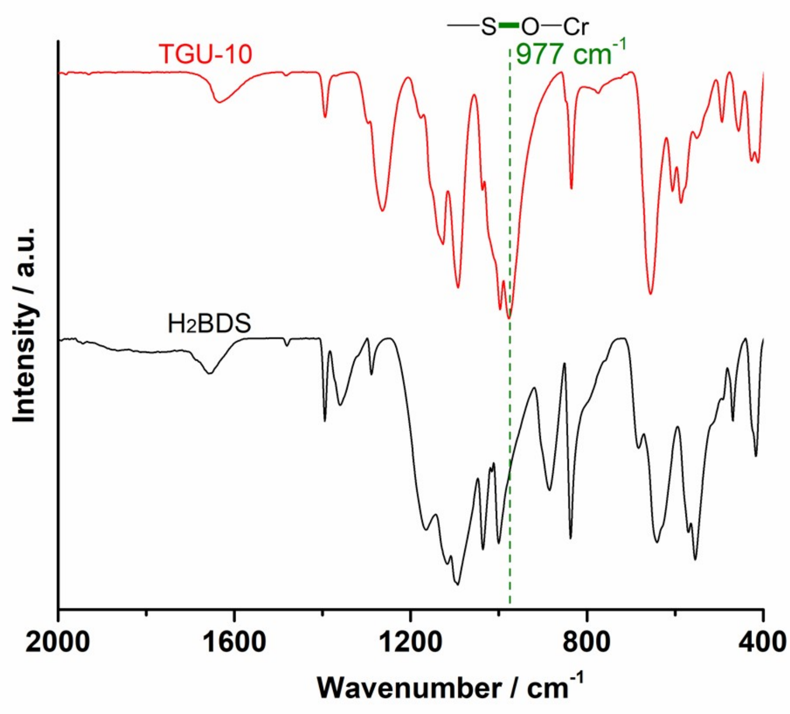

Fig. S15 FT-IR spectra of the TGU-10 and H_2BDS ligand.

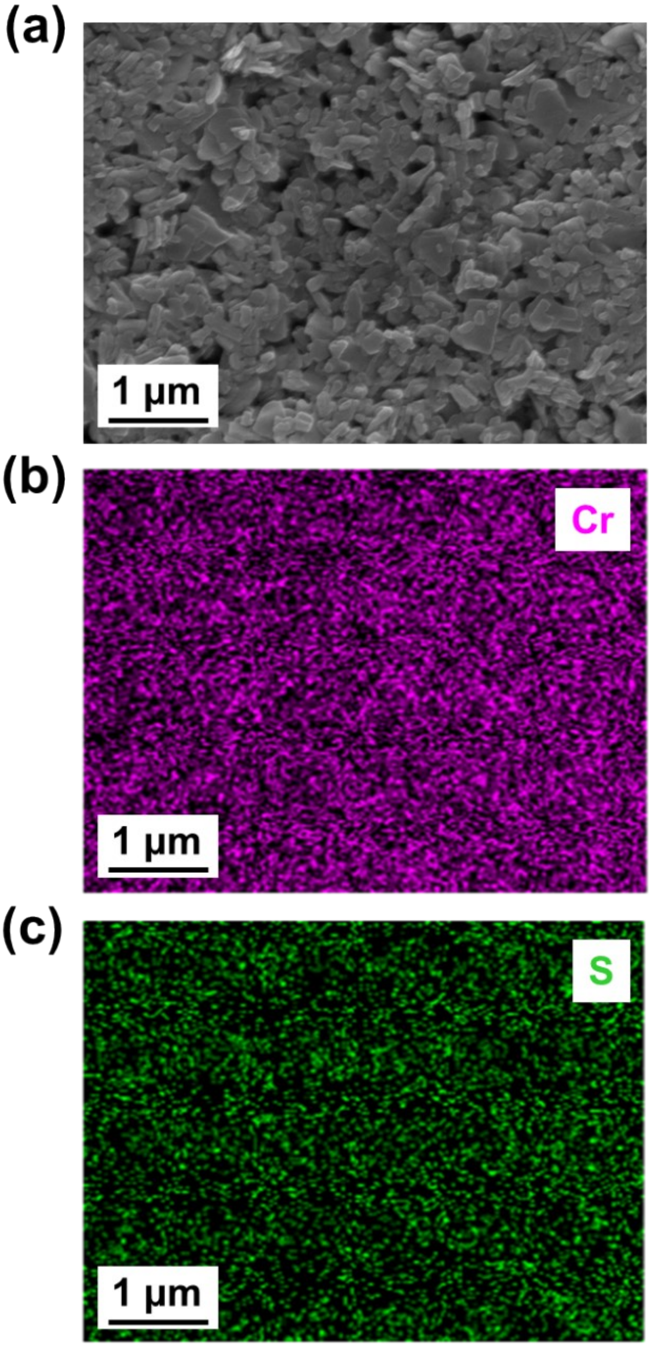

Fig. S16 The SEM image and EDS mapping of TGU-7. (a), the SEM image; (b), the element Cr distribution; (c), the element S distribution.

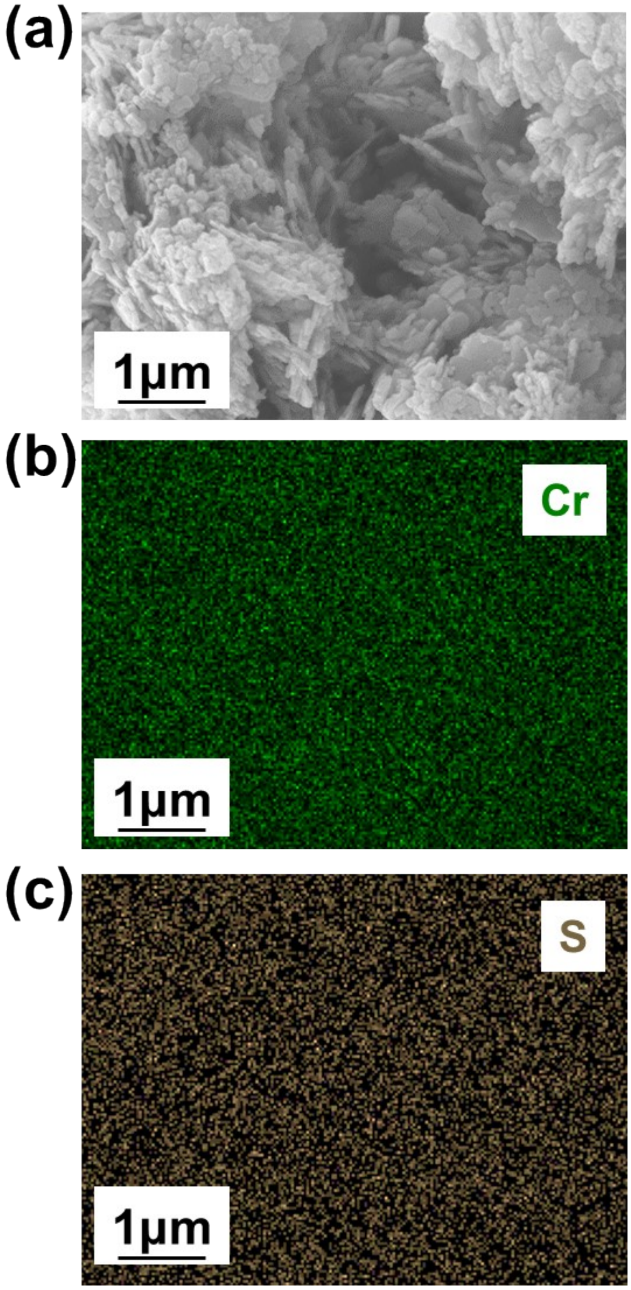

Fig. S17 The SEM image and EDS mapping of TGU-8. (a), the SEM image; (b), the element Cr distribution; (c), the element S distribution.

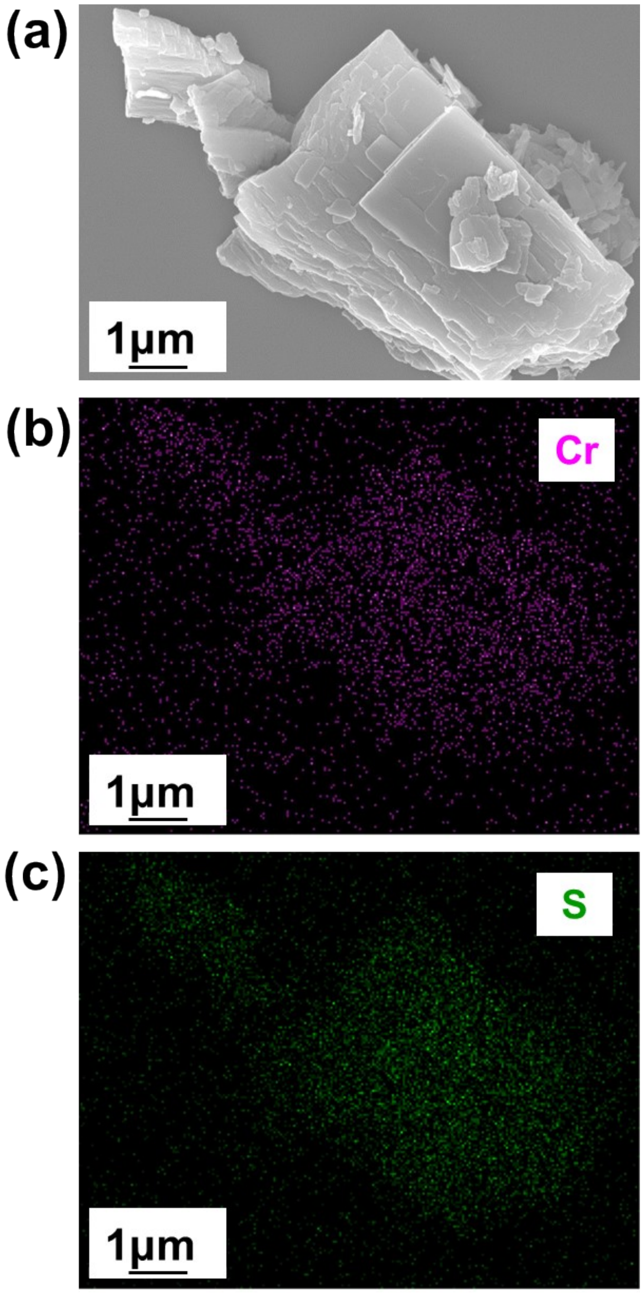

Fig. S18 The SEM image and EDS mapping of TGU-9. (a), the SEM image; (b), the element Cr distribution; (c), the element S distribution.

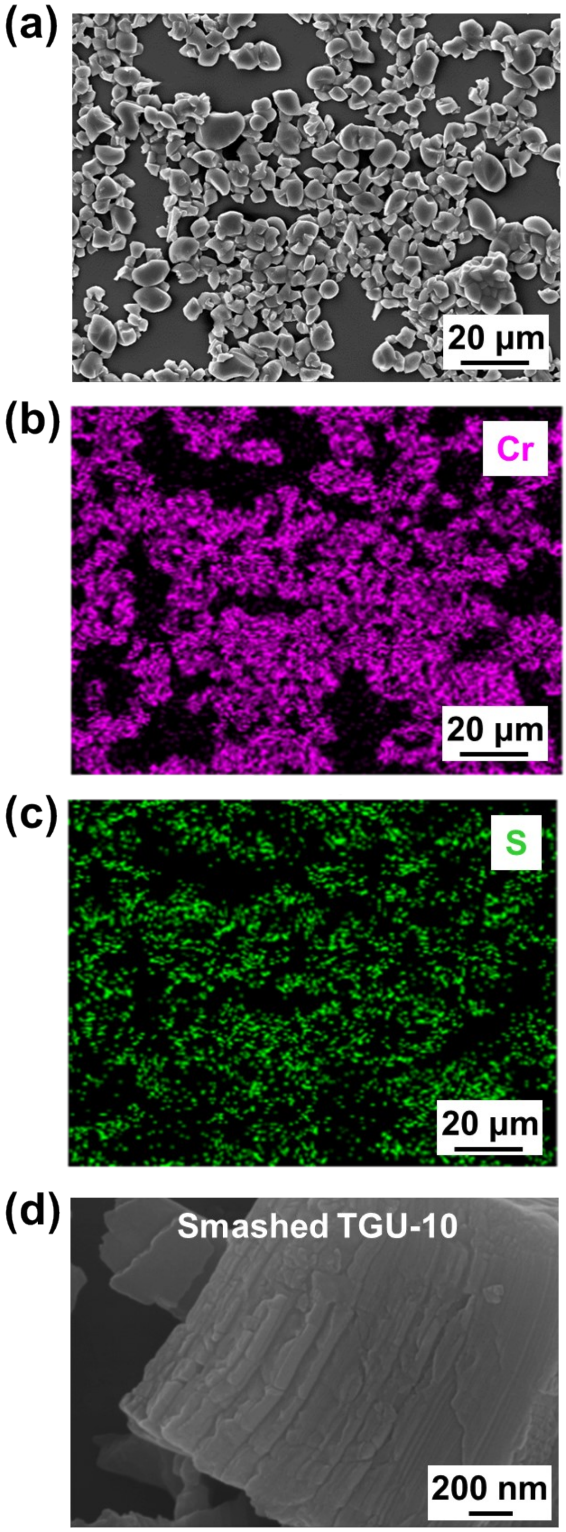

Fig. S19 The SEM images and EDS mapping of TGU-10. (a) and (d), SEM images of the fresh-made and smashed TGU-10, respectively; (b), the element Cr distribution; (c), the element S distribution.

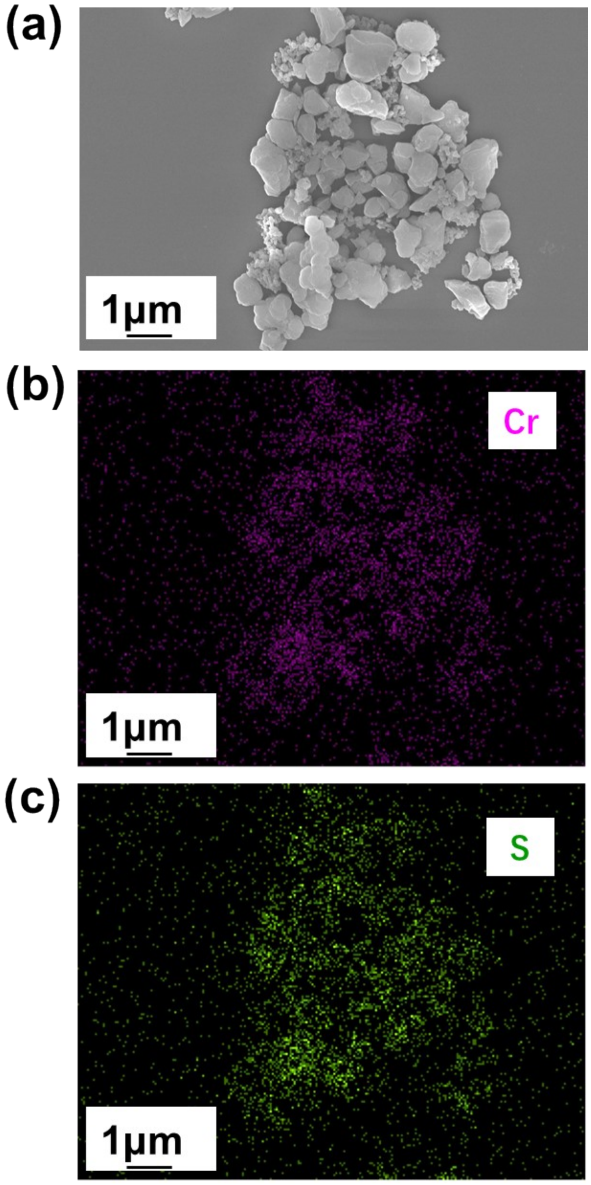

Fig. S20 The SEM image and EDS mapping of TGU-11. (a), the SEM image; (b), the element Cr distribution; (c), the element S distribution.

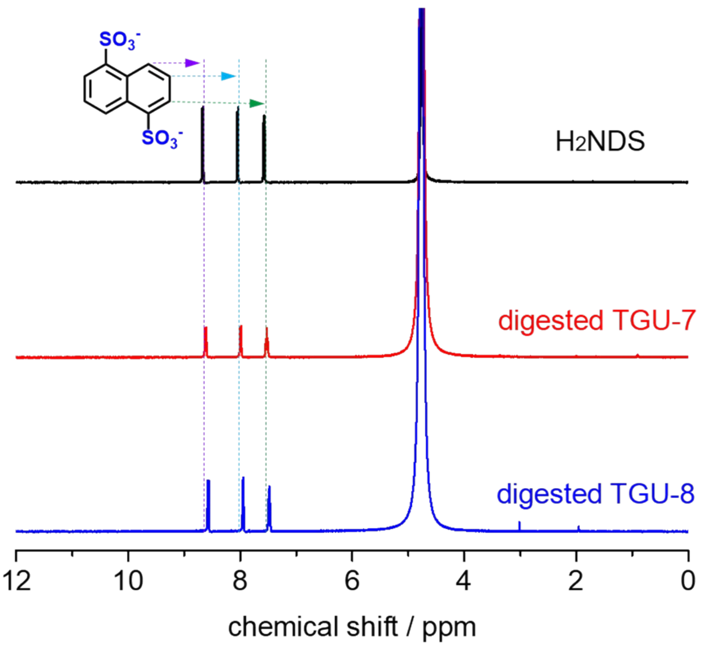

Fig. S21 ^{1}H NMR spectra of the digested TGU-7, digested TGU-8 and H_2NDS ligand in 6 M KOH/D_2O solution.

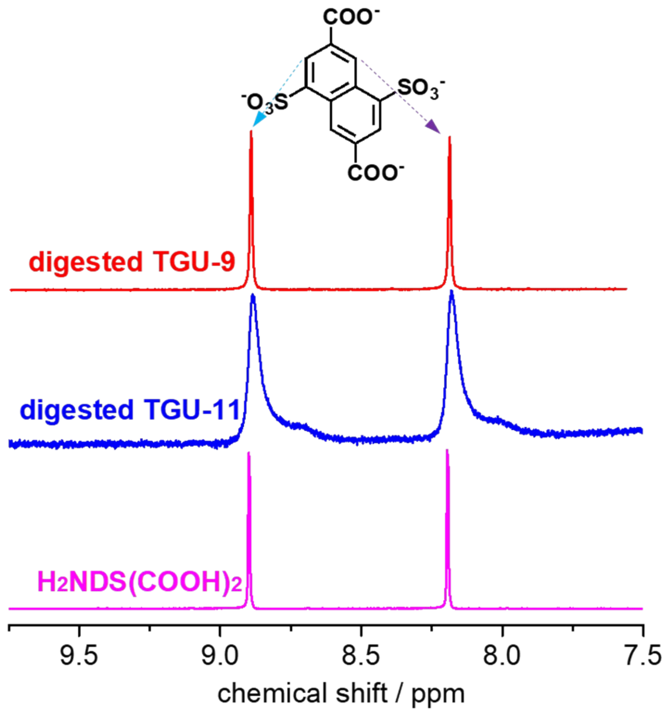

Fig. S22 ^{1}H NMR spectra of the digested TGU-9, digested TGU-11, and $NAP(COOH)_2(SO_3H)_2$ in 6 M KOH/D_2O solution.

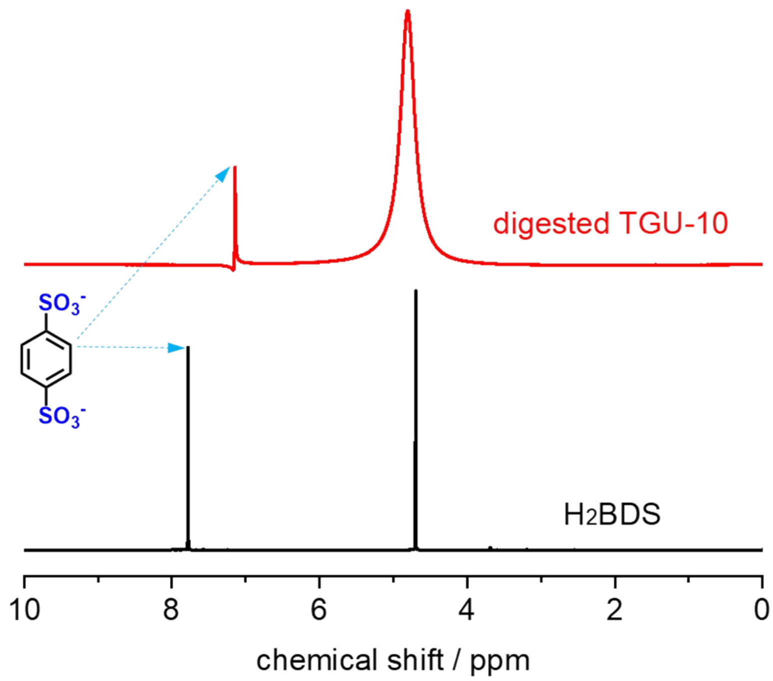

Fig. S23 ^{1}H NMR spectra of the digested TGU-10 and H_2BDS ligand in 6 M KOH/D_2O solution.

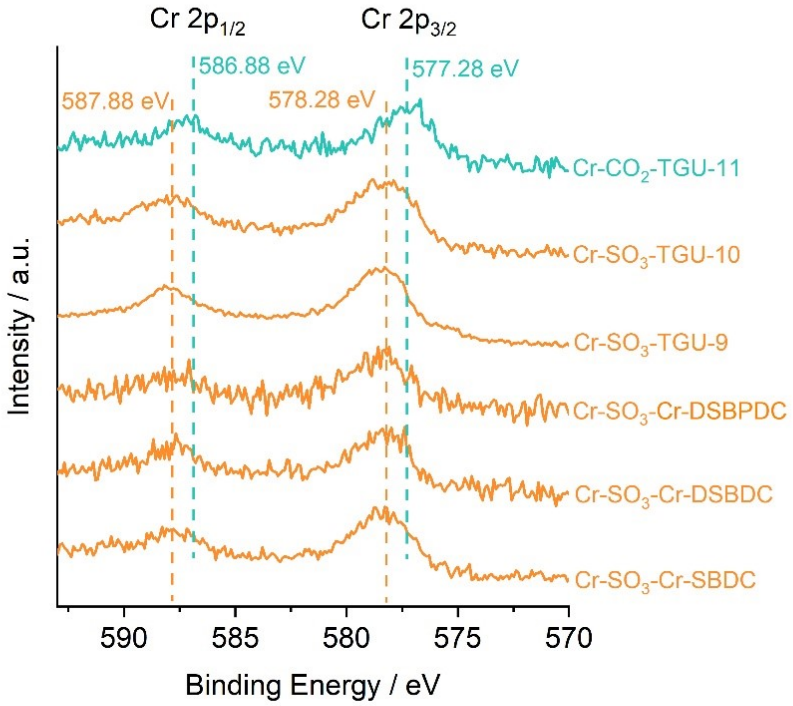

Fig. S24 XPS of the Cr-CO_2 coordinated TGU-11 and the Cr-SO_3 coordinated TGU-10, TGU-9, Cr-DSBPDC, Cr-DSBDC, and Cr-SBDC.

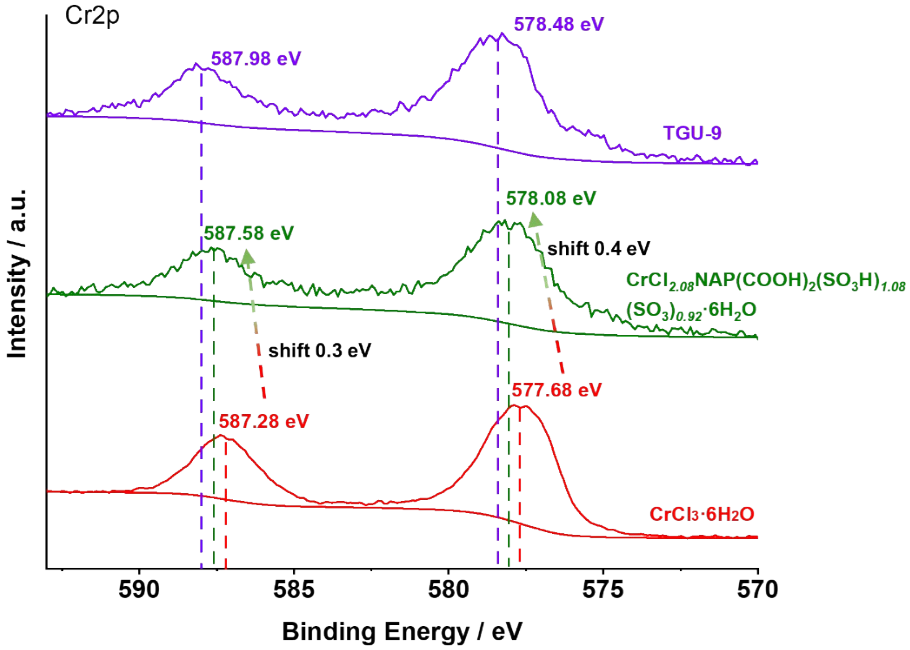

Fig. S25 The binding energy of Cr2p in $CrCl_3{\cdot}6H_2O$, $CrCl_{2.08}NAP(COOH)_2(SO_3H)_{1.08}(SO_3)_{0.92}{\cdot}6H_2O$ ($0<x<2$), and TGU-9.

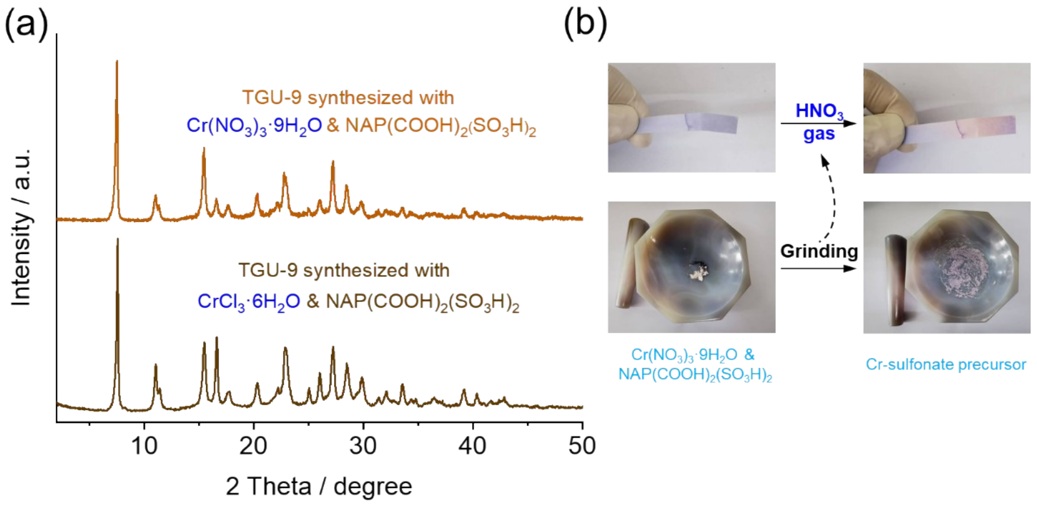

Fig. S26 (a) PXRD pattern of TGU-9 prepared with $Cr(NO_3)_3 \cdot 9H_2O$ and (b) visualized detection of HNO_3 gas during the grinding of $Cr(NO_3)_3 \cdot 9H_2O$ with the $NAP(COOH)_2(SO_3H)_2$ ligand.

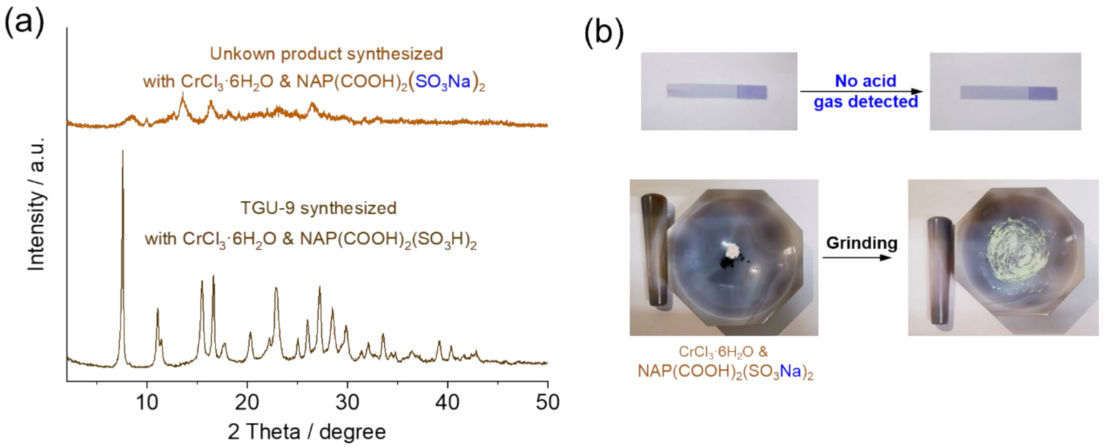

Fig. S27 (a), Unknown product synthesized with $NAP(COOH)_2(SO_3Na)_2$ and $CrCl_3 \cdot 6H_2O$; (b), no acid gas detected during the grinding of the $NAP(COOH)_2(SO_3Na)_2$ with $CrCl_3 \cdot 6H_2O$.

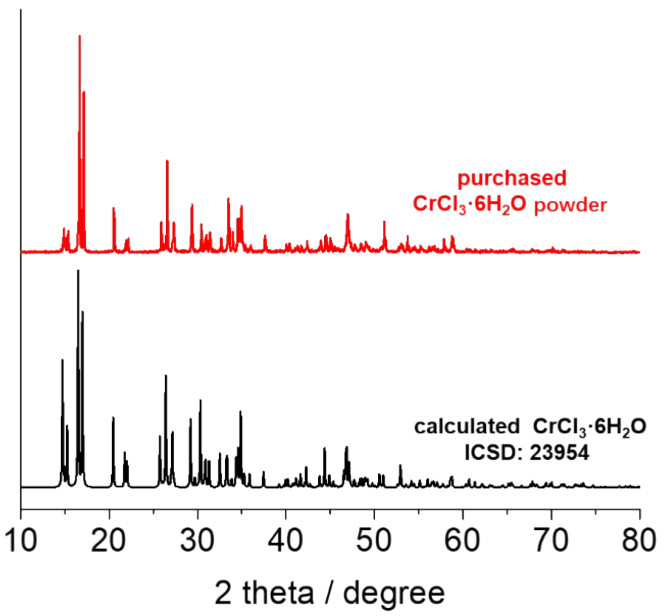

Fig. S28 The identification of the purchased $CrCl_3{\cdot}6H_2O$ used in this work.

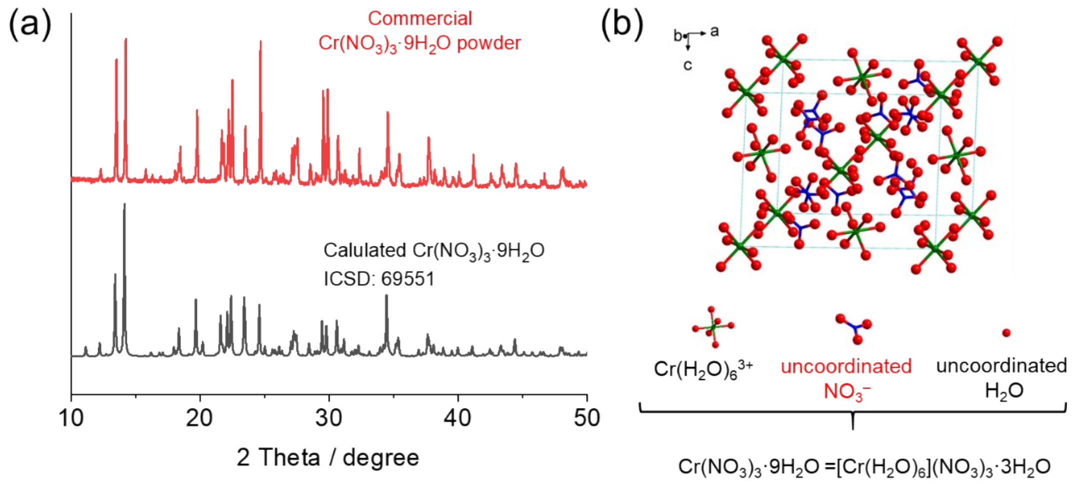

Fig. S29 Identification of the adopted $Cr(NO_3)_3{\cdot}9H_2O$ (a) and the corresponding unit cell (b).

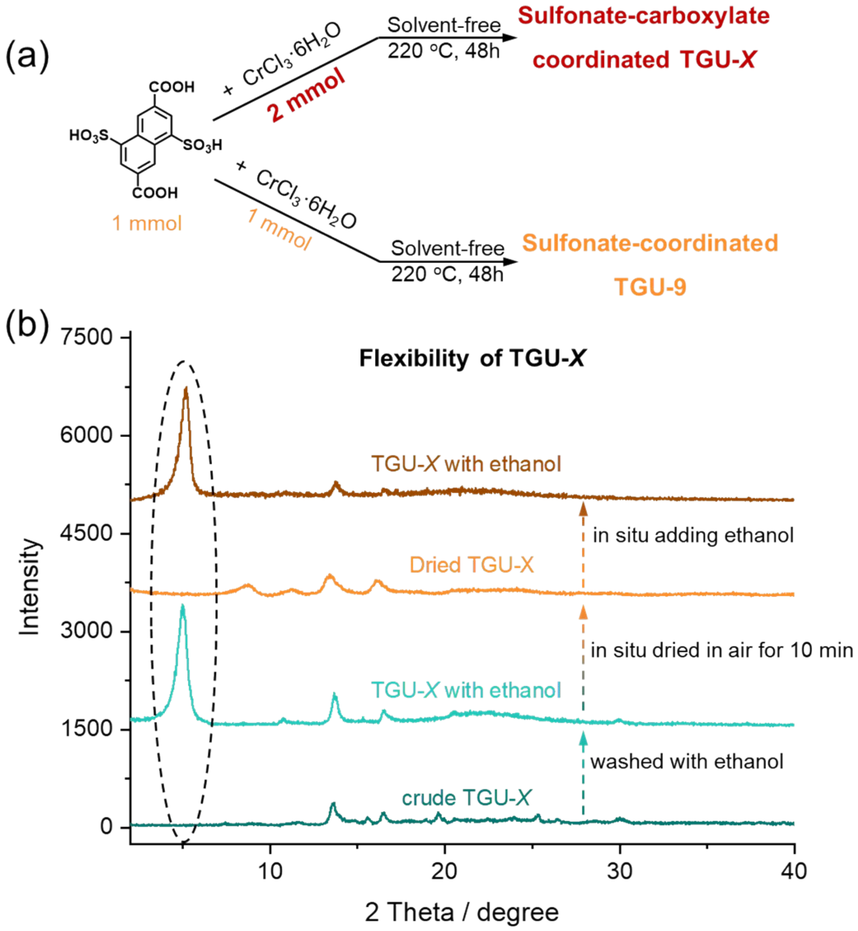

Fig. 30 Synthesis of TGU-X (a) and the flexibility test under different conditions (b).

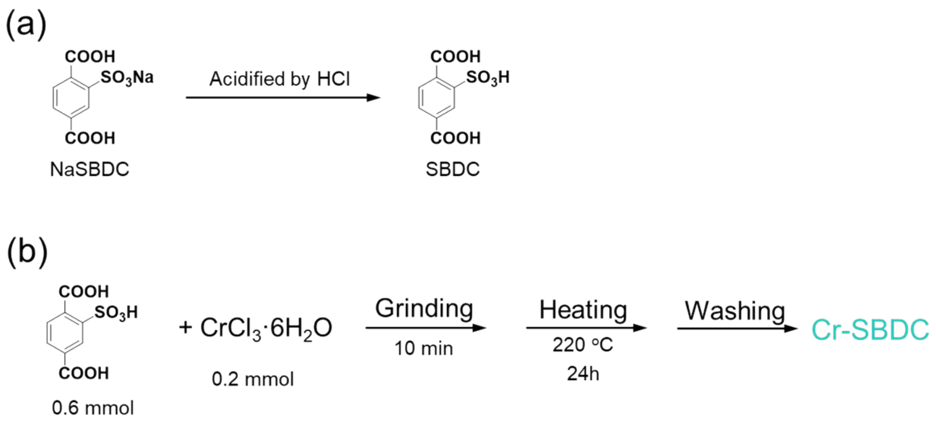

Scheme S1. Synthesis route of Cr-SBDC. (a), Synthesis of the SBDC ligand. (b), Synthesis of Cr-SBDC.

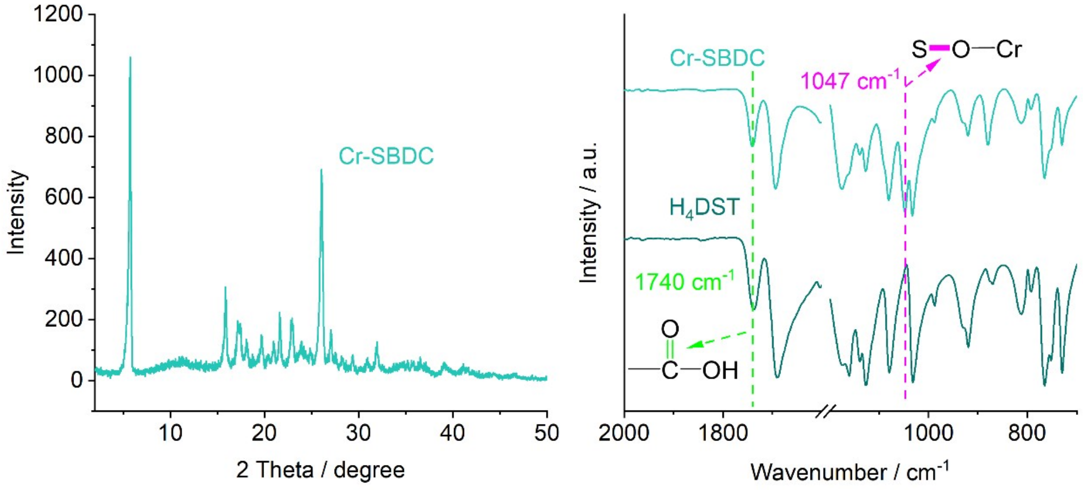

Fig. S31 Synthesis and characterization of Cr-SBDC. (a), Synthesis of Cr-SBDC. (b), PXRD pattern of Cr-SBDC. (c), FT-IR spectra of Cr-SBDC and SBDC.

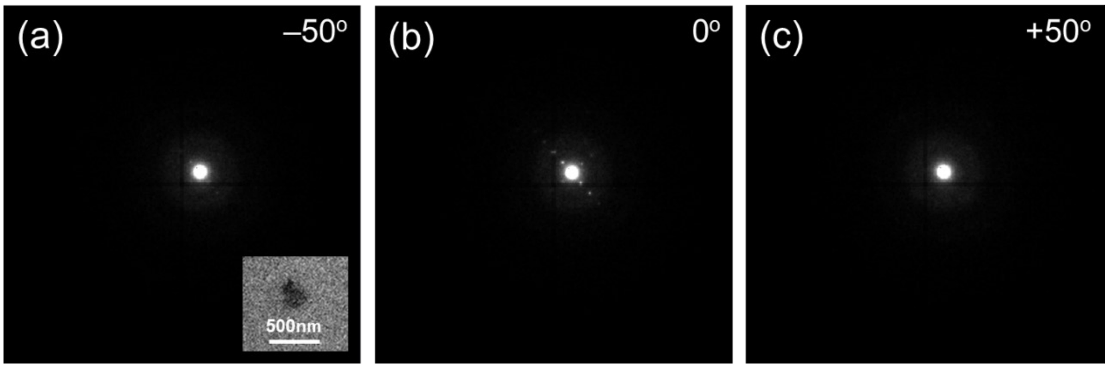

Fig. S32 3D ED data of of Cr-SBDC collected from –50° to +50°. (a), –50°; (b), 0°; (c), +50°. The insertion in (a) is the corresponding TEM image.

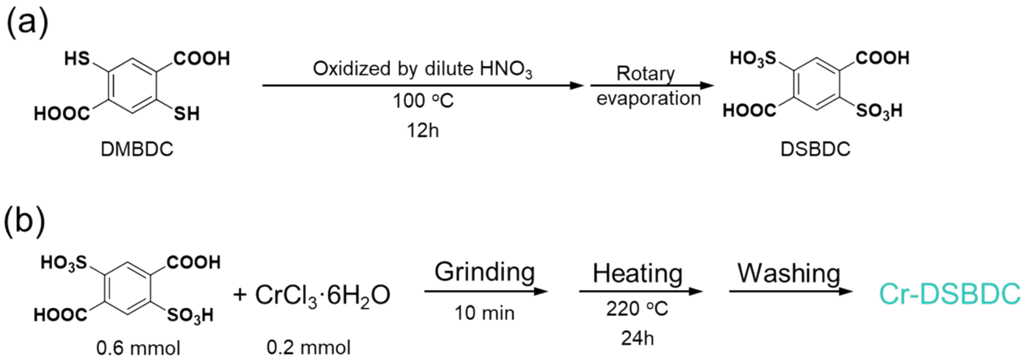

Scheme S2. Synthesis route of Cr-DSBDC. (a), Synthesis of the DSBDC ligand. (b), Synthesis of Cr-DSBDC.

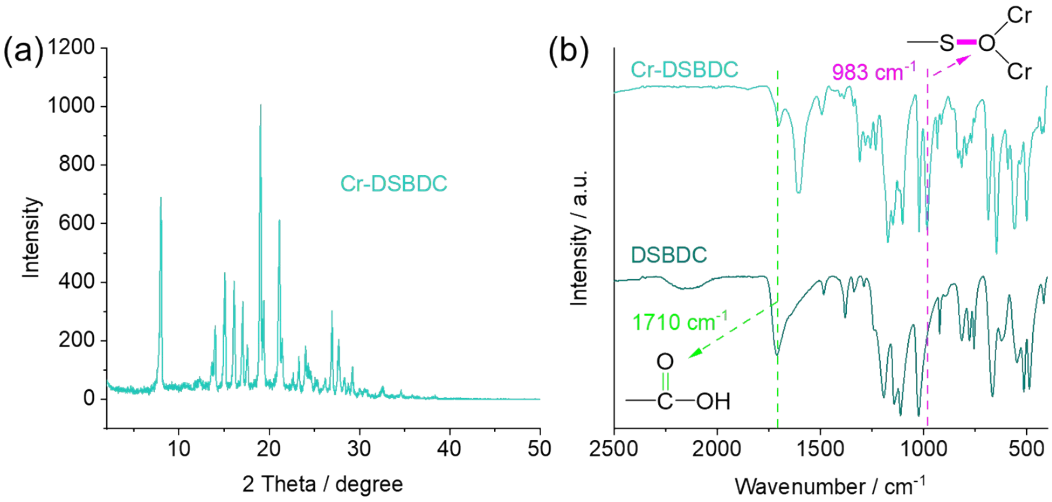

Fig. S33 Synthesis and characterization of Cr-DSBDC. (a), PXRD pattern of Cr-DSBDC. (c), FT-IR spectra of Cr-DSBDC and DSBDC.

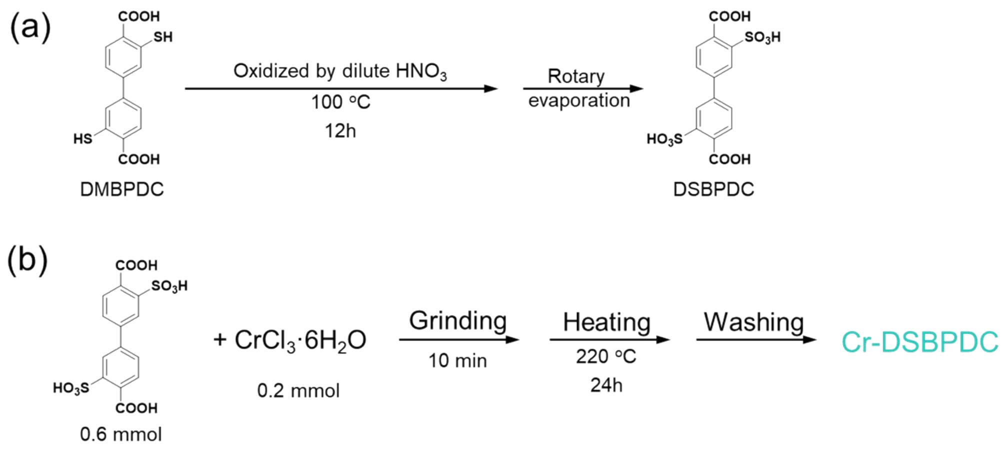

Scheme S3. Synthesis Route of Cr-DSBPDC. (a), Synthesis of DSBPDC. (b), Synthesis of Cr-DSBPDC.

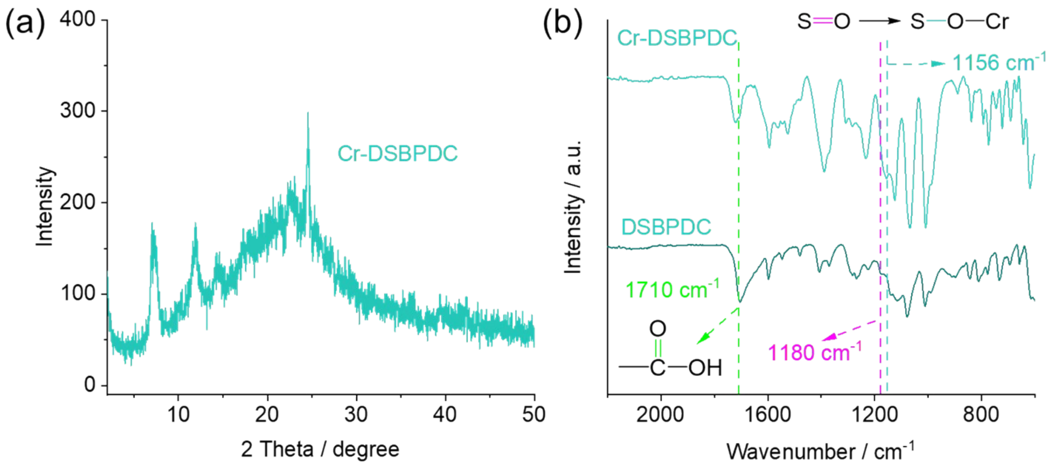

Fig. S34 Synthesis and characterization of Cr-DSBPDC. (a), PXRD pattern of Cr-DSBPDC. (b), FT-IR spectra of Cr-DSBPDC and DSBPDC.

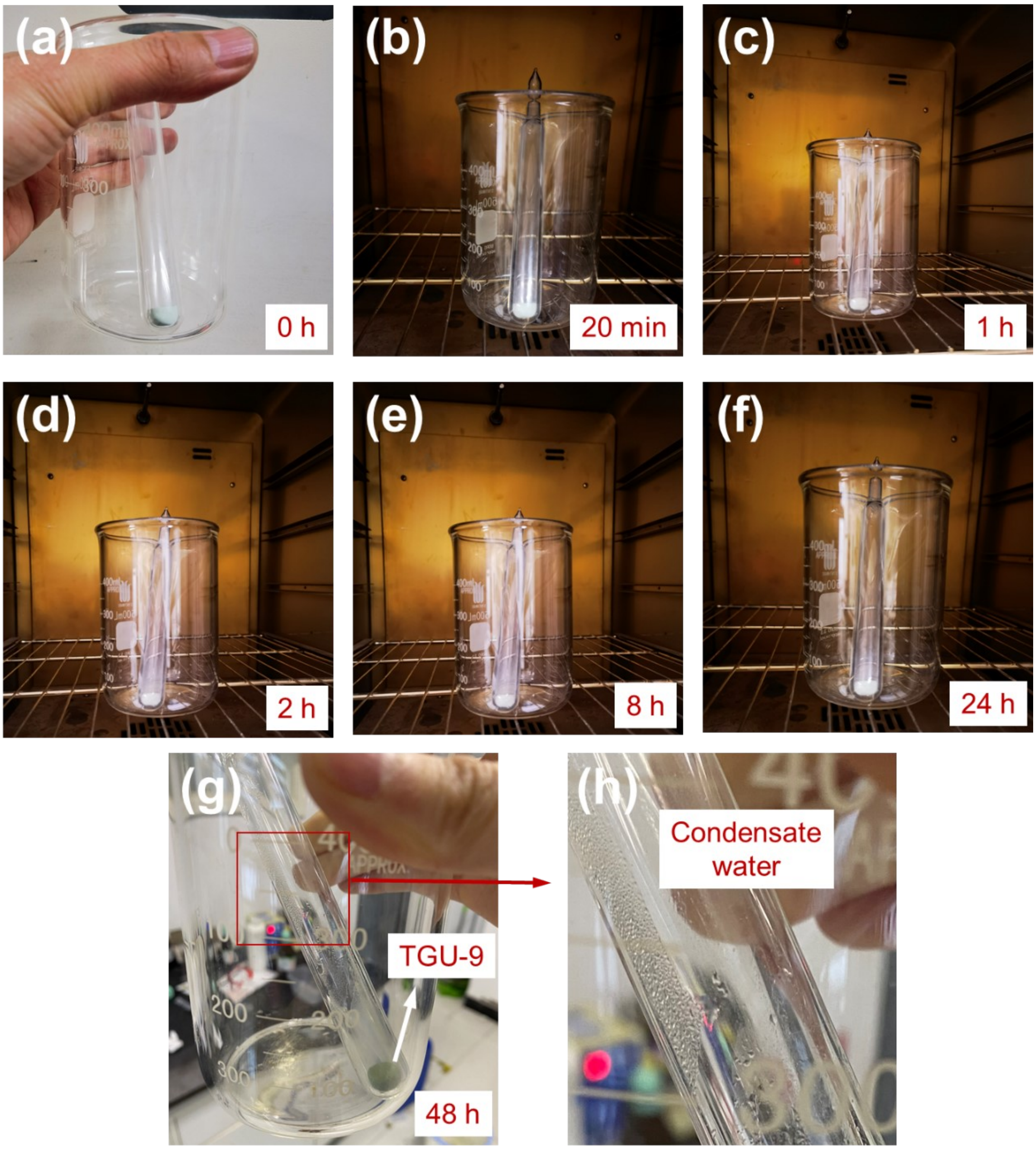

Fig. S35 The solid state of the ground reactant mixture sealed in a glass tube during heating at 220 °C for different hours. (a), 0 h; (b), 20 min; (c), 1 h; (d), 2 h; (e), 8 h; (f), 24 h; (g), after heating at 220 °C for 48 h and cooled to room temperature. The condensate water product can also be clearly observed.

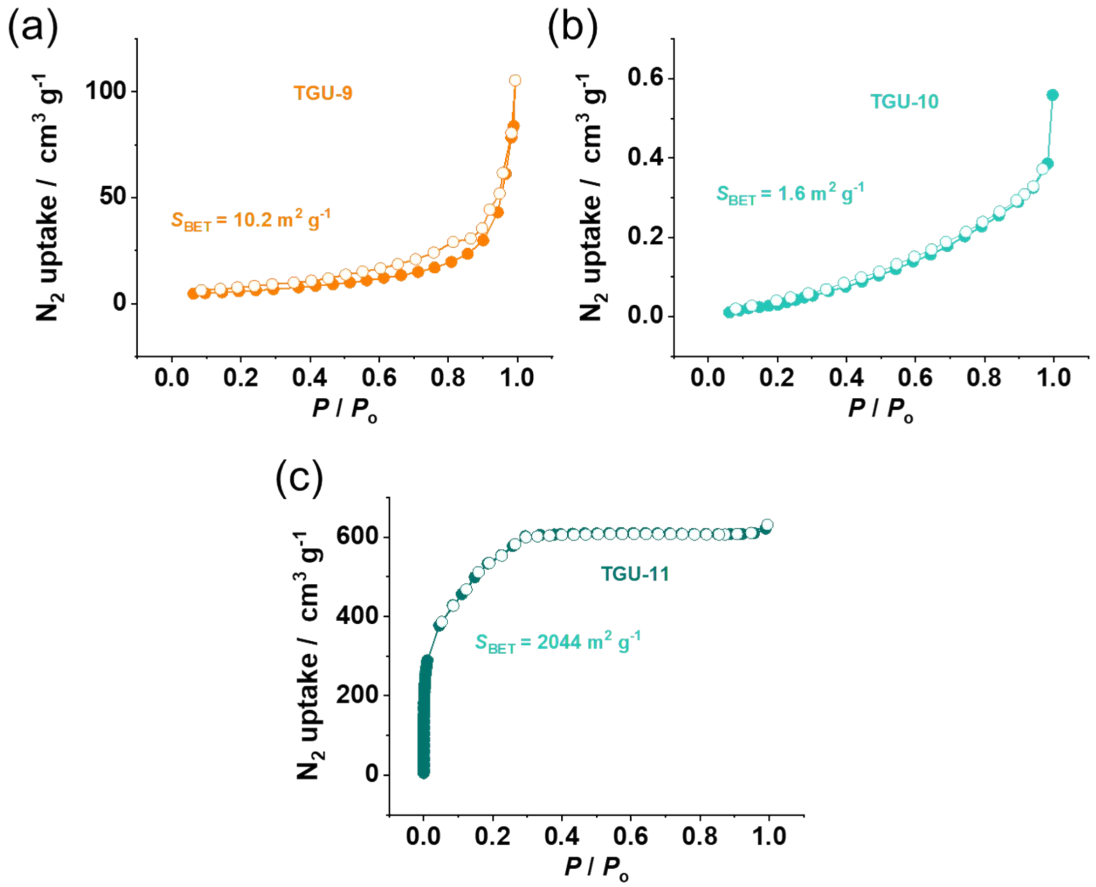

Fig. S36 N_2 adsorption and desorption isotherms of TGU-9 (a), TGU-10 (b), and TGU-11 (c). Closed and open symbol represent adsorption and desorption, respectively.

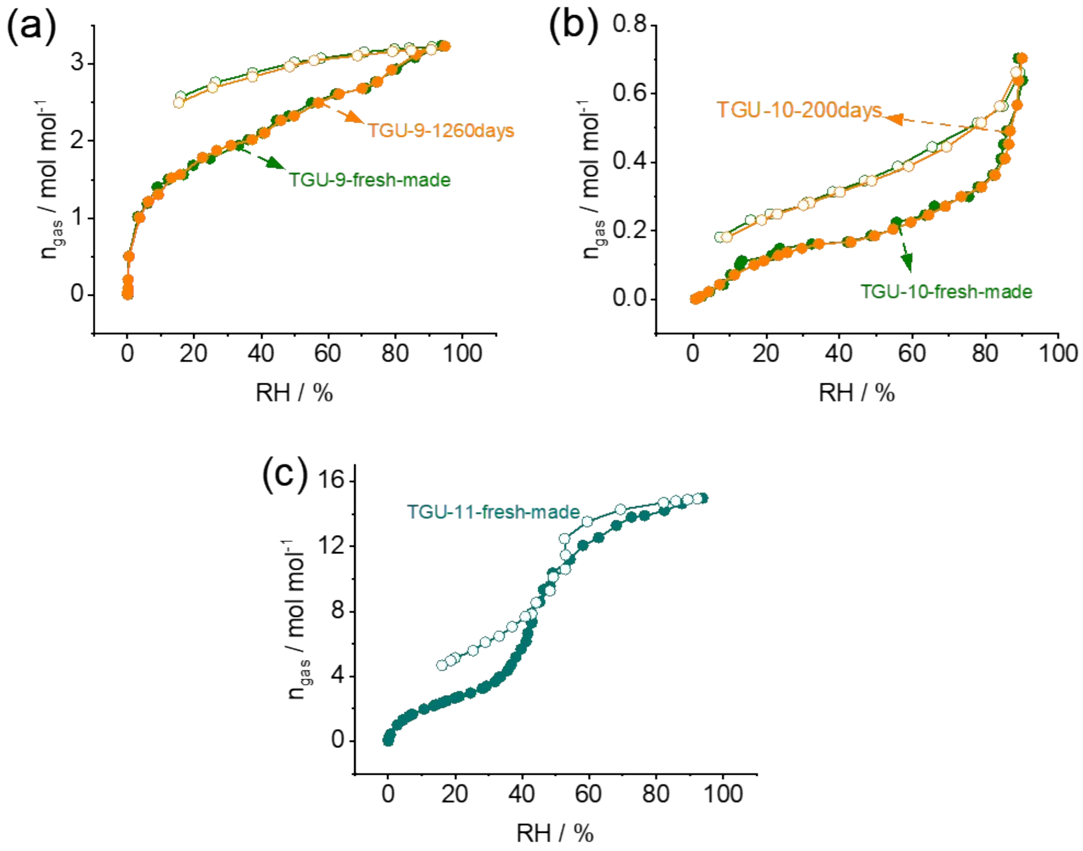

Fig. S37 Water vapor uptake comparison of the fresh-made and the long-time stored TGU-9 (a), TGU-10 (b) and TGU-11 (c, the fresh-made). Closed and open symbol represent adsorption and desorption, respectively.

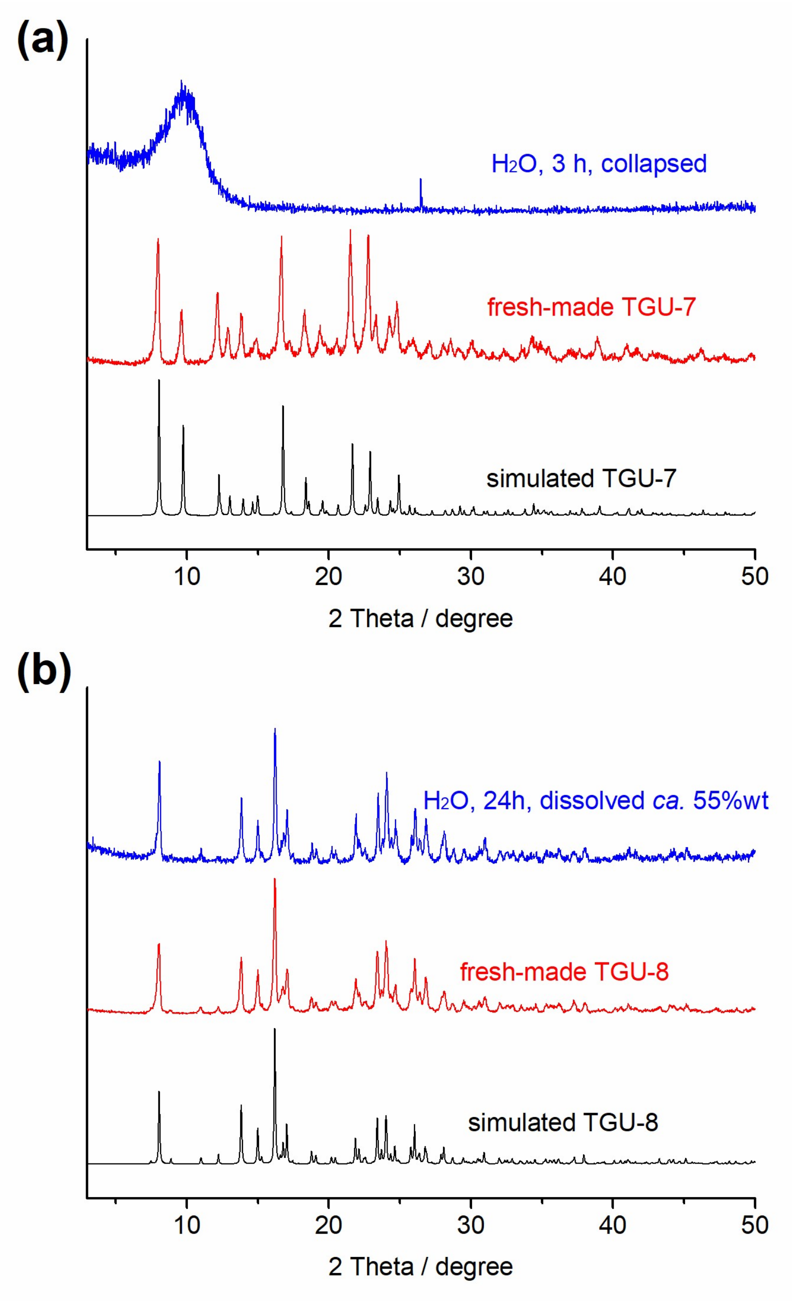

Fig. S38 Stabilities of TGU-7 and TGU-8 in water. (a), TGU-7; (b), TGU-8.

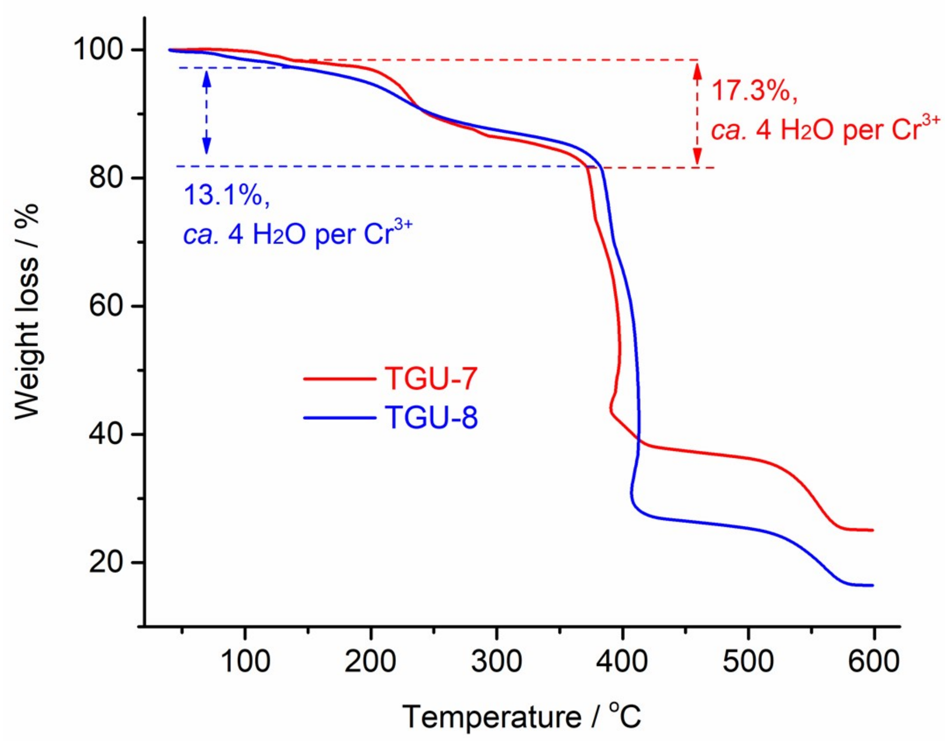

Fig. S39 TG curves of TGU-7 (red) and TGU-8 (blue) under air atmosphere.

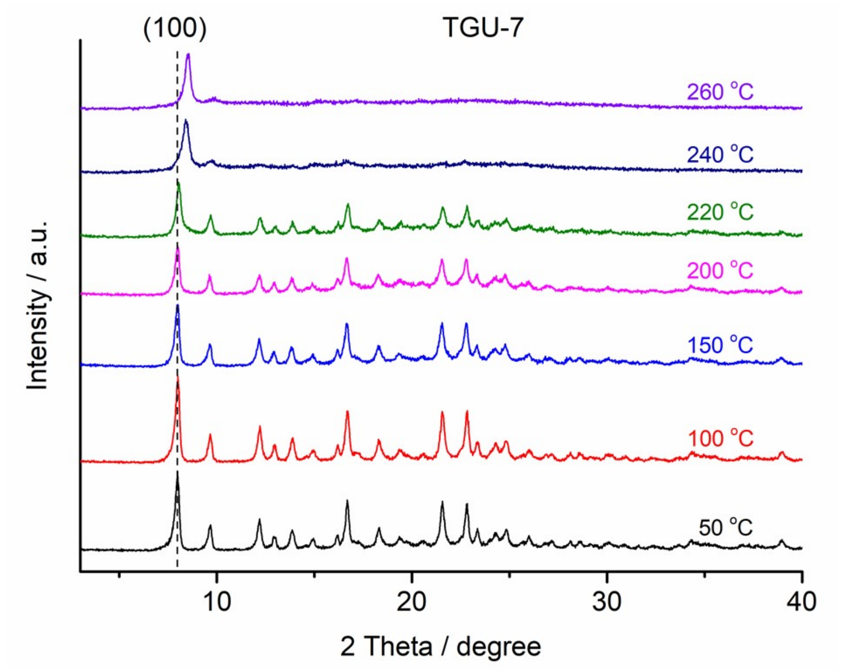

Fig. S40 Temperature-dependent *in-situ* PXRD patterns of TGU-7.

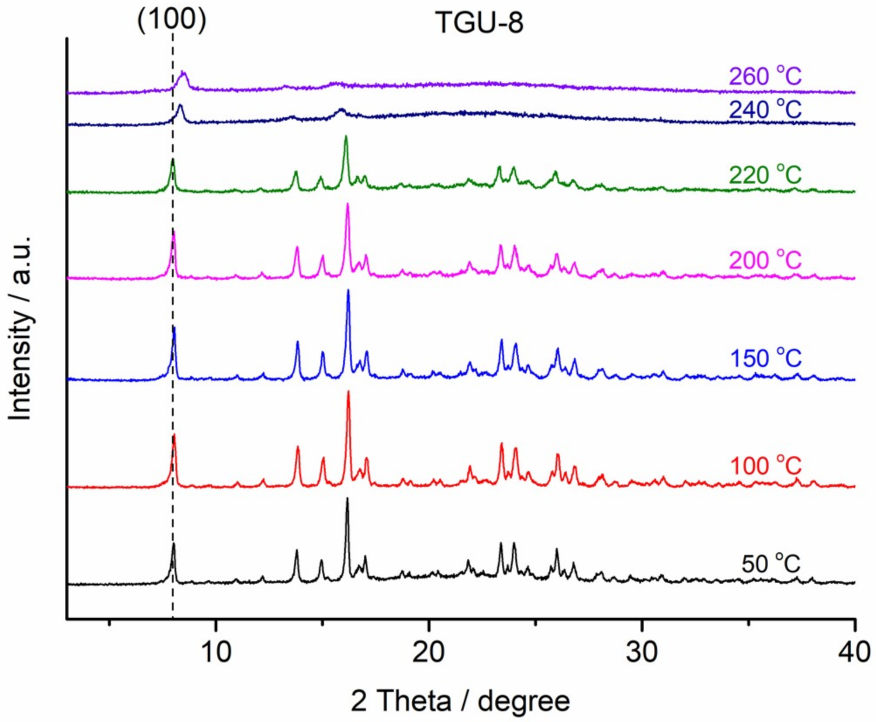

Fig. S41 Temperature-dependent *in-situ* PXRD patterns of TGU-8.

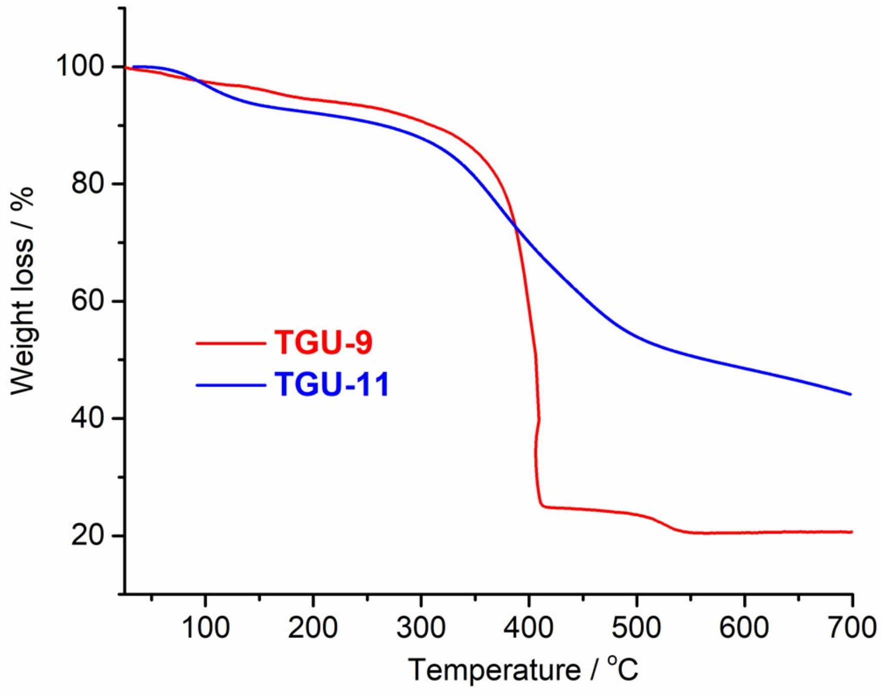

Fig. S42 TG curves of TGU-9 (red) and TGU-11 (blue) under air atmosphere.

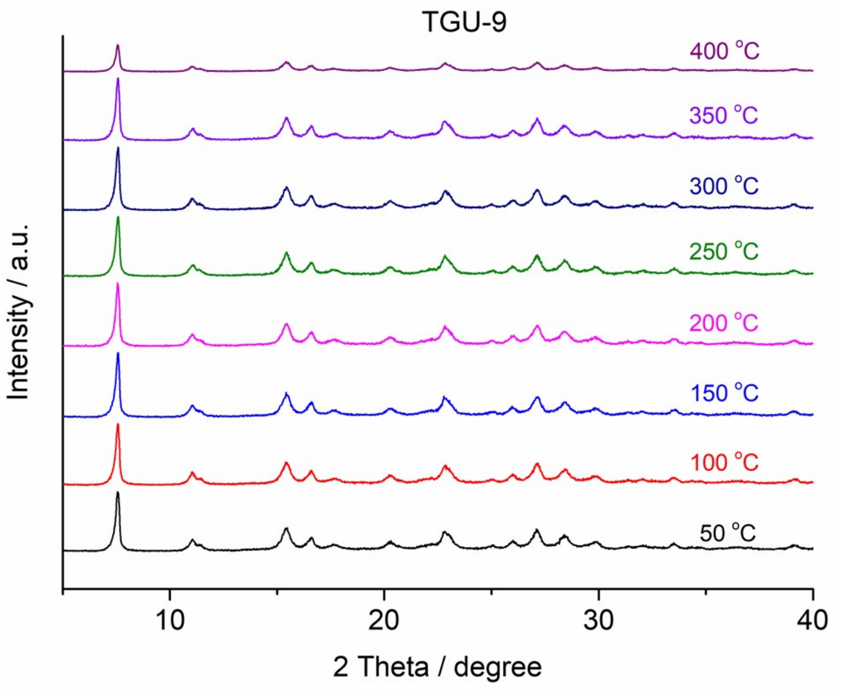

Fig. S43 Temperature-dependent *in-situ* PXRD patterns of TGU-9.

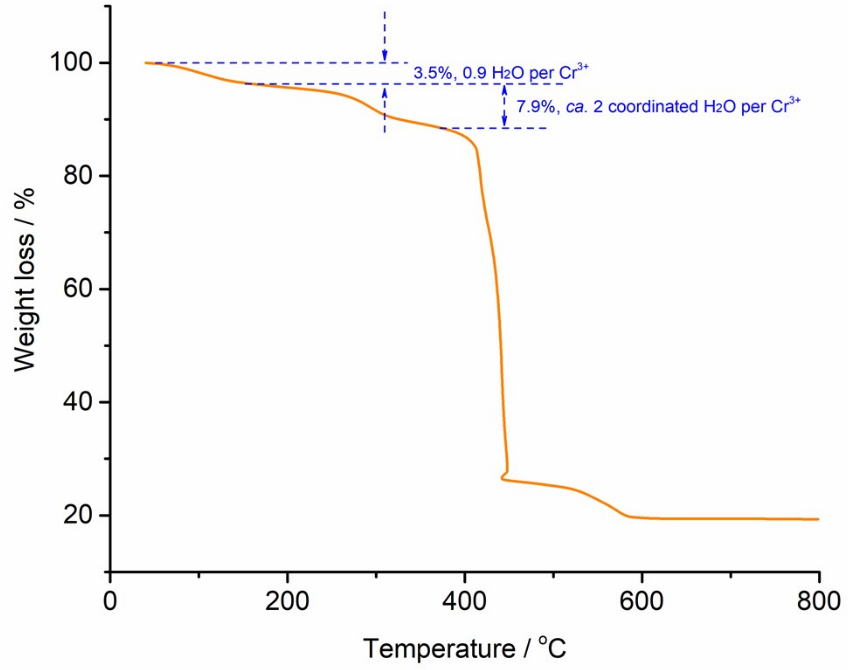

Fig. S44 TG curves of TGU-10 with fully adsorbed water vapor. Before TG test, TGU-10 was put in a closed environment with 100 % RH for 24 hours to guarantee the fully adsorption of water vapor.

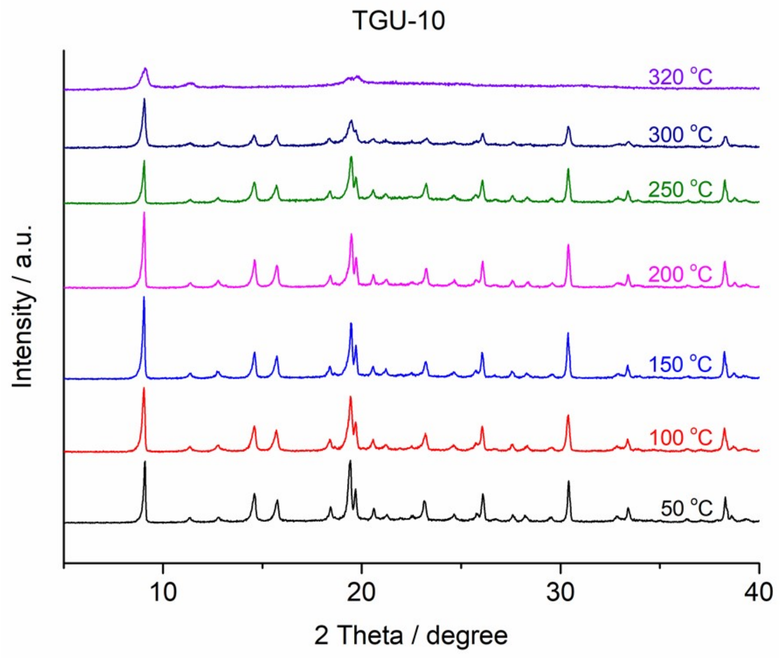

Fig. S45 Temperature-dependent *in-situ* PXRD patterns of TGU-10.

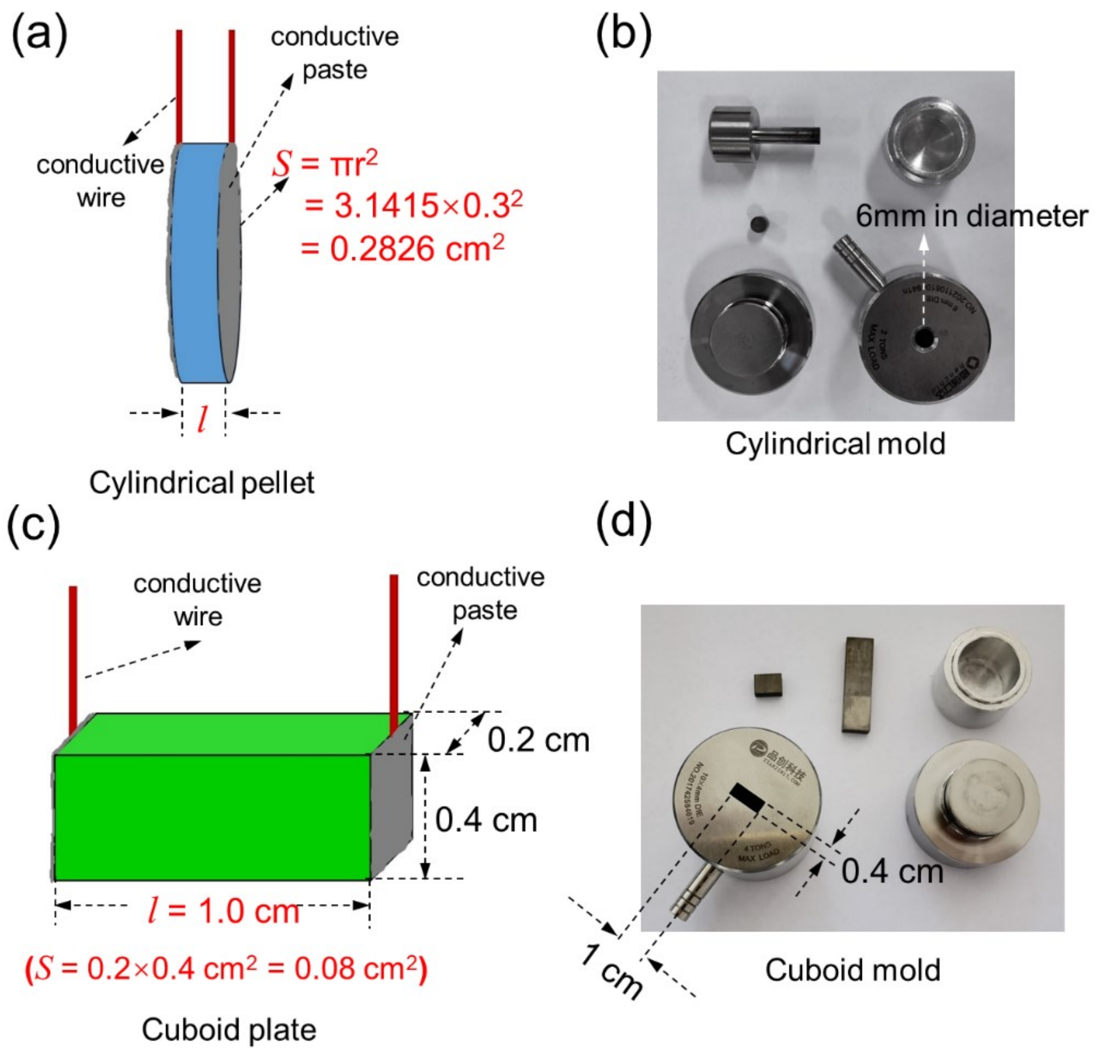

Fig. S46 Schematic representation of the different shape used for impedance test and the photos of corresponding pressing mold. (a), Cylindrical pellet. (b), Cylindrical mold. (c), Cuboid plate. (d), Cuboid mold.

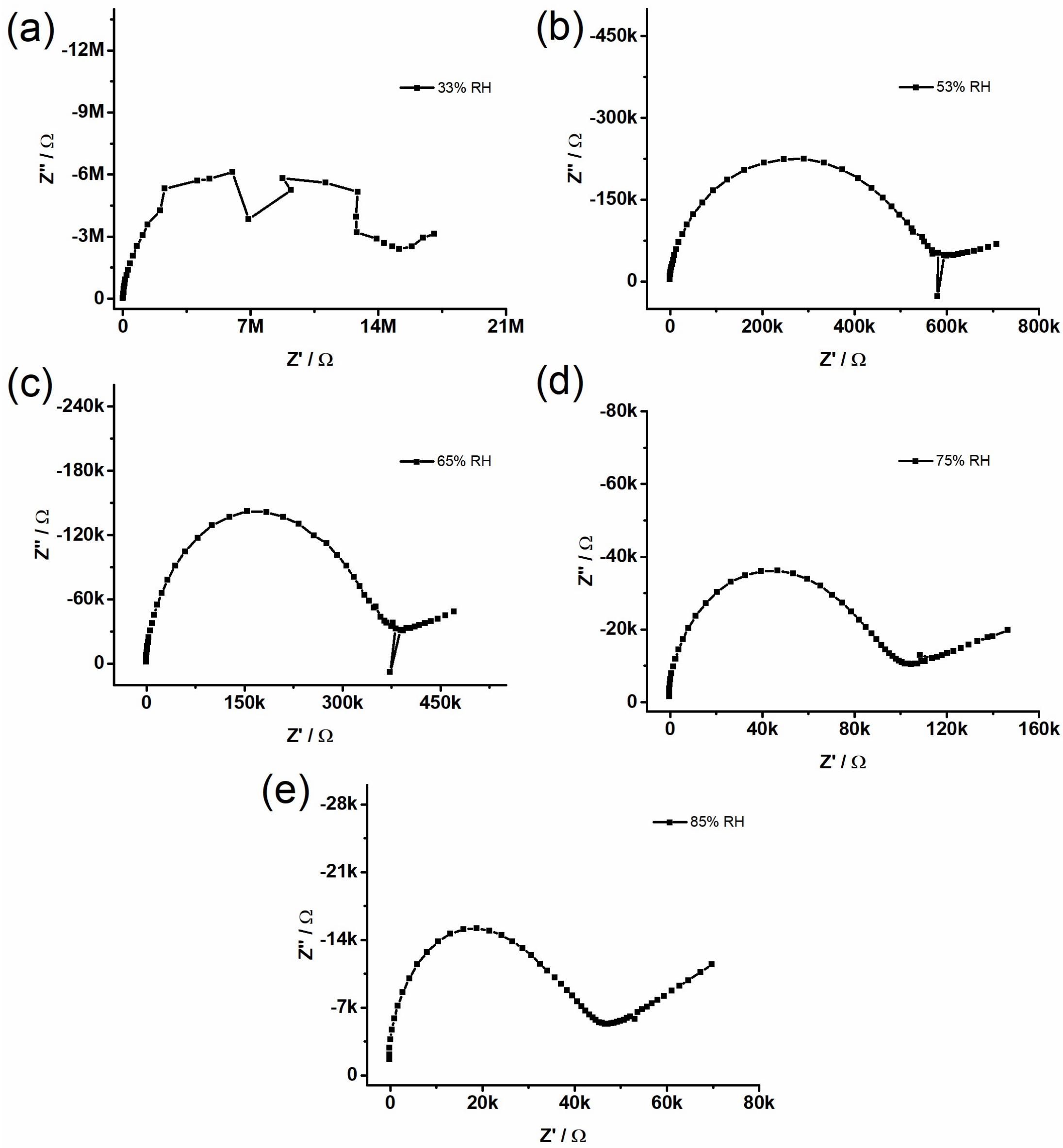

Fig. S47 RH-dependent impedance plots of TGU-9 after stored in air for 1260 days. (a), 33% RH; (b), 53% RH; (c), 65% RH; (d), 75% RH; (e), 85% RH. The cuboid plate of TGU-9 is used to obtained these plots.

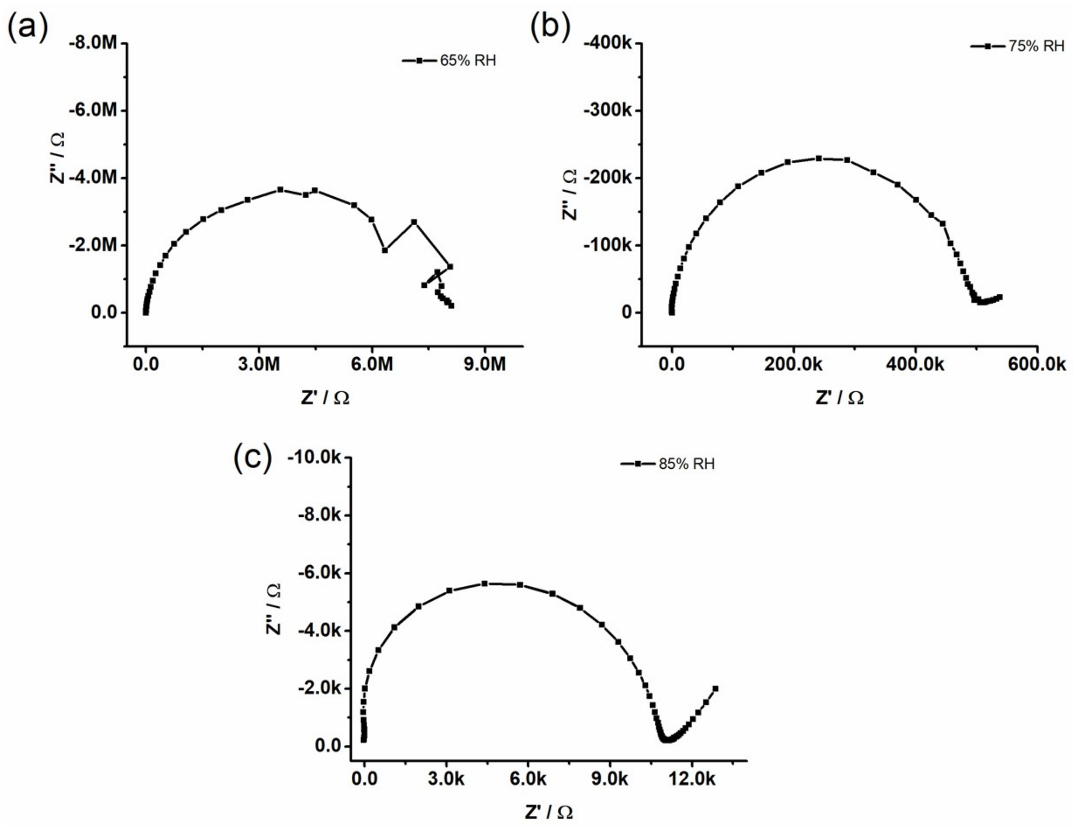

Fig. S48 RH-dependent impedance plots of TGU-10 after stored in air for 200 days. (a), 65% RH; (b), 75% RH; (c), 85% RH. The cylindrical pellet with the thickness of 0.157 cm is used to obtained these plots.

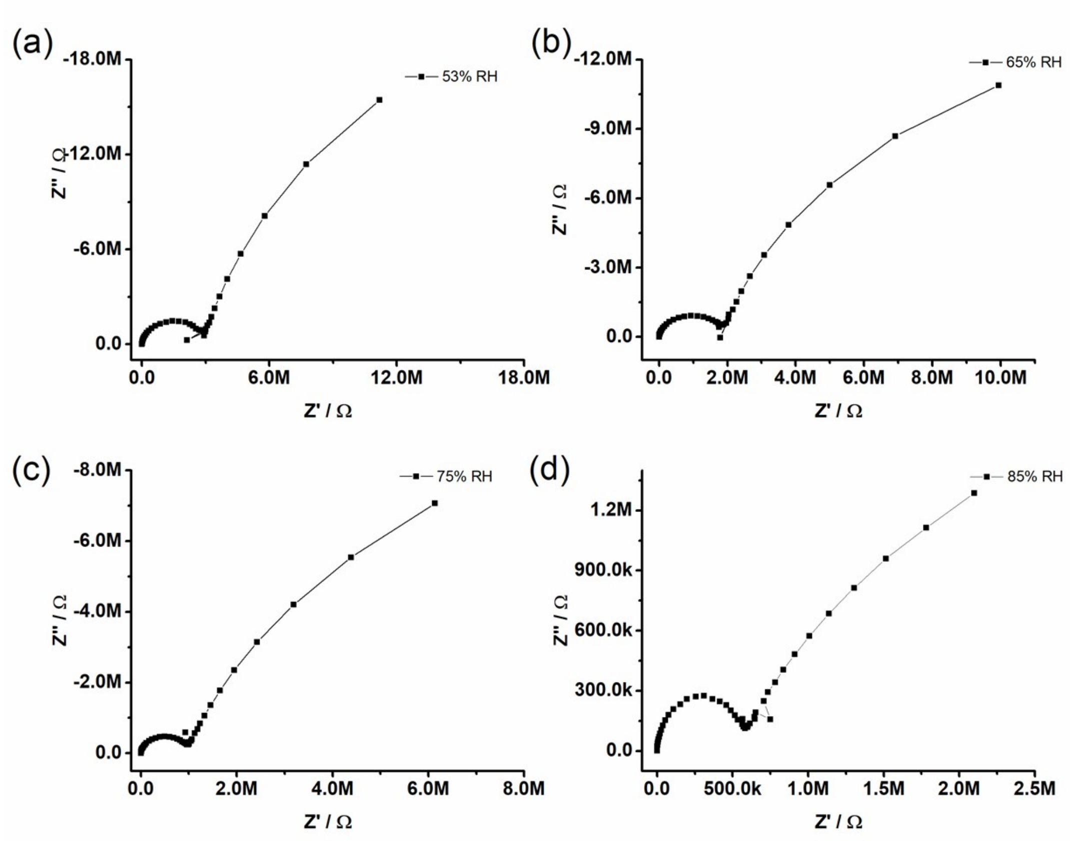

Fig. S49 RH-dependent impedance plots of fresh-made TGU-11. (a), 53% RH; (b), 65% RH; (c), 75% RH; (d), 85% RH. The cylindrical pellet with the thickness of 0.185 cm is used to obtained these plots.

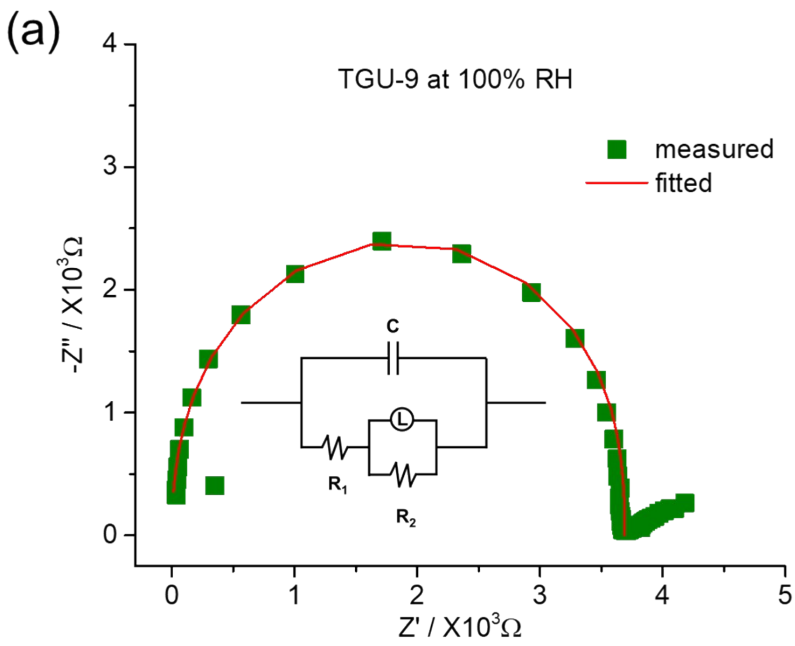

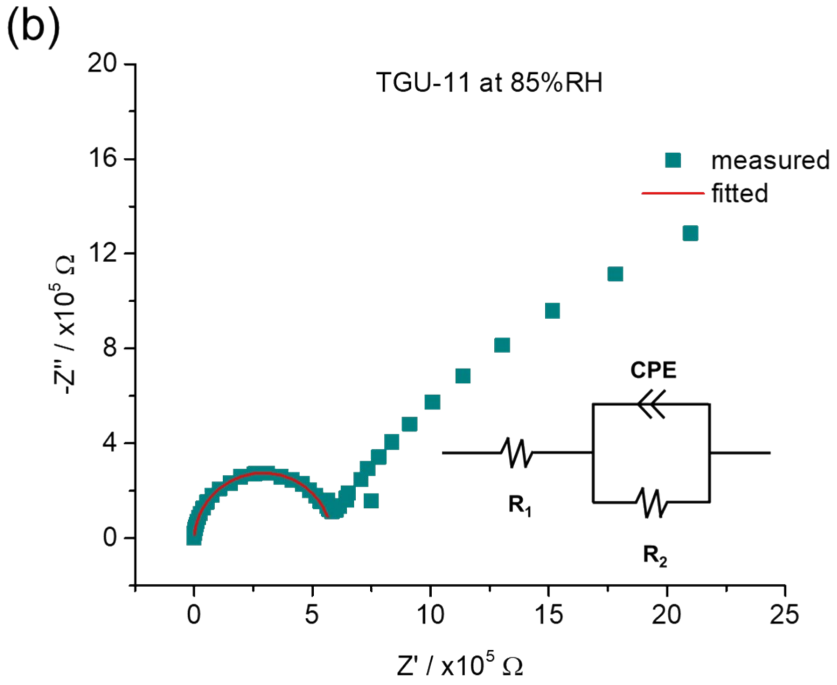

Fig. S50 The impedance plots (dots) and the fitting data (line). (a), the data of TGU-9 at 100% RH; (b), the data of TGU-11 at 85% RH. The insertion in each figure is the corresponding equivalent circuit.

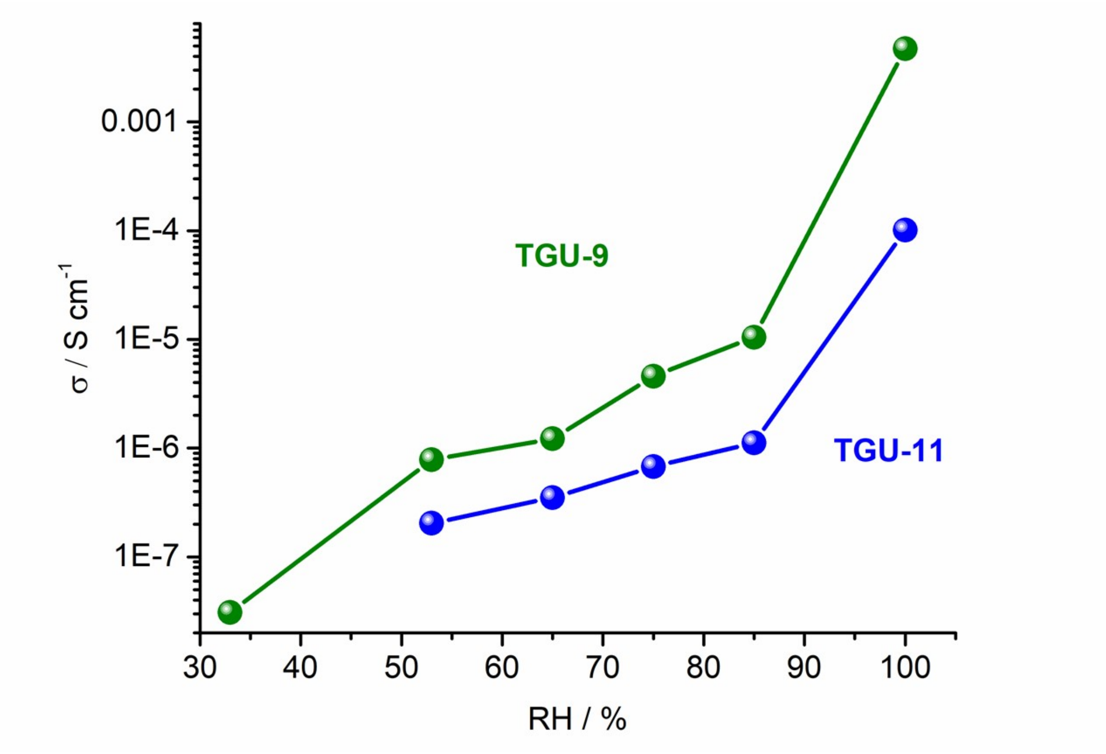

Fig. S51 RH-dependent proton conductivities of TGU-9 and TGU-11 at 25 ^{o}C.

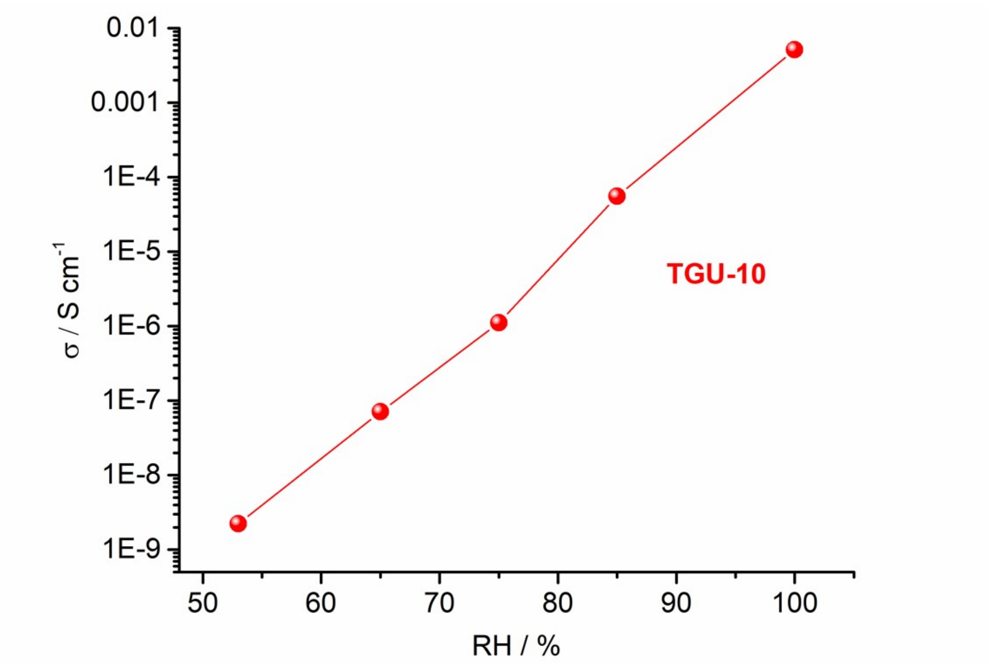

Fig. S52 RH-dependent proton conductivity of TGU-10 at 25 ^{o}C.

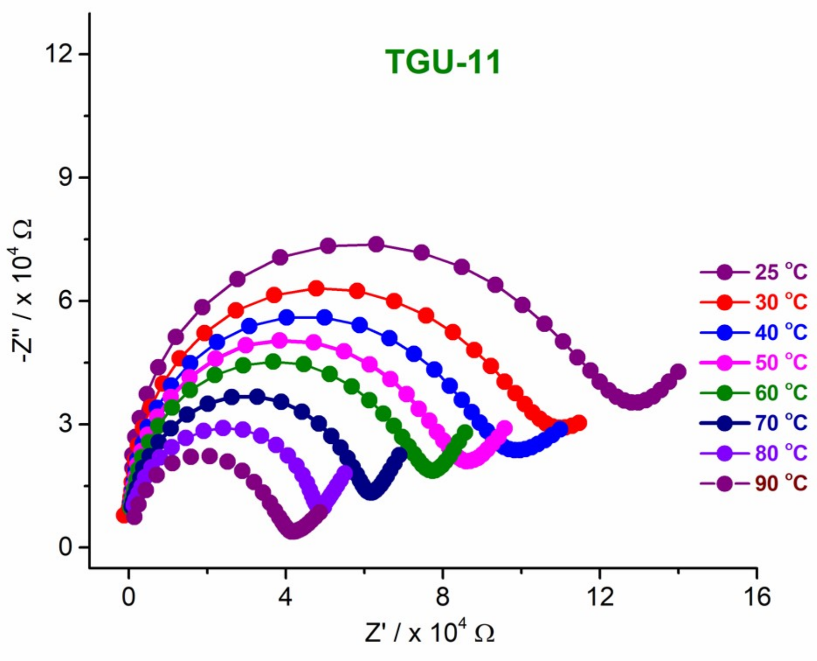

Fig. S53 Temperature-dependent impedance plots of the fresh-made TGU-11 at 100% RH. The cylindrical pellet with the thickness of 0.165 cm is used to obtained these plots.

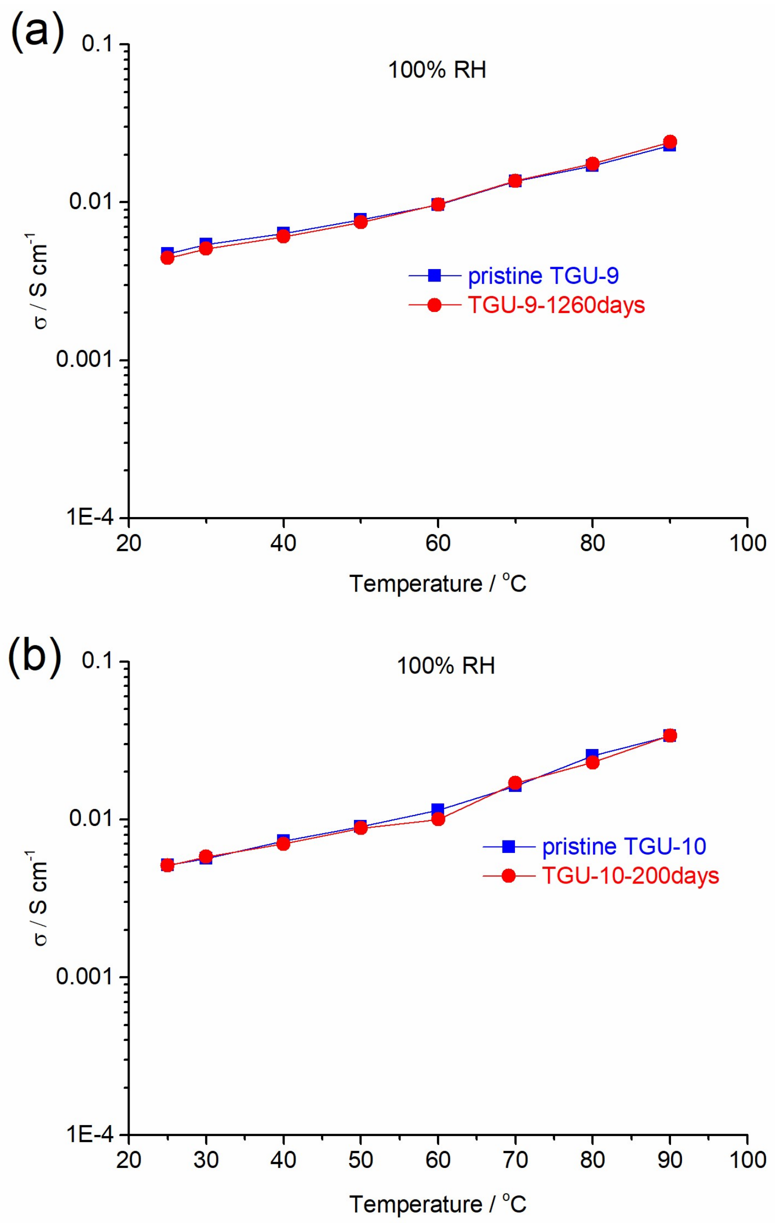

Fig. S54 Temperature-dependent proton conductivities of the fresh-made CPs and that after long-time stored in air. (a), TGU-9; (b), TGU-10.

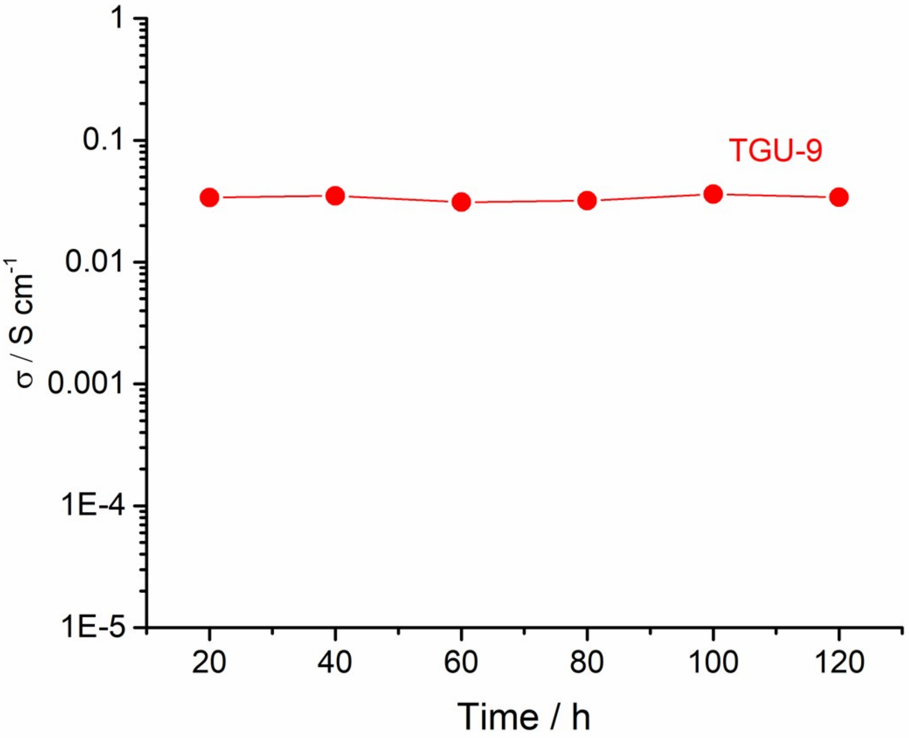

Fig. S55 Time-dependent proton conductivity of TGU-9 at 80 ^{o}C and 100% RH.

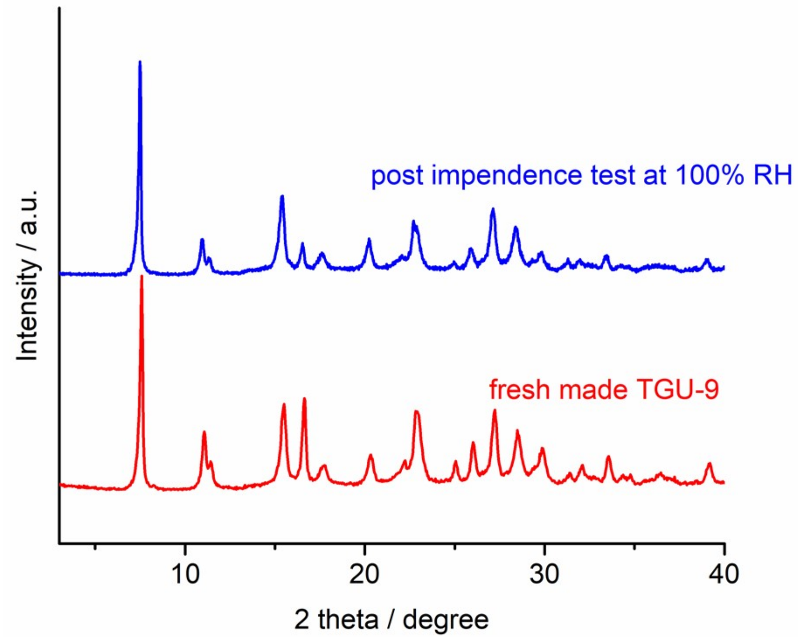

Fig. S56 PXRD patterns of TGU-9 before and after impedance test.

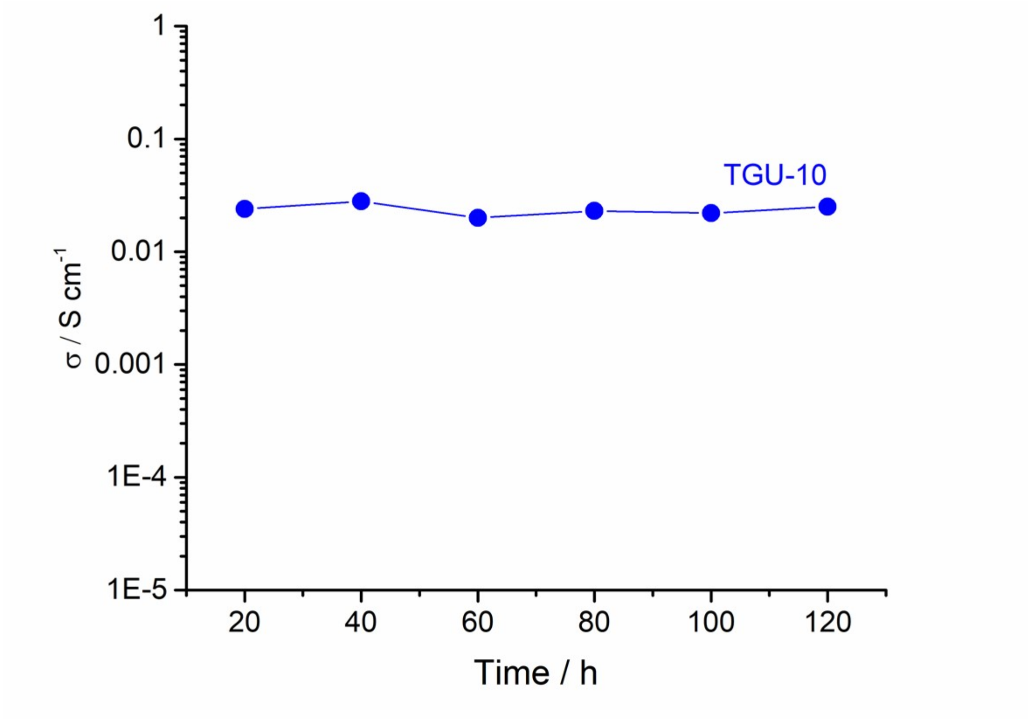

Fig. S57 Time-dependent proton conductivity of TGU-10 at 80 °C and 100% RH.

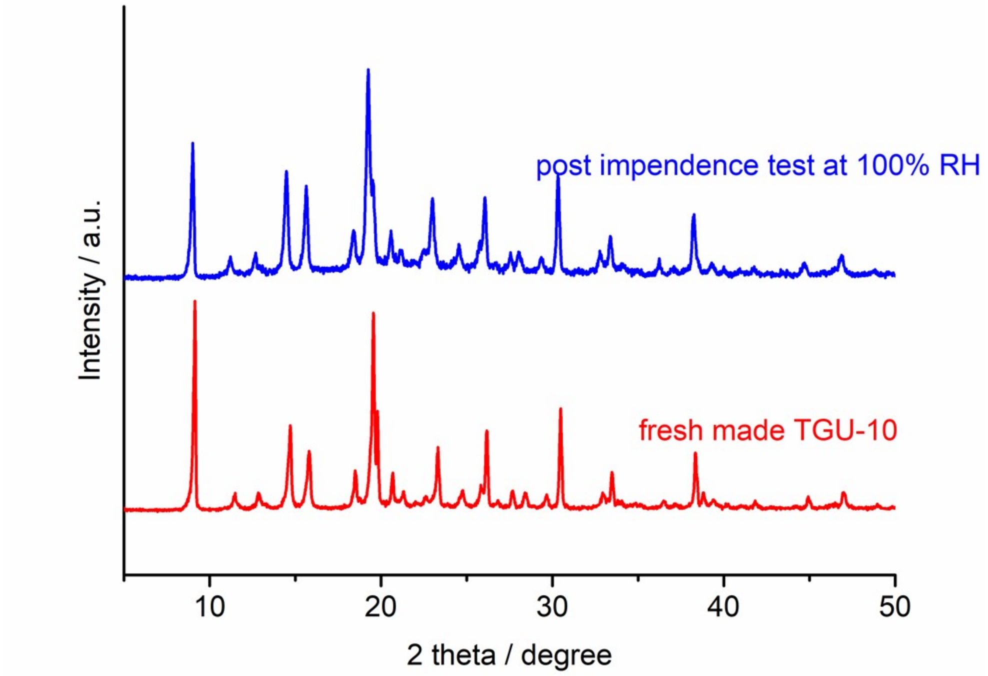

Fig. S58 PXRD patterns of TGU-10 before and after impedance test.

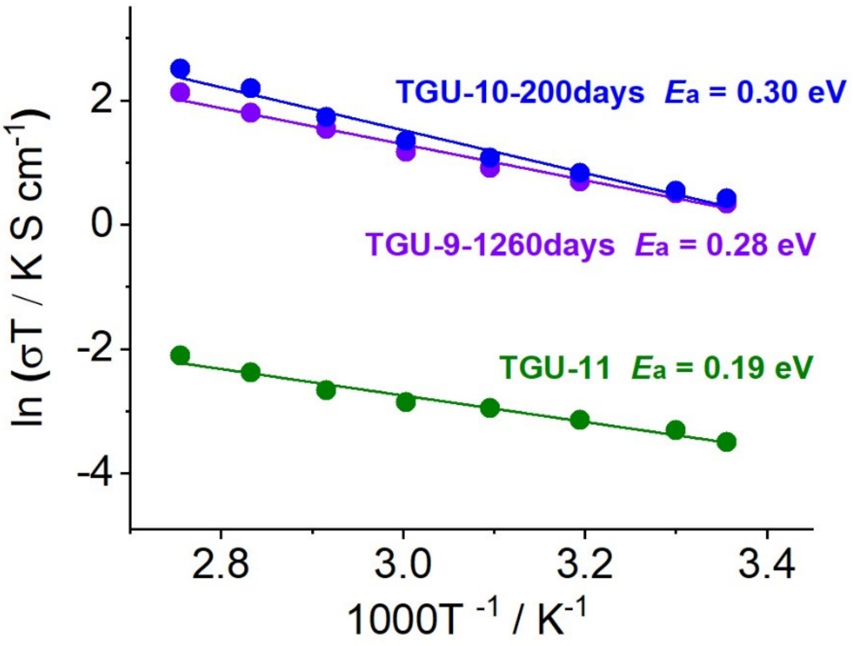

Fig. S59 Arrhenius plots of the long-time stored TGU-9, TGU-10, and fresh-made TGU-11.

Table S5. Proton conduction comparison of TGU-9, TGU-10, TGU-11, and the sulfonate-coordinated CPs.

**CPs**	**Proton conductivity ($S\ cm^{-1}$)**	**E_a / eV**	**Refs.**
TGU-9	**3.5×10^{-2}** (90 °C, 100% RH)	0.28	This work
TGU-10	**3.39×10^{-2}** (90 °C, 100% RH)	0.30	This work
TGU-11	**3.14×10^{-4}** (90 °C, 100% RH)	0.19	This work
$PCMOF2_{1/2}$	2.1×10^{-2} (85 °C, 90% RH)	0.21	11
$[CuH(Hsfpip)Cl(H_2O)]$	1.50×10^{-2} (95 °C, 97% RH)	0.41	12
$[Co_{16}(TPC4R\text{-}I)_2(H_25\text{-}sip)_4(H5\text{-}sip)_8(H_2O)_{24}]\cdot12DMF\cdot4H_2O$	1.35×10^{-2} (90 °C, 98% RH)	1.32	13
$\{[Cu_2(sba)_2(bpg)_2(H_2O)_3]\cdot5H_2O\}_n$	9.4×10^{-3} (80 °C, 95% RH)	0.64	14
Cr/sBDC-Gel-0.4	7.8×10^{-3} (80 °C, 100% RH)	0.3	15
$\{[Er_4(OH)_4(DSOA)_2(H_2O)_8]\cdot4.6H_2O\cdot1.4CH_3CN\}_n$	6.59×10^{-3} (80 °C, 95% RH)	0.32	16
$Cu_2H_2(Hspip)_2Cl_4\cdot H_2O$	6.47×10^{-3} (95 °C, 97% RH)	0.12	17
$[Cu(H_2SNDC)(DMF)_4]_n$	3.46×10^{-3} (95 °C, 95% RH)	0.68	18
Cu-DSOA	1.9×10^{-3} (100 °C, 95% RH)	1.04	19
$[Cp_3Zr_3(\mu_3\text{-}O)(\mu_3\text{-}OH)_3]_2L_3\cdot4Na\cdot H_2O$	1.41×10^{-3} (30 °C, 95% RH)	0.225	20
PCMOF-17	1.25×10^{-3} (25 °C, 40% RH)	0.31	21
JXNU-2(Sm)	1.11×10^{-3} (80 °C, 98% RH)	0.628	22
$Cu_4(5\text{-}sip)_2(OH)_2(DMF)_2$	7.4×10^{-4} (95 °C, 95% RH)	1.32	23
Cu-SAT	5.3×10^{-4} (80 °C, 98% RH)	0.23	24
$[Zn(H5\text{-}sip)(4,4'\text{-}bpy)]\cdot DMF\cdot2H_2O$	3.9×10^{-4} (25 °C, 60% RH)	-	25
Tb-DSOA	1.66×10^{-4} (100 °C, 95% RH)	0.45	26

$[Cu(4,4'\text{-}bpy)_2(1,4\text{-}BDMS)(H_2O)_{0.5}]_n$	1.23×10^{-4} (90 °C, 98% RH)	0.37	27
$[Ba(H_3BPM)(H_2O)]\cdot H_2O$	1.21×10^{-4} (22 °C, 90% RH)	0.54	28
JXNU-7(Eu)	1.04×10^{-4} (85 °C, 98% RH)	0.34	29
$[\{(H_3O)[Eu(SBDB)(H_2O)_2]\}_n]$	1.0×10^{-4} (65 °C, 98% RH)	0.48	30
$[\{In_2(\mu\text{-}OH)_2(SO_4)_4\}\{(LH)_4\}\ nH_2O]_n$	4.4×10^{-5} (30 °C, 98% RH)	0.32	31
$\{[Cd\text{-}(4,4'\text{-}bpe)_{0.5}(5\text{-}sip)(H_2O)]\cdot 4H_2O(4,4'\text{-}H_2bpe)_{0.5}\}_n$	3.7×10^{-5} (65 °C, 95% RH)	0.37	32
$\{[Cu(pyz)(H5\text{-}sip)(H_2O)_2]\ (H_2O)_2\}_n$	3.5×10^{-5} (65 °C, 95% RH)	0.35	33
$Co(dia)_{1.5}(Hsip)(H_2O)\cdot H_2O$	3.461×10^{-5} S cm^{-1} (85 °C, 98% RH)	0.404	34
$(Cs_3(THB)(H_2O)_{3.4}$	1.1×10^{-5} (70℃, 50% RH)	0.3	35
$\{[Cu_2(DBDC)(4,4'\text{-}bpy)_{2.5}(H_2O)]\cdot 1.7H_2O\}_n$	4.48×10^{-7} (65 °C, 95% RH)	0.95	36

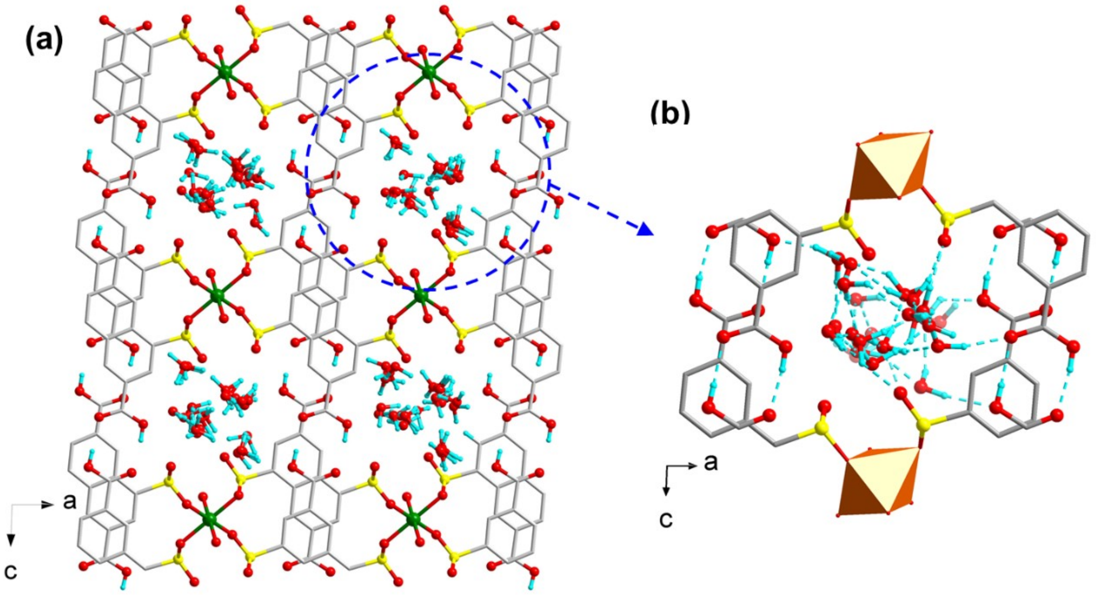

Fig. S60 Simulated water in the channel of TGU-9 with the hydrogen bonding networks. (a), water distribution in TGU-9; (b), the hydrogen bonding networks in one channel.

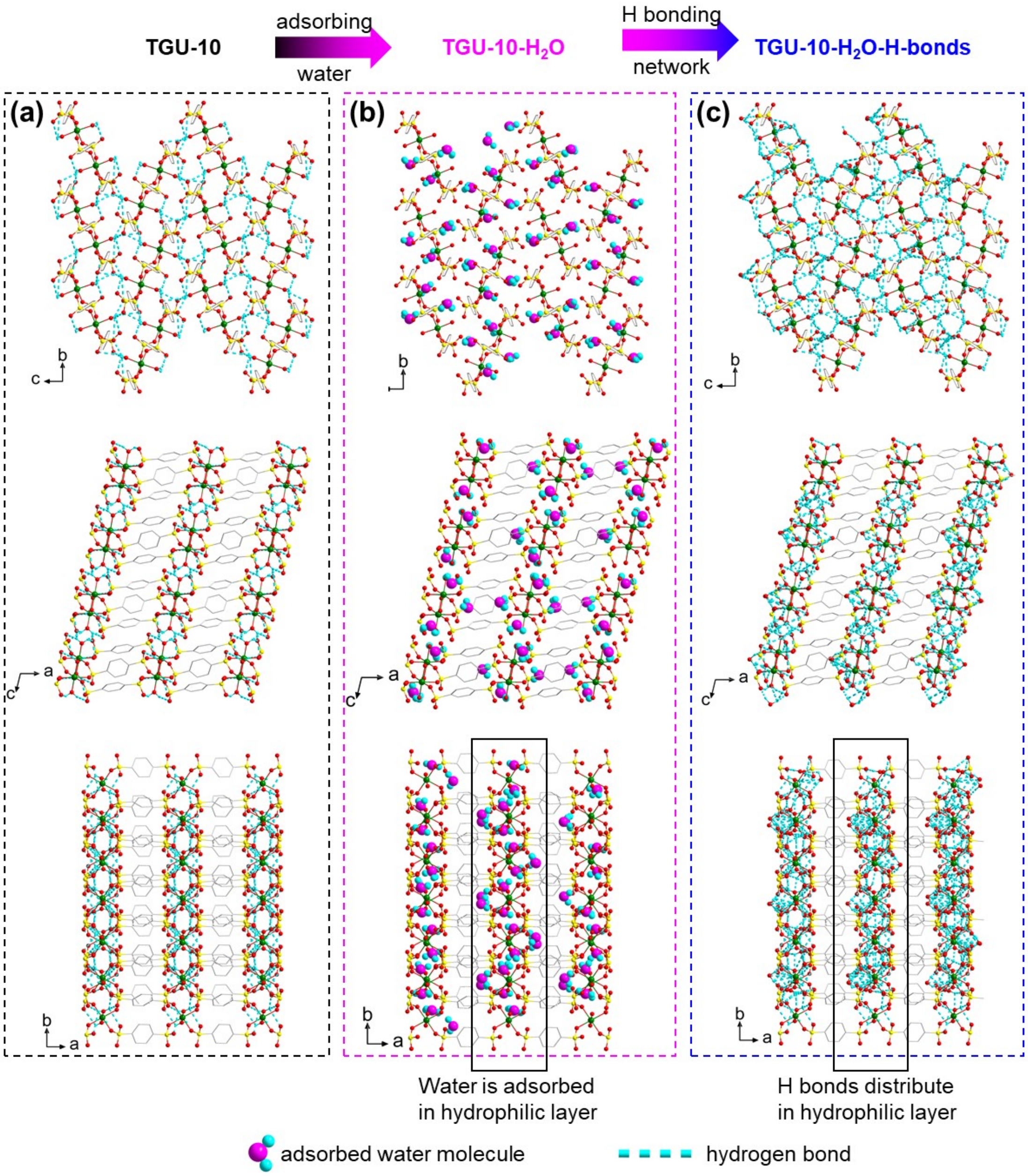

Fig. S61 Comparison of the hydrogen bonding networks in TGU-10 before and after adsorbing water vapor viewed along different directions. (a), the pristine hydrogen bonds in TGU-10; (b), TGU-10 with adsorbed water vapor; (c), hydrogen bonding network in TGU-10 with adsorbed water vapor.

Reference

1. F. Yang, G. Xu, Y. Dou, B. Wang, H. Zhang, H. Wu, W. Zhou, J.-R. Li and B. Chen, Nat. Energy., 2017, 2, 877.
2. A. Mietrach, T. W. T. Muesmann, J. Christoffers and M. S. Wickleder, Eur. J. Inorg. Chem., 2009, 35, 5328.
3. Kabsch, W. XDS. Acta Cryst., 2010, D66, 125.
4. A. A. Coelho, J. Appl. Crystallogr., 2018, 51, 210.
5. W. Wan, J. Sun, J. Su, S. Hovmöller and Zou, X, J. Appl. Cryst., 2013, 46, 1863.
6. G. M. Sheldrick, Acta Cryst., 2015, A71, 3.
7. T. Yang, T. Willhammar, H. Xu, X. Zou and Z. Huang, Nat. Protocols., 2022, 17, 2389.
8. W. L. Jorgensen, J. Chandrasekhar, J. D. Madura, R. W. Impey, M. L. Klein, J. Chem. Phys. 1983, 79, 926–935.
9. A. K. Rappé, C. J. Casewit, K. S. Colwell, W. A. Goddard III, W. M. Skiff, J. Am. Chem. Soc. 1992, 114, 10024–10035.
10. C. E. Wilmer, K. C. Kim, R. Q. Snurr, J. Phys. Chem. Lett. 2012, 3, 2506.
11. S. Kim, K. W. Dawson, B. S. Gelfand, J. M. Taylor, G. K. H. Shimizu, J. Am. Chem. Soc. 2013, 135, 963−966.
12. M. J. Wei, J. Q. Fu, Y. D. Wang, J. Y. GU, B. L. Liu, H. Y. Zang, E. L. Zhou, K. Z. Shao, Z. M. Su, J. Mater. Chem. A 2017, 5, 1085–1093.
13. T. T. Guo, D. M. Cheng, J. Yang, X. X. Xu, J. F. Ma, Chem. Commun. 2019, 55, 6277–6280.
14. S. B. Tayade, R. Illathvalappil, V. Lapalikar, D. Markad, S. Kurungot, B. Pujari, A. S. Kumbhar, Dalton. Trans. 2019, 48, 11034–11044.
15. M. Qiu, H. Wu, L. Cao, B. B. Shi, X. He, H. H. Geng, X. L. Mao, P. F. Yang, Z. Y. Jiang, ACS Appl. Mater. Interfaces 2020, 12, 19788–19796.
16. S. P. Bera, A. Mondal, S. Roy, B. Dey, A. Santra, S. Dalton. Konar, Trans. 2018, 47, 15405.

17. M. J. Wei, J. Q. Fu, Y. D. Wang, Y. Zhang, H. Y. Zang, K. Z. Shao, Y. G. Li, Z. M. Su, CrystEngComm. 2017, 19, 7050–7056.
18. S. N. Zhao, X. Z. Song, M. Zhu, X. Meng, I. I. Wu, S. Y. Song, C. Wang, H. J. Zhang, Dalton Trans. 2015, 44, 948–954.
19. X. Y. Dong, R. Wang, J. B. Li, S. Q. Zang, H. W. Hou, T. C.W. Mak, Chem. Commun. 2013,49, 10590–10592.
20. W. H. Xing, H. Y. Li, X. Y. Dong, S. Q. Zang, J. Mater. Chem. A. 2018, 6, 7724–7730.
21. J. Lin, Z. Romero, G. K. H. Shimizu, B. Joarder, J. Am. Chem. Soc. 2017, 139, 7176–7179.
22. J. Zhao, X. He, Y. Zhang, J. Zhu, X. Shen, D. Zhu, Cryst. Growth Des. 2017, 17, 5524–5532.
23. X. Meng, S. Y. Song, X. Z. Song, M. Zhu, S. N. Zhao, L. L. Wu, H. J. Zhang, Chem. Commun. 2015, 51, 8150.
24. R. Moi, A. Ghorai, S. Banerjee, K. Biradha, Cryst. Growth Des. 2020, 20, 5557–5563.
25. P. Ramaswamy, R. Matsuda, W. Kosaka, A. George, H. J. Jeon, S. Kitagawa, Chem. Commun. 2014, 50, 1144.
26. X. Y. Dong, R. Wang, J. Z. Wang, S. Q. Zang, T. C. W. Mak, J. Mater. Chem. A, 2015, 3, 641–647.
27. G. Zhang, H. Fei, Chem. Commun. 2017,53, 4156–4159.
28. A. Javed, T. Wagner, S. Wöhlbrandt, N. Stock, M. Tiemann, ChemPhysChem. 2020, 21, 605–609.
29. M.Y. Xu, Y. L. Wang, Q. Liu, Z. T. Lin, Q. Y. Liu, Inorganic Chemistry. 2020, 59, 7265–7273.
30. Y. Y. Yuan, S. L. Yang, C. X. Zhang, Q. L. Wang, CrystEngComm 2018, 20, 6989.
31. B. Manna, B. Anothumakkool, A.V. Damod, P. Samanta, S. Kurungot, Ghosh, S. K. Inorg. Chem. 2015, 54, 5366–5371.

32. D. K. Maity, S. Ghosh, K. Otake, H. Kitagawa, G. Debajyoti, Inorg. Chem. 2019, 58, 12943–12953.

33. D. K. Maity, K. Otake, S. Ghosh, H. Kitagawa, D. Ghoshal, Inorg. Chem. 2017, 56, 1581–1590.

34. T.-E. Ho, A. Datta, H. M. Lee. CrystEngComm 2022, 24, 5450–5459.

35. N. Wong, J. A. Hurd, R. Vaidhyanathan, G, K, H. Shimizu, Can. J. Chem. 2015, 9, 988–991.

36. S. Zhang, S. Gao, X. Wang, X. He, J. Zhao, D. Zhu, Acta Cryst B, 2019, 75, 1060–1068.
